# Evidence-based management and motor rehabilitation of cerebral palsy children and adolescents: a systematic review

**DOI:** 10.3389/fneur.2023.1171224

**Published:** 2023-05-25

**Authors:** Silvia Faccioli, Emanuela Pagliano, Adriano Ferrari, Cristina Maghini, Maria F. Siani, Giada Sgherri, Gina Cappetta, Giulia Borelli, Giuseppina M. Farella, Maria Foscan, Marta Viganò, Silvia Sghedoni, Silvia Perazza, Silvia Sassi

**Affiliations:** ^1^Children Rehabilitation Unit, Azienda Unità Sanitaria Locale IRCCS di Reggio Emilia, Reggio Emilia, Italy; ^2^Ph.D. Program in Clinical and Experimental Medicine, Department of Biomedical, Metabolic and Neural Sciences, University of Modena and Reggio Emilia, Modena, Italy; ^3^Neurodevelopmental Unit, Fondazione IRCCS Istituto Neurologico Carlo Besta, Milan, Italy; ^4^Functional Rehabilitation Unit, IRCCS E. Medea, Associazione La Nostra Famiglia, Bosisio Parini, Italy; ^5^Physical Medicine and Rehabilitation Unit, S. Maria delle Croci Hospital, Azienda Unità Sanitaria Locale Romagna, Ravenna, Italy; ^6^Developmental Neuroscience Clinical Department, IRCCS Fondazione Stella Maris, Pisa, Italy; ^7^Physical Medicine and Rehabilitation Unit, Infermi Hospital, Azienda Unità Sanitaria Locale Romagna, Rimini, Italy; ^8^Physical Medicine and Rehabilitation Unit, IRCCS Istituto Ortopedico Rizzoli, Bologna, Italy

**Keywords:** cerebral palsy, rehabilitation, physical therapy modalities, occupational therapy, patient participation, learning, exercise, play and playthings

## Abstract

**Background:**

Evidence regarding the management of several aspects of cerebral palsy improved in recent years. Still, discrepancies are reported in clinical practice. Italian professionals and stakeholders expressed the need of setting up updated, evidenced-based, shared statements, to address clinical practice in cerebral palsy rehabilitation. The objective of the present study was to provide an updated overview of the state of knowledge, regarding the management and motor rehabilitation of children and young people with cerebral palsy, as the framework to develop evidence-based recommendations on this topic.

**Methods:**

Guidelines and systematic reviews were searched, relative to evidence-based management and motor treatment, aimed at improving gross motor and manual function and activities, in subjects with cerebral palsy, aged 2–18 years. A systematic search according to the Patients Intervention Control Outcome framework was executed on multiple sites. Independent evaluators provided selection and quality assessment of the studies and extraction of data.

**Results:**

Four guidelines, 43 systematic reviews, and three primary studies were included. Agreement among guidelines was reported relative to the general requirements of management and motor treatment. Considering the subject's multidimensional profile, age and developmentally appropriate activities were recommended to set individual goals and interventions. Only a few approaches were supported by high-level evidence (i.e., bimanual therapy and constraint-induced movement therapy to enhance manual performance). Several task-specific active approaches, to improve gross motor function and gait, were reported (mobility and gait training, cycling, backward gait, and treadmill), based on low-level evidence. Increasing daily physical activity and countering sedentary behavior were advised. Based on the available evidence, non-invasive brain stimulation, virtual reality, action-observation therapy, hydrotherapy, and hippotherapy might be complementary to task or goal-oriented physical therapy programs.

**Conclusion:**

A multiple-disciplinary family-centered evidence-based management is recommended. All motor rehabilitation approaches to minors affected by cerebral palsy must share the following fundamental characteristics: engaging active involvement of the subject, individualized, age and developmentally appropriate, goal-directed, skill-based, and preferably intensive and time-limited, but suitable for the needs and preferences of the child or young person and their family, and feasible considering the implications for themselves and possible contextual limitations.

## 1. Introduction

Cerebral Palsy (CP) describes a group of permanent disorders of the development of movement and posture, causing activity limitation, which are attributed to non-progressive disturbances that occurred in the developing fetal or infant brain. The motor disorders of cerebral palsy are often accompanied by disturbances of sensation, perception, cognition, communication, and behavior; epilepsy; and secondary musculoskeletal problems (1). It is the most common motor disability in childhood, affecting 2–2.5 per 1,000 live births (2). Although in CP the causative brain damage is static, the secondary musculoskeletal problems and motor manifestations change over time. Pathological movements and postures manifest during infancy or early childhood, and secondary disability may be progressive and may involve different aspects of the subject's life. Therefore, several specialists and experts are involved in the management of cerebral palsy, and their engagement may change over time. Recommendations were published in the past predominantly covering specific aspects (e.g., botulinum injections and osteoporosis management), then national institutes started promoting clinical practice guidelines, to orient clinical choices and health policies. Italian guidelines for CP were first published in 2005 (3) and then revised in 2012–2014 (4). They provided a comprehensive approach to the complexity of the child's disability profile. Nonetheless, general criteria were reported to guide and coordinate professionals, without specifying proven effective interventions. Evidence regarding the rehabilitation of several aspects of cerebral palsy dramatically improved in recent years. Still, discrepancies are reported in clinical practice, partially due to organizational characteristics and resources of service providers. Guidelines must define what is currently regarded as a safe and appropriate approach. Therefore, Italian stakeholders expressed the need of setting up updated, evidenced-based, shared statements, to address clinical practice in CP rehabilitation. The objective of the present study was to provide an updated overview of the state of knowledge, regarding the management and motor rehabilitation of children and young people with CP, as a framework to develop evidence-based recommendations on this topic.

## 2. Materials and methods

### 2.1. Search and selection

The scope of the systematic review was structured in research questions, according to the Patients, Intervention, Control, and Outcome (PICO) framework. The following queries were considered:

- Which are the general principles to provide comprehensive management of CP subjects under the age of 18 years?
- Which are the most effective motor rehabilitation approaches to improve gross motor or upper limb performance, in CP subjects aged 2–18 years?

Query 2 was deliberately maintained inclusive, rather than providing separated queries for gross motor or manual functions and activities because several studies involved both aspects as outcomes.

Available evidence on each question was systematically enquired. Search and selection procedures are described in the Supplementary material 1.

Clinical practice guidelines (CPGs) were first searched, relative to CP management and rehabilitation. In case of missing or incomplete evidence, to answer the identified queries, the search was extended to systematic reviews (SRs). Screening and selection were independently executed by two evaluators (SG and SS), by first assessing titles and abstracts and then full texts. Any discrepancies among the evaluators were resolved through discussion. A few studies were included from manual search, relative to uncovered topics.

### 2.2. Quality assessment

Two evaluators for each study independently provided the quality assessment of the included documents (SF, SG, SP, and SS). Any discrepancy among the evaluators was resolved through discussion. CPGs were assessed using the Appraisal of Guidelines Research and Evaluation (AGREE) 2 tool (5). Three qualitative levels were identified based on the AGREE 2 scores: “high”, “moderate”, and “low” (6). SRs were assessed using the Assessing the Methodological Quality of Systematic Reviews (AMSTAR) 2 tool (7). While computing the total score, the reviewers agreed in considering “yes partially” as “yes” and item 11 (relative to the meta-analysis) as a non-critical item, because just a minority of studies included a meta-analysis. GRADE's (Grading of Recommendations, Assessment, Development, and Evaluation) evidence profiles were implemented including the few meta-analyses available (8, 9). Observational primary studies were assessed using the Joanna Briggs Institute (JBI) critical appraisal checklist for case series (10).

### 2.3. Data extraction

All authors, in numbers of two for each study, independently provided data extraction, resolving any discrepancy through discussion. Recommendations relevant to the queries were extracted from each selected CPG and reported verbatim. Relevant contents were extracted from the included SRs: population (type of CP and age), characteristics of the intervention, outcome measures, and conclusion of the authors about the effectiveness with adverse events whenever reported. In most cases, studies presented mixed neuromotor treatments, addressing manual or gross motor performances and mixed outcome measures. The extracted contents were synthetized and ordered considering first the essential requirements shared by the child-focused therapies, then considering the individual approaches and addressing manual or gross motor function and activities.

## 3. Results

Based on the search on organizational websites, four CPGs were found: two from the National Institute for Health and Care Excellence (NICE) site (11, 12) and one from the New South Wales (NSW) Ministry of Health site (13), which were included concerning both queries; one report from Haute Autorité de Santé (HAS) site (14) was excluded because only general information was given, irrelevant with respect to the queries.

Concerning the Pubmed search for CPGs, a first selection, based on title and abstract, excluded 363 studies as non-pertinent. Seven were selected and examined on full text, with the exclusion of five as non-pertinent relative to the intervention (15–19). Shaunak et al. (20) reported about NICE CPG, without reporting any further information: therefore, it was finally excluded. Only one CPG by Castelli et al. (4) was finally selected.

**Figure 1:**
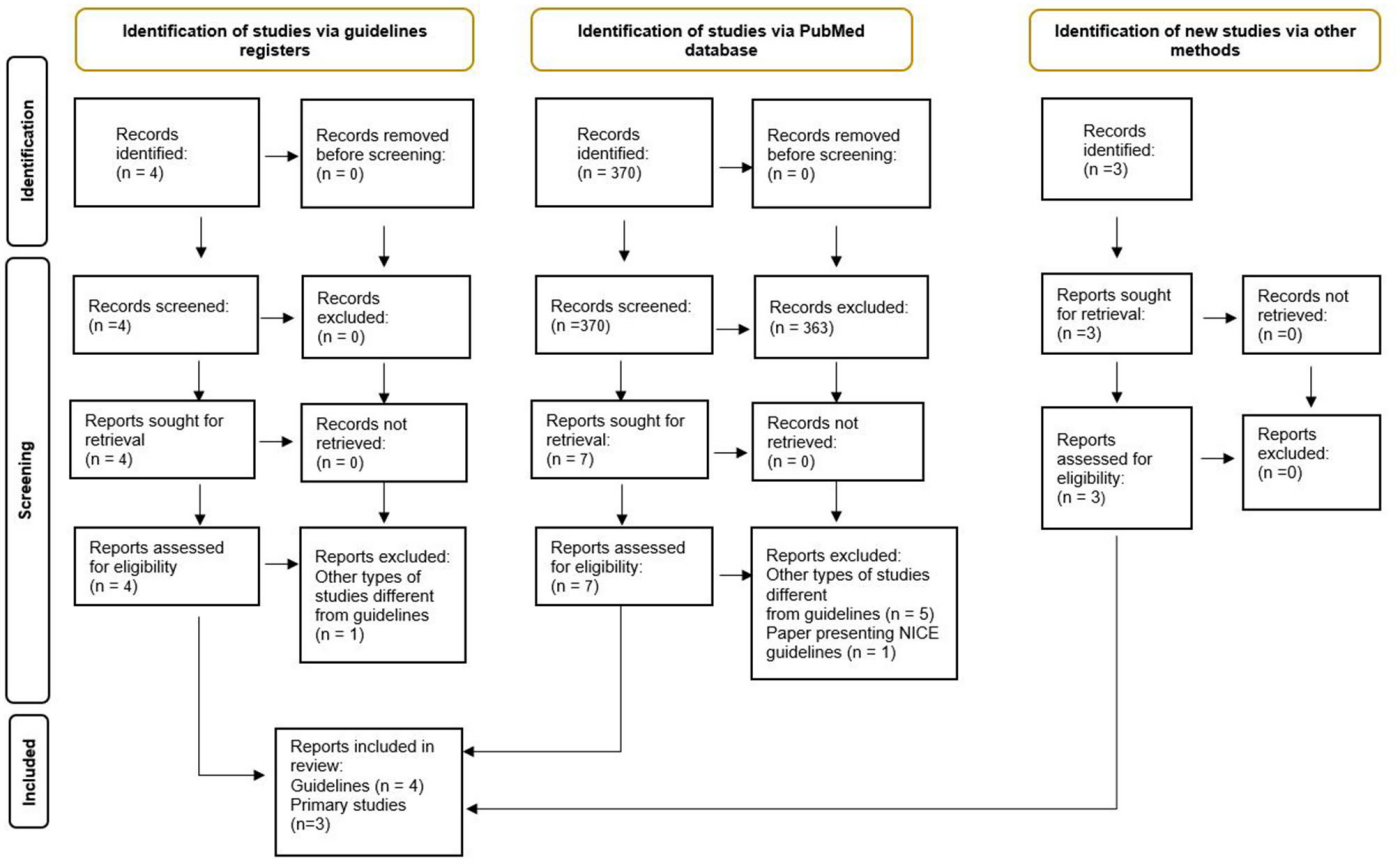
PRISMA flow diagram relative to query 1.

In total, four CPGs were included. They provided exhaustive information relative to query 1. Nonetheless, three primary studies were manually retrieved and included (21–23) to disclose the reference developmental trajectories relative to the functional classifications recommended by the CPGs. Figure 1 represents the PRISMA flowchart relative to Query 1.

**Figure 2:**
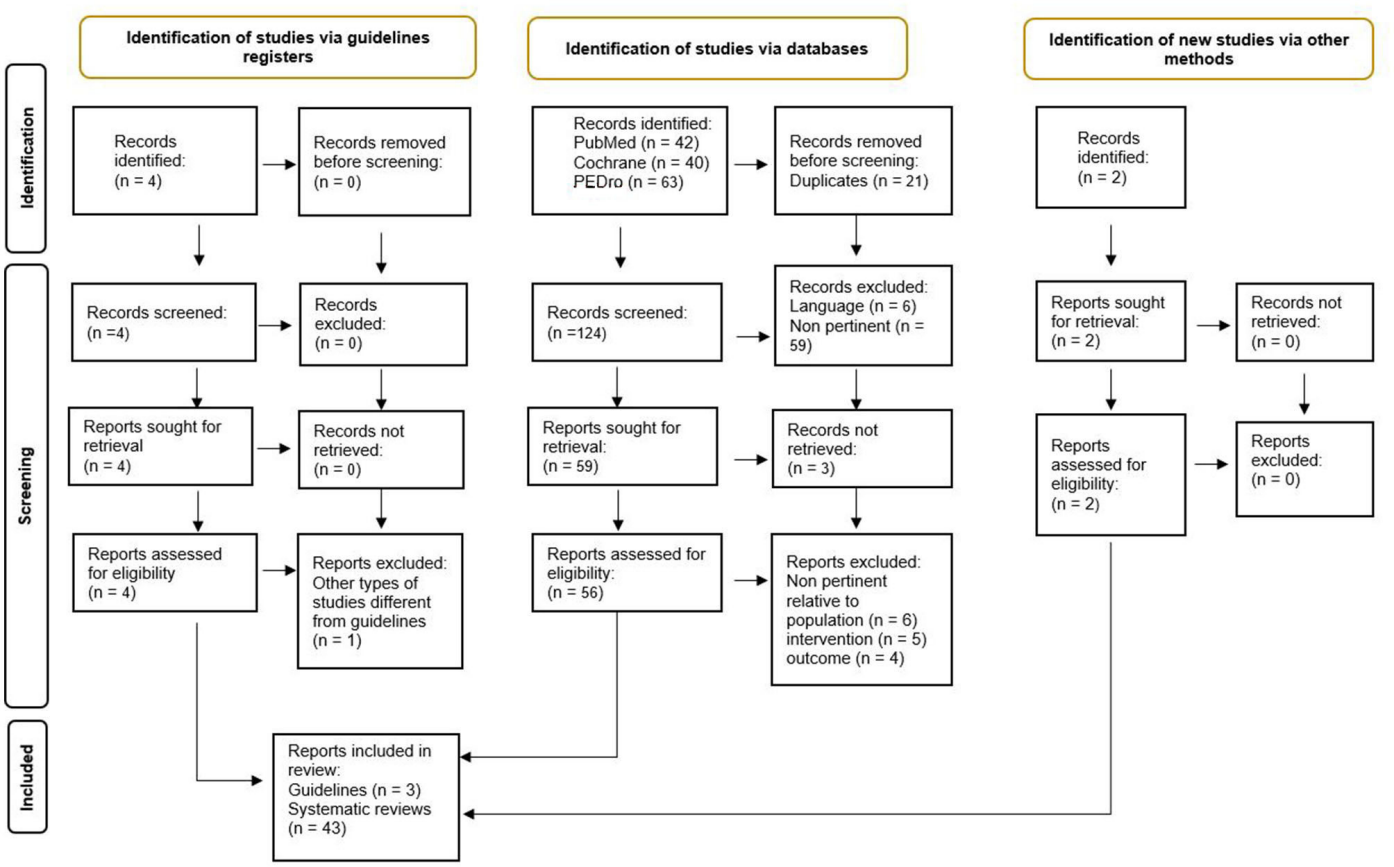
PRISMA flow diagram relative to query 2.

Based on the search on databases for query 2, a total of 145 SRs were retrieved, and after the removal of 21 duplicates, SRs were screened: 65 were excluded on abstracts, three were not retrieved as full text, and 15 were excluded on full text (24–38). Further two SRs were considered from individual search (39, 40). Therefore, 43 SRs (39–81) were finally included, concerning query 2. Figure 2 represents the PRISMA flowchart relative to Query 2.

The quality and risk of bias analysis of included studies are represented in Table 1 for CPGs, Table 2 for observational studies, and Supplementary Table 3 for SRs.

**Table 1:** Quality assessment through AGREE II of included guidelines.

**AGREE II**								
**References**	**Domain 1**	**Domain 2**	**Domain 3**	**Domain 4**	**Domain 5**	**Domain 6**	**TOT**	**Quality** ^*^
	**Scope and purpose**	**Stakeholder involvement**	**Rigor of development**	**Clarity of presentation**	**Applicability**	**Editorial independence**		
Spasticity in under 19s: management National Institute for Health and Care Excellence *(NICE guidelines, 2012-2016)*	97.92%	91.67%	97.92%	97.22%	93.75%	87.50%	95.29%	High quality
Managing cerebral palsy in under 25s *(NICE guidelines, 2021)*	100%	91.67%	98.96%	77.78%	83.33%	87.50%	91.67%	High quality
Management Of Cerebral Palsy In Children: A Guide For Allied Health Professionals *(NSW Ministry of Health guidelines, 2018)*	100%	94.44%	86.46%	97.22%	87.50%	87.50%	90.94%	High quality
SIMFER-SINPIA Intersociety Commission. Recommendations for the rehabilitation of children with cerebral palsy *(Eur J Phys Rehabil Med, 2016)*	91.67%	80.56%	28.13%	58.33%	18.75%	45.83%	47.10%	Low quality

^*^High quality, CPGs that scored ≥60% in at least three of six AGREE II domains, including Domain 3; Moderate quality, CPGs with three AGREE II domains assessed a score of ≥60%, except Domain 3; Low quality, CPGs that scored < 60% in two or more domains and scored < 50% in Domain 3.

**Table 2:** Quality assessment of included primary studies through JBI critical appraisal checklist for case series.

**Primary studies**	**Items**										**Quality**
	**1**	**2**	**3**	**4**	**5**	**6**	**7**	**8**	**9**	**10**	
Rosenbaum et al. (21)	Yes	Yes	Yes	Yes	Yes	Yes	Yes	Yes	Yes	Yes	High
Klevberg et al. (22)	Yes	Yes	Yes	Yes	Yes	Yes	Yes	Yes	Yes	Yes	High
Eliasson et al. (23)	Yes	Yes	Yes	Yes	Yes	Yes	Yes	Yes	Yes	Yes	High

### 3.1. Evidence synthesis relative to query 1

Evidence synthesis concerning query 1 is reported in Supplementary Table 4.

NICE CPG (12) recommended providing a management program developed and implemented in partnership with the child or young person and their parents or careers, individualized and goal-focused. Considering the impact on the individual child or young person and their family was advised. Assessments and goals should be identified, as age and developmentally appropriate, in agreement with the subjects and their parents or careers, focusing on the following domains of the World Health Organization's International Classification of Functioning, Disability, and Health: body functions and structures, activities and participation, environmental factors. The physical therapy (physiotherapy and/or occupational therapy) program had to be tailored to the child or young person's individual needs and aimed at specific goals, such as: enhancing skill development, function, and ability to participate in everyday activities; and preventing consequences such as pain or contractures. The likelihood of achieving the treatment goals, possible difficulties in implementing the program, and implications for the person and their careers had to be considered. Moreover, the CPG recommended reassessing the physical therapy program at regular intervals to ensure the goals are achieved and the program remains appropriate to the child or young person's needs. Finally, ensuring access to adults' services nearby, with expertise in managing cerebral palsy, was reported as a minimum standard of care (11).

The Australian CPG (13) outlined the need to develop individually tailored treatment plans and to provide a multiple-disciplinary team approach (whether in a format of multidisciplinary, interdisciplinary, or transdisciplinary). This was supposed to be locally implemented whenever possible or seeking multidisciplinary support from tertiary institutions or specialist services, to facilitate the provision of a holistic approach. The team was recommended to include all professionals involved in the child's care, as well as the teachers. Australian CPG (13) advocated particular attention to times of transition, with early forward planning to be essential for positive outcomes. Finally, the authors recommended using functional motor ability classification scales to guide assessment, goal setting, and intervention:

- Gross Motor Function Classification System (GMFCS) for the posture-kinetic organization (82).
- Manual Ability Classification System (MACS) relative to praxis manual function (83).
- Communicative Function Classification System (CFCS) for communicative skills (84).
- Eating and Drinking Function Classification System (EDACS) relative to feeding (85).
- Visual Function Classification System (VFCS) concerning visual impairment (86).

Previous Italian recommendations (4) described rehabilitation as a complex process focusing on the person in all his/her dimensions, physical, mental, emotional, communicative, and relational (holistic approach), and involving the child's family and social and environmental context (ecological approach). Interventions were supposed to be tailored based on the patient's profile, considering his/her functioning in the area of autonomic control, personal autonomy, locomotion, manipulation and praxis, sensation/perception, cognition, communication, and relationships; the architecture of the main functions (activities/abilities); and age and developmentally appropriate goals. While assessing the patient, the authors recommended taking into account not only the single functional area involved but also its relationship with the other areas, to be able to define the overall level of development attained and the reciprocal impact between areas. It was considered important to provide not just a mere description of the skills (i.e., present, absent, or emerging) but also to state whether and in what way the child implemented adaptive, compensatory, or additional strategies, not least because these could serve as a crucial guide for the proposed therapy.

Considering the recommendation by the Australian CPG (13) to refer to functional classifications, the authors agreed on the relevance of integrating with the developmental curves based on the cited classifications. They appeared particularly important to focus on the critical periods and to define the limits of rehabilitation. Rosenbaum et al. (21) reported the gross motor development curves of 657 uni- and bilateral CPs, of mixed subtypes, describing average development predicted by the Gross Motor Classification System. The age range was 1–13 years at first assessment and the follow-up lasted 4 years. Higher ability levels reached their limit of development in a longer period than lower ability levels, though all levels reached their developmental limit by the age of 7 years.

Klevberg et al. (22) described the bimanual performance of 60 unilateral and 42 bilateral CP of mixed subtypes, MACS I–III. The mean age at the first assessment was 25 months for unilateral and 35 months for bilateral CP. The mean follow-up was 4.5 months. Children with bilateral CP seemed to reach their developmental limits around 30 months of age, regardless of MACS level, and to change their performance over time to a smaller extent than those with unilateral CP.

Eliasson et al. (23) confirmed that the Assistive Hand Assessment (AHA) score at 18 months together with the MACS levels resulted in the prediction of future development, based on data from 171 unilateral spastic CP, with an age range of 18 months−18 years and mean follow-up of 8 years. Children classified as having higher ability (MACS level I) had both a higher rate and limit of development and a shorter period of development than those having a lower ability (MACS level II). Children functioning in MACS level III had the lowest limit, and development occurred for the longest time. The stable performance lasted throughout adolescence for participants in all MACS levels, from approximately 7 years. Nonetheless, on an individual level, a large variation in development was seen; therefore, regular follow-up for children in all MACS levels was recommended.

### 3.2. Evidence synthesis relative to query 2

Evidence synthesis concerning query 2 is reported in Supplementary Table 5.

Considering the few meta-analysis available, separate evidence profiles were implemented for the following outcome: gross motor function measured by Gross Motor Function Measure (GMFM) and gait speed; balance measured by Pediatric Balance Scale (PBS), Berg Balance Scale (BBS), or mixed outcome measures; and upper limb performance by AHA, Melbourne assessment of Unilateral Upper Limb function (MUUL), ABILHAND-Kids, or mixed outcome measures (Supplementary Tables 6A–D). The evidence level according to GRADE was overall at low to very low.

Most studies were systematic reviews, without meta-analysis, considering mixed outcome measures and in many cases also mixed characteristics concerning population and treatments. Therefore, a statistical synthesis was not feasible, and a description of the main contents is provided below and in Supplementary Table 5.

#### 3.2.1. Child-focused therapy (goal and task-oriented training) and context-focused therapy

CPGs and SRs agreed in recommending child or context-focused approaches: distinctive features, with time-related references, were discussed.

As previously reported, NICE CPG (12) recommended setting individually tailored goals and interventions, considering age and developmentally appropriate activities, preferences, and impact on the child or young person and their careers. Task-focused active-use therapy, such as constraint-induced movement therapy followed by bimanual therapy, was recommended, to enhance manual skills. An intensive program over a short time (for example, 4–8 weeks) was considered preferable.

The Australian CPG (13) recommended both goal-directed and context-focused therapy to improve function. The first required goals to be age and developmentally appropriate and child-focused to increase motivation. The task should then be analyzed, considering the child's skills as well as environmental limitations, to identify the goal-limiting factor(s). The intervention should be structured and involve repetitive practice, appropriate adaptations to the task or the environment, and outcomes evaluated using validated tools. The context-focused therapy consisted of changing the task or the environment (but not the underlying body structure and function of the child) to promote successful task performance.

Jackman et al. (41) analyzed 74 randomized controlled trials (RCTs) or quasi-RCT, involving CP or high-risk CP subjects, aged 0–18 years. Authors examined the effectiveness of several types of active interventions, classified as “goal-directed”, “functional or part-task”, or “non-functional”. Outcome measures were AHA and Canadian Occupational Performance Measure (COPM). Differently from non-functional approaches, both goal-directed and functional training were presented as effective, but a difference in the “dose” of practice was reported. Interventions that set functional goals and involved the actual practice of those goals led to goal achievement at a lower dose than general upper limb motor training. According to the authors, children were likely to achieve individual goals, if they had set their own goals and had practiced those goals for more than 14 to 25 h, combining face-to-face therapy with home practice. To improve motor ability, a higher dose of practice was needed, likely 30 to 40 h of practice. Moreover, where the outcome was measured on the AHA, logistic regression showed that children under 8 years of age were two times more likely to succeed. On the COPM, results were similar regardless of age, although children over 8 years were 1.46 times more likely to succeed.

Novak et al. (42) updated the previous SR (87) which included five RCTs, with one SR, involving CP subjects of 4–18 years of age, at GMFCS levels I–III. The authors confirmed the effectiveness of goal-oriented training in improving goal achievement of functional tasks involving gross motor, hand function, and self-care. Relative to task-oriented training, the authors included in the analysis only two small RCTs (GMFCS levels I–III, age 4–18 years) that conferred improved gross motor skills compared to control non-task-based therapy. Finally, based on three RCTs (GMFCS levels I–IV, 11 months−4 years), the authors reported no between-group differences for context-focused vs. child-focused to improve self-care. Therefore, they recommended using both approaches simultaneously and letting the family select the preferred one.

Inamdar et al. (43) examined 12 RCTs, involving uni-bilateral CP, at GMFCS levels I–V, and the age range of 18 months–puberty. They concluded that task-specific, intensive, and child-initiated intervention components showed promise for improving sitting in young infants at risk for CP. And components of impairment remediation combined with functional balance training should be explored to improve sitting in children diagnosed with CP.

Hsu et al. (44) included 13 RCTs (GMFCS levels I–III, 1–17 years). The meta-regression analysis revealed that the improvement in GMFM scores was positively associated with the number of daily training hours and program duration.

Das et al. (45) based on 34 SRs, involving mostly hemiplegic CP (0–18 years), confirmed the effectiveness of intensive activity-based, goal-directed interventions. Conversely, the ability of manual stretching to increase the range of motion and reduce spasticity was limited.

#### 3.2.2. Bimanual therapy and hand-arm bimanual intensive therapy

NICE CPG (12) simply recommended considering task-focused active-use therapy, such as constraint-induced movement therapy (CIMT, temporary restraint of an unaffected arm to encourage use of the other arm) followed by bimanual therapy (unrestrained use of both arms) to enhance manual skills.

The Australian CPG (13) recommended bimanual training as an increased opportunity to practice bilateral activities to improve the use of both hands during activity. Bimanual training should provide practicing the specific task or goal, or parts of the task, rather than focusing on the underlying body structure and functional deficits. The best candidates for bimanual training were considered to be older than 12 months, have spontaneous use of affected hand and selective motor control, have basic skills such as grasp and hold, and have the cognitive skills to respond to cues. The effectiveness of bimanual therapy was equal to that of CIMT when the same amount of therapy was provided.

Alahmari et al. (46) considered four RCTs, about bimanual therapy (duration of intervention 60–90 h for 2–4 weeks) in hemiplegic subjects. A meta-analysis on the efficacy (measured using the Jebsen-Taylor Hand Function Test—JTHFT) of HABIT vs. CIMT or structured and unstructured bimanual therapies was conducted: HABIT showed a trivial effect compared to the other interventions, with an effect size of 0.06. Both groups performed functional tasks improving hand function within enjoyable and playful activities.

Ouyang et al. (47) included a SR of 11 RCTs, one quasi-RCT, one retrospective, and two longitudinal studies. The treatments were individualized training, group-based training, or both, mostly in daily camp settings, for hemiplegic subjects aged 3–18 years. The outcome measures were mixed: AHA, JTHFT, Quality of Upper Extremity Skills Test (QUEST), ABILHAND-Kids, Box and Block test (BBT), COPM, and Pediatric Evaluation of Disability Inventory (PEDI). HABIT in the form of 6 h a day for 3 consecutive weeks (totaling 90 h) led to the improvement of bimanual ability, unilateral dexterity, self-care function, and functional goals, and the improvements were mostly maintained during the follow-up period (duration not specified).

Novak et al. (42), based on three RCTs in hemiplegic children aged 2–10 years, reported that CIMT (total duration of intervention 90 hours) was equally effective for improving bimanual performance and unimanual capacity as dose-matched occupational therapy or HABIT (bimanual training). Then, recommended using both approaches and selecting one according to the family's preferences.

#### 3.2.3. Constraint-induced movement therapy

NICE CPG (12), as previously reported, recommended combining CIMT and bimanual therapy into an intensive program over a short time (for example, 4–8 weeks), to enhance manual skills.

The Australian CPG (13) reported that the modified model of CIMT (mCIMT) involving the use of slings, mitts, and splints for up to 2 h a day, but for a longer overall duration, was as effective as traditional CIMT (restraint applied for most of the waking day). Modified CIMT was recommended with an age-dependent model: shorter periods of daily practice at home and/or preschool over an 8–10-week period, under the age of 4 years; and intensive 2–3-week camps or group-based intervention, over 4 years of age. The CPG declared that higher intensity did not always bring better outcomes and CIMT did not result in age-dependent outcomes, although children with poorer hand function tended to make greater improvements.

Hoare et al. (48) conducted a SR with meta-analysis including 36 RCTs, in unilateral CP subjects with a mean age of 5.96 years (3 months−19.8 years). The most common constraint devices were a mitt/glove or a sling (11 studies each); the frequency was 2–7 days/week and the duration of intervention sessions was 0.5–8 h per day, for 1–10 weeks. The mixed outcome measures are as follows: AHA, QUEST, MUUL, BBT, and ABILHAND-Kids. CIMT appeared no more effective than another upper-limb therapy that was carried out intensively (most comparisons were with intensive bimanual therapist-led interventions). CIMT did not appear to impact body structure and function outcomes, such as grip strength, muscle stiffness, and spasticity. It had no consistent effect on quality of life and there was minimal research on participation outcomes. Two key ingredients across all models of CIMT were maintained: (1) restraint of the well-functioning upper limb (irrespective of device/type); and (2) intensive, structured training (irrespective of type). CIMT appeared to be a safe intervention for children with unilateral CP. The authors were not able to identify the characteristics of children who could be advised to participate in one or the other of CIMT or bimanual interventions. Therefore, they recommended to choose considering the developmental needs, child and family characteristics and preferences, therapist expertise, costs of implementing the intervention, funding and service delivery models, and resource availability.

Novak et al. (42) reported data from two SRs including hemiplegic children aged 3 months−19 years. The authors recommended CIMT to improve bimanual performance, unimanual capacity, activity, and participation in hemiplegic CP. CIMT conferred better activity and participation gains than no therapy, with large effect sizes, but it was equally effective to dose-matched occupational therapy.

Finally, also the SR by Das et al. (45), involving mostly hemiplegic patients, aged 0–18 years, confirmed the use of CIMT to improve upper-extremity functioning.

#### 3.2.4. Home programs

NICE CPG (12) recommended considering the following items when deciding who should deliver physical therapy:

- whether the child or young person and their parents or careers were able to deliver the specific therapy.
- what training the child or young person, or their parents or careers might need.
- the wishes of the child or young person and their parents or careers.

The Australian CPG (13) recommended home programs aimed at improving the performance of functional activities when based on the following five-step model:

- Establish collaborative relationships between parents and therapist.
- Set mutually agreed upon family and child goals.
- Select therapeutic activities that focus on achieving family and child goals, supported by the best available evidence.
- Support implementation of the home program through parent education, home visits, and program updates to sustain motivation.
- Evaluate outcomes.

The use of appropriate outcome measures for evaluation was recommended. The authors concluded that there was insufficient evidence to support the use of home programs aimed at improving participation.

Beckers et al. (49) reviewed 26 RCTs and four single-subject studies, involving uni/bilateral CP subjects, aged 4 months−19 years, at GMFCS levels I–V. The authors reported that no conclusions could be drawn due to the large variability in the study, patient and intervention characteristics, comparators, and outcome measures used in the included studies. Even within the same treatment approach, the frequency and duration of the interventions varied. Training intensity confirmed to be an important predictor of treatment success.

Novak et al. (42) based on two RCTs (GMFCS I–V, age 4–13 years) reported that home programs conferred improved function compared to no therapy and were an effective way to increase the dose of therapy.

#### 3.2.5. Action observation therapy

No recommendation was available on this topic in the included CPGs.

Abdelhaleem et al. (50) conducted a SR with meta-analysis, including 12 RCTs, with uni-bilateral CP subjects aged 5–15 years. No evidence of benefit had been found to draw a firm conclusion regarding the effectiveness of AOT, due to limitations in methodological quality and variations between studies.

The SR by Alamer et al. (51) included nine RCTs, with hemiplegic subjects aged 3–12 years, at GMFCS levels I–IV. The authors suggested that AOT is more effective than simple motor training, to improve physical function and structure, activities, and participation. However, the authors recalled particular attention when applying AOT for CP children with severe motor and cognitive impairment, and recommended further studies to determine the optimal frequency, intensity, and time of AOT.

The SR by Novak et al. (42) included only two RCTs, with ambulatory spastic unilateral CP subjects aged 5–15 years. The duration of intervention was 1 h a day for 15 days−3 weeks. Upper limb action observation training conferred better bimanual performance compared to watching videos but with a small effect size.

#### 3.2.6. Hand and arm bimanual intensive training including lower extremity

No recommendations were found on this topic. Only one SR by Novak et al. (42) was available on databases, including two RCTs, with uni-bilateral CP subjects aged 6–16 years, at GMFCS levels I–IV. The intervention lasted 90 h and was in a camp setting. The authors reported low evidence of improved motor function in both lower and upper limbs, compared to usual care.

#### 3.2.7. Adapted physical therapy and physical activity

An adapted physical therapy program was recommended by NICE CPG (12), following treatment with botulinum toxin type A, continuous pump-administered intrathecal baclofen, orthopedic surgery, or selective dorsal rhizotomy. Furthermore, the authors recommended considering muscle-strengthening therapy where the assessment indicated that muscle weakness was contributing to the loss of function or postural difficulties, using progressive repetitive exercises performed against resistance.

The Australian CPG (13) promoted gait training, defining it as the process of first learning or re-learning how to walk, after an intervention such as orthopedic surgery. It could be achieved in several ways, but repetition of the actual motions/gait pattern performed during walking was reported as the most important factor. Depending on the severity of the person's impairment, one or more physiotherapists, parallel bars, and high- or low-support assistive mobility devices might be involved to facilitate the gait pattern. Furthermore, the authors stated that strength-training in the lower limbs could be an accepted intervention for children with cerebral palsy, despite the lack of evidence regarding the effects on activity and participation. No adverse increase in spasticity was reported. The authors suggested setting the strengthening programs relying on the guidelines published by The American Academy of Pediatrics and the National Strength and Conditioning Association (NSCA), and complying with the following requirements:

- To perform a small number of repetitions until fatigue.
- To allow sufficient rest between exercises for recovery.
- Not to be performed frequently or for long durations.
- To increase the resistance as the ability to generate force increases.

The strength training should be combined with other activity-based programs such as treadmill training or cycling, involving other aspects of function such as endurance and coordination. Finally, the Australian CPG (13) recommended fitness training, defined as “planned structured activities involving repeated movement of skeletal muscles that result in energy expenditure to improve or maintain levels of physical fitness”. Aerobic fitness training provided short-term benefits for clients with sufficient motor skills to be able to undertake training, which was not maintained when training stopped. The frequency and intensity of interventions varied across the literature and generally focused on structured moderate to vigorous exercise. Attention was shifted recognizing the importance of reducing sedentary behavior and encouraging light-intensity activities throughout the day. It was recommended that fitness training to improve aerobic fitness, muscle strength, and the general health of children with cerebral palsy should be integrated into the child's daily life on an ongoing basis.

The SR by Corsi et al. (52) included 13 RCTs about gait or strength training, with uni-bilateral CP subjects, aged 7–18 years, at GMFCS levels I–III. Vibratory platform, gait training, electrical stimulation, and transcranial stimulation were effective to improve spatiotemporal gait parameters, especially velocity. Conversely, isolated strength training was not effective to improve gait parameters in CP.

Liang et al. (53) conducted a SR and meta-analysis, including 27 RCTs, in uni-bilateral CP, at GMFCS levels I–III, with a mean age of 1.8–16 years. Exercise interventions (resistance or aerobic or mixed training) showed beneficial effects on gait speed and muscle strength, but no significant effect on gross motor function in children with CP.

Merino-Andres et al. (54) conducted a SR with meta-analysis, including 27 RCTs [most studies had been analyzed also by Ryan et al. (60)]. Uni-bilateral CP subjects involved were aged 3–22 years, at GMFCS levels I–IV. The authors reported improvements after strength training programs, compared to other physical therapy techniques or untreated control groups, for muscle strength at the knee flexors, at the knee extensors, at the plantar flexors, maximum resistance, balance, gait speed, GMFM (global, D and E dimension), and spasticity.

Bania et al. (55) conducted a SR and meta-analysis including nine RCTs, with CP subjects aged 2–18 years (most were over 6 years), at GMFCS levels I–III. Activity training on the ground (whole-body self-initiated activities such as sitting, turning, sit-to-stand, walking, stepping, stair climbing, or other similar activities people use to transfer independently or with handheld support at home or outdoor settings) compared to no treatment or usual treatment (Neurodevelopmental treatment—NDT—or strengthening) showed no statistically significant difference.

The SR and meta-analysis by Armstrong et al. (56) about cycling, analyzed five RCTs, one quasi-RCT, one comparison trial, one pre-post study with a control group, and one single-group study with a control period. Uni-bilateral CP subjects at GMFCS levels I–V, mean age 10.4 years (SD 2.3), were involved. The authors concluded that cycling could improve aerobic fitness, muscle strength, balance, and gross motor function in children with CP; however, evidence was limited by small sample sizes, inconsistent outcome measures, and a lack of follow-up testing.

The SR by Lopez et al. (57) enquired about dance and Rhythmic Auditory Stimulation (RAS), selecting one case study, 10 clinical trials (three RCTs), and three pilot studies, involving either children or adults. The authors reported a positive impact on body functions, emotional expression, social participation, and attitudinal change as areas for consideration in future research. Nonetheless, the level of evidence was very low.

Das et al. (45) analyzed 34 SRs, involving mostly hemiplegic subjects aged 0–18 years. Intensive activity-based, goal-directed interventions resulted to be more effective than passive non-functional approaches, such as manual stretching, whose ability to increase range of motion and reduce spasticity was limited.

Collado-Garrido et al. (58) conducted a SR including 12 RCTs and three non-RCTs, with uni-bilateral CP subjects aged 4–18 years, at GMFCS levels I–V. The authors reported a statistically significant positive effect on muscle strength and motor function following resistance therapy, though they also declared limitations due to publication bias.

The SR (17 RCTs and 17 non-RCTs) by Clutterbuck et al. (59) enquired about several active exercise interventions (gross motor activity training alone or with progressive resistance exercise plus additional physiotherapy, physical fitness training, modified sport, and non-immersive virtual reality), in subjects affected by CP mixed types, aged 3–18 years, at GMFCS I–IV (mostly I–III). The authors reported an improvement in gross motor function of ambulant/semi-ambulant children, in particular, following gross motor activity training. They also indicated that practice variability is essential to improve gross motor function.

The SR by Novak et al. (42) enquired about several approaches: mobility training, strength training, aerobic exercise, physical activity, and modified sports.

The mobility training studies (six SRs) examined an eclectic group of interventions including Nintendo, wall climbing, sit-to-stand, circuit training of functional tasks, and overground or treadmill walking. Subjects aged 3–21 years, at GMFCS levels I–IV, were involved. The authors reported low- to moderate-level evidence of improving gait speed and gross motor function.

The strength training studies (four SRs), involved mixed CP subjects, aged 3.4–20 years, GMFCS levels I–III, and confirmed improved muscle strength and gait.

Moderate-based evidence [including Ryan et al. (60)] supported aerobic exercise (including cycling and treadmill) in children of GMFCS I–II who could move fast enough to train in aerobic fitness, to improve gross motor function in the short and intermediate term, without affecting gait speed.

Concerning physical activity, the authors reported low-level evidence (four SRs) and conflicting results on improving gross motor function, gait, and fitness, in subjects aged < 25 years, affected by CP mixed types, GMFCS levels I–V.

Very low-level evidence (observational studies in CP mixed types, GMFCS I–III, 4–16 years) supported modified sports, to improve gross motor skills, gait speed, and aerobic fitness.

The SR with the meta-analysis by Ryan et al. (60) was older (2017) than previous studies, but of higher quality and was included in the SRs by Novak et al. (42) and Merino-Andres et al. (54). It analyzed 29 RCTs (eight compared aerobic exercise to usual care, 15 compared resistance training to either usual care or no treatment, four compared mixed training to usual care or no treatment, and two compared aerobic exercise to resistance training) evaluated as low- to very low-level evidence. Samples were CP mixed types, GMFCS I–V, of ages < 19 years. Aerobic exercise improved motor function (activity level) but did not improve gait speed, walking endurance, participation, or aerobic fitness among children with CP in the short or intermediate term. There was no research regarding the effect of aerobic exercise on participation or quality of life. Resistance training did not improve motor function, gait speed, or participation in the short or intermediate term, or quality of life in the short term, in children and adolescents with CP but improved muscle strength. Mixed training did not improve motor function or gait speed but appeared to improve participation in children and adolescents with CP in the short term. No difference was evidenced between aerobic and resistance training on motor function, but a difference in muscle strength in the short term. Although the evidence suggested that exercise might be safe for people with CP, only 16 trials (55%) included information on adverse events; these trials reported no serious adverse events.

Elnahhas et al. (61) conducted a SR (seven RCTs) on backward gait training, involving uni-bilateral spastic CP, GMFCS levels I–III, of ages 5–14 years. The authors reported moderate evidence that backward gait training improved mobility (gait) and some evidence that it improved balance and gross motor function.

The SR and meta-analysis by Araujo et al. (62) involved uni-bilateral spastic CP subjects aged 5–15 years, at GMFCS levels I–II (incomplete data). Very low-quality evidence suggested that balance-training interventions (i.e., activities that caused unpredicted perturbations, such as unstable or mobile surfaces, in multiple training settings) combined with other interventions enhanced the effect of the other intervention alone on postural control in the short term.

Inamdar et al. (43) conducted a SR with meta-analysis (12 RCTs), enquiring about several approaches to improve sitting in uni-bilateral CP children, aged 18 months–puberty, at GMFCS I–V. The authors suggested that task-specific, intensive, and child-initiated intervention components might improve sitting in young infants at risk for CP, while components of impairment remediation combined with functional balance training should be explored to improve sitting in children diagnosed with CP.

Yardimci-Lokmanoglu et al. (63) conducted a SR including three small RCTs, with spastic CP subjects aged 5–15 years, at GMFCS levels I–III. Different approaches to proprioception (i.e., whole body vibration or integrated intensive proprioceptive and visuomotor training) combined with conventional physical therapy (CPT), showed no superiority in motor performance, compared to CPT alone.

#### 3.2.8. Treadmill and mechanically assisted walking

The Australian CPG (13) described treadmill training among recommended treatments, with or without partial body-weight support. The authors reported low-quality evidence to support treadmill training to improve weight-bearing and improve functional walking, although the practice of overground walking, rather than treadmill training might be more effective.

The SR and meta-analysis by Chiu et al. (64) analyzed 17 RCTs, in uni-bilateral CP subjects, aged 4–14 years, at GMFCS levels I–IV. The duration of the intervention was 4–12 weeks, the intensity of training was 15–40 min, and the frequency was 2–5 days/week. Compared with no walking, mechanically assisted walking training resulted in small improvements in walking speed (with or without body weight support) and gross motor function (with body weight support). Compared with the same dose of overground walking, mechanically assisted walking training with body weight support resulted in little to no difference in walking speed and gross motor function. Two studies found that mechanically assisted walking training without body weight support was probably more effective than the same dose of overground walking training for walking speed and gross motor function. Not many studies reported adverse events, although those that did report appeared to show no differences between groups. The results were largely not clinically significant, sample sizes were small, and the risk of bias and intensity of intervention varied across studies, making it hard to draw robust conclusions.

The SR with the meta-analysis by Han et al. (65) included eight RCTs, with uni-bilateral CP patients, GMFCS levels I–IV, with a mean age of 4.5–16 years. Findings suggested that treadmill training was effective for gait endurance, gait speed, and limb support time. No significant improvement was observed in cadence and step length.

Novak et al. (42) examined three SRs in uni-bilateral CP patients, aged 4–21 years at GMFCS levels I–IV. The authors reported that treadmill training, with or without body weight support, conferred improved walking speed, endurance, and gross motor function.

#### 3.2.9. Virtual reality for upper arm activities

No recommendation was available on this topic in included CPGs.

The SR with the meta-analysis by Johansen et al. (66) included eight RCTs, in CP subjects aged 5–20 years, at GMFCS levels I–V. The results highlighted the potential of video games (task-oriented, motivating, and intensive) as a supplementary method of training arm and hand functions for persons with CP. Nonetheless, they should be interpreted with caution due to the high risk of bias and low level of evidence.

Also, the SR by Plasschaert et al. (67) (two studies in bilateral CP) reported very low-level evidence for improvement in upper limb function.

Rathinam et al. (68) published a SR including six RCTs, in uni/bilateral CP subjects, aged 6–18 years, at GMFCS levels I–V. Four studies reported some improvement in hand function, but only one had a low risk of bias. The authors reported that the available evidence was inconsistent and that VR could not be reliably suggested to improve hand function until further studies had ascertained its therapeutic effect.

Conversely, Novak et al. (42) reported that VR conferred better arm function than NDT or usual care, with large effect sizes, based on a SR (19 RCTs) in CP subjects aged 4–12 years. The duration of the intervention was 20–90 min/day, 1–7 days/week, and over 4–20 weeks. The authors suggested the use of VR as a complement to conventional therapies and not as a substitute.

#### 3.2.10. Virtual reality for gross motor and balance activities

No recommendation was available on this topic in included CPGs.

Montoro-Cardenas et al. (69) enquired about the effectiveness of Nintendo WII Balance (NWT), for improving functional and dynamic balance, in spastic uni-bilateral CP children, at GMFCS levels I–IV. NWT was combined with CPT in 30-min sessions with interventions lasting longer than 3 weeks. Very low-quality evidence was found with a large effect of NWT compared with no intervention and moderate quality evidence for using NWT with CPT vs. CPT for improving dynamic balance.

The SR by Wu et al. (70) included 11 RCTs, in uni-bilateral CP subjects >6 years, at GMFCS I–IV (but data were incomplete). VR games played a positive role in the improvement of balance, but the evidence was limited by the methodological defects of included studies.

Ren et al. (71) analyzed seven RCTs, in uni-bilateral CP subjects >6 years, at GMFCS I–V. The authors reported preliminary evidence that VRGs improved the gross motor skills of children with CP. The single intervention time was 17–40 min and the intervention frequency was >5 times per week, over 12 weeks.

The SR by Pin et al. (72) included 21 studies (10 RCTs) in CP subjects at GMFCS levels I–II, with a mean age of over 4.8 years. ICP (interactive computer play) seemed to be more effective than conventional therapy in improving postural control and balance, with medium to large effect sizes.

Warnier et al. (73) conducted a SR with meta-analysis, including 26 studies (nine RCTs), in CP subjects mostly at GMFCS level I, aged 6–18 years. The meta-analysis confirmed the positive effect of VR, though results should be interpreted with caution due to differences in the interventions used, the lack of randomized controlled trials, and the relatively small groups.

In the SR (14 RCTs) by Ghai et al. (40), 88% of the studies reported significant enhancements in gait performance after training with VR. Meta-analyses revealed positive effects of virtual reality training on gait velocity (Hedge's g = 0.68), stride length (0.30), cadence (0.66), and gross motor function measure (0.44). Subgroup analysis reported a training duration of 20–30 min per session, ≤ 4 times per week across ≥8 weeks to allow maximum enhancements in gait velocity.

Novak et al. (42) presented results from one observational study, involving subjects aged 4–12 years, and compared VR + biofeedback vs. VR alone: the combination conferred better balance than VR alone. The authors also reviewed one RCT and five observational studies, involving subjects aged 5–18 years, at GMFCS levels I–III. Wii Fit appeared to confer improved balance.

Araujo et al. (62) analyzed just one RCT, in spastic hemiplegic CP, with a mean age of 9.6 years (SD 2.6), at GMFCS I–II. Wii therapy and NDT, compared to NDT, improved balance in terms of PBS in the short term (12 weeks).

#### 3.2.11. Hydrotherapy

The Australian CPG (13) claimed for further research on hydrotherapy, nonetheless, outlined some positive aspects of this approach: the warmth and buoyancy of the water might provide support and pain relief, by assisting relaxation and reducing spasms; walking might be possible without aides; and fitness and endurance might be more easily challenged in a controlled way. Hydrotherapy was also presented as an excellent recreational pursuit that could lead to improved swimming skills and respiratory function.

The SR by Roostaei et al. (39) included 11 studies (two RCTs), with uni-bilateral CP mixed types, GMFCS levels I–V, with ages 3–21 years. The treatment had a frequency of 2–3 days/week and a duration of 6–16 weeks. Evidence was limited. The aquatic exercise was feasible and adverse effects were minimal. However, the authors claimed the need for further research defining dosing parameters across age categories and GMFCS levels, the aquatic setting (type of pool and temperature of the water), and group or individualized treatment.

Novak et al. (42), based on low-quality evidence [including Roostaei et al. (39)], reported that aquatic-based exercises improved vitals and gross motor function.

#### 3.2.12. Non-invasive brain stimulation

The NIBS includes transcranial direct current stimulation (tDCS) and repetitive transcranial magnetic stimulation (rTMS).

Elbanna et al. (74) reported data from 14 RCTs comparing tDCS or rTMS with or without treadmill or VR vs. sham rTMS or placebo or NDT or treadmill training. A mixed population of CP, traumatic brain injury, or pediatric stroke ≤ 18 years, was considered. The authors concluded that rTMS improved upper limb function and tDCS improved balance and the majority of gait variables, but the level of evidence was low, and no long-term follow-up was provided. No adverse effects were described.

Novak et al. (42) presented results from four SRs, involving spastic or dystonic CP, aged 4–19 years. tDCS combined with treadmill or VR appeared to confer improved gait velocity, stride length, cadence, and balance compared to sham tDCS and rehabilitation. Adverse effects were rare, mild, and transient and included minor tingling, burning, itching, and skin redness.

Corsi et al. (52) reported data from three RCTs, involving uni-bilateral CP, GMFCS I-III, aged 7–18 years. tDCS combined with virtual reality or treadmill was effective to improve spatiotemporal gait parameters, especially velocity compared to sham stimulation. No follow-up was enquired.

#### 3.2.13. Neuromuscular electrical stimulation

The Australian CPG (13) reported emerging evidence to support the use of Functional Electric Stimulation (FES) for children with cerebral palsy in the lower limb and inconclusive evidence for its use in the upper limb.

The SR by Salazar et al. (75) examined six RCTs, in uni-bilateral CP, with a mean age of 1.04–8.6 years. Low-quality of evidence suggested that NMES might be used as an adjuvant therapy to improve gross motor function, particularly the sitting and standing dimensions of the GMFM scale. The evidence was limited due to the small number of studies included and the reduced sample size in each study. Further research with adequate methodological quality, ample sample size, and long-term follow-up was advised.

Corsi et al. (52) published a SR (five RCTs) enquiring about several treatments (vibratory platform, gait training, electrical stimulation, and transcranial stimulation), which all resulted to be effective to improve spatiotemporal gait parameters. The studies involved uni-bilateral CP, aged 7–18 years, at GMFCS levels I–III.

Conversely, the study by Das et al. (45) (34 SRs in uni-bilateral CP, 0–18 years) reported limited functional gain following NMES.

Controversial results about improving gait and low-level evidence about improving standing and sitting were reported by Novak et al. (42) (five SRs, in mixed CP types, GMFCS I–IV, and 1–19 years of age).

#### 3.2.14. Neurodevelopmental therapy

The Australian CPG (13) expressed a strong recommendation against NDT, because it considered the child as a relatively passive recipient of the treatment, and the approach was embedded into the context of normal developmental sequence.

Recent SRs (42, 45, 76, 87) all agreed reporting a lack of evidence to support the use of NDT in current practice.

#### 3.2.15. Hippotherapy

The Australian CPG (13) accounted for hippotherapy among adjunct interventions for children with CP, as it might have positive effects on balance and gross motor function, although evidence was limited.

The SR with the meta-analysis by Guindos-Sanchez et al. (77) (10 RCTs with mixed age subjects, GMFCS I-V) reported improvements in GMFM-66 total scores and GMFM-88 dimensions A, B, and E, balance recovery, and muscle spasticity reduction.

Novak et al. (42) (five SRs and three RCTs, in uni-bilateral CP subjects, aged 3–16 years, GMFCS levels I–V) attributed low level and conflicting evidence relative to gross motor function, but some positive effects on trunk position and arm function in GMFCS I–IV.

The SR by Araujo et al. (62) included just one low-level study dealing with hippotherapy (missing data about GMFCS, mean age 7 years, and uni-bilateral CP). A large additional effect on postural control was found when balance-training interventions (including hippotherapy) were combined with NDT at short-term (standardized mean difference of 1.3; 95% confidence interval 0.5, 2.0, *p* = 0.001). Nonetheless, the quality of the evidence was very low due to publication bias, imprecision, and inconsistency.

#### 3.2.16. Suit therapy

The Australian CPG (13) stated that there is conflicting and limited evidence on the benefits of suit therapy and claimed further research.

Novak et al. (42) (three SRs with CP mixed type, 3–17 years) reported that the suit might act on hip and shoulder stability and movement, given the suit was located over the hips and shoulders, whereas there was no effect on distal kinematics as the suit could not act on regions of the body not covered by the suit. Some children disliked wearing the suits and experienced adverse events including respiratory compromise, overheating, and peripheral cyanosis. The suits also impeded functions such as independent toileting and dressing.

The SR by Karadag-Saygi et al. (78) included 29 studies (nine RCTs) heterogenous in design, type of suit, size, study population, and outcomes measured. Some improvements were reported in proximal stability and gross motor function but with low evidence and several adverse effects.

#### 3.2.17. Taping

No recommendation was found in included CPGs on this topic.

The SR with the meta-analysis by Inamdar et al. (43) included 12 RCTs, in uni-bilateral subjects, aged 18 months–puberty, at GMFCS levels I–V. The authors reported that kinesio-taping might be an effective adjunct to conventional physical therapy in improving sitting ability in children with spastic bilateral CP.

Similarly, Novak et al. (42) (seven SRs, uni-bilateral CP, < 18 years, and GMFCS I–V) considered taping as an adjunct to therapy, not a stand-alone intervention, to improve gross motor and upper limb function. It was found to be most beneficial with GMFCS I–II, i.e., children with better selective motor control. Children had more active movement in the upper limbs when the tape was elasticized compared to rigid tape. A small number of children had a skin allergy to the tape, which was considered a contraindication.

#### 3.2.18. Orthoses

The Australian CPG (13) recommended the use of functional and positional orthoses, as common practice, even though the evidence was limited. Functional orthoses (e.g., ankle foot orthoses, wrist extension orthoses, neoprene wrist, and thumb orthoses) generally position joints in a biomechanically advantageous position to either enable or improve function. Positional orthoses (e.g., spinal braces, leg or elbow wraparounds, and hip abduction orthoses) aimed to maintain corrected anatomical alignment of the joint and maintain range of motion around that joint, to reduce the need for future orthopedic surgery and in some cases to maintain healthy skin integrity.

The SR by Betancourt et al. (79), including three RCTs and 14 prospective cohort studies (uni-bilateral CP, GMFCS I–IV, 3–18 years), reported that CP children using ankle-foot orthoses had improved stride length and dorsiflexion angle during gait.

#### 3.2.18. Serial casting

The Australian CPG (13) recommended casting (one cast or a series) to gain/restore muscle length and provide soft tissue elongation, in the short term, in the lower limb. While no evidence was reported to support upper limb casting. Casting was indicated when soft tissue contracture was interfering with function or causing potential biomechanical misalignment, not in the case of bony changes occurring at a joint. It was reported as particularly effective following botulinum toxin injections.

The SR by Milne et al. (80) analyzed 25 studies (mixed type, mostly had poor methodological quality) with a mixed population in two studies. Lower limb serial casting was found to be effective for improving ankle dorsiflexion (DF) passive range of motion (PROM) in the immediate to short term, decreasing hypertonicity measured by the Modified Ashworth Scale (MAS) in the short term. Serial casting with or without botulinum toxin did not significantly affect gross motor capacity measured by Gross Motor Function Measure. Serial casting with botulinum toxin achieved significantly more DF PROM than serial casting alone.

#### 3.2.19. Massage

The Australian CPG (13) accounted massage as one of the complementary and alternative medicines to relax a child after a bath, before sleeping, to relieve muscle pain, or to prepare for a therapy session. The authors reported the existence of a wide variety of massage techniques, from gentle effleurage to deep tissue massage or myofascial release, supported by little evidence of benefits in children with cerebral palsy.

Also, the SR by Guchan et al. (81) (11 studies including seven RCTs, in subjects aged 0–18 years, missing data relative to GMFCS level) suggested massage as an adjunct to traditional therapies to reduce muscle tone in spastic-type CP, but the evidence was at a very low level.

## 4. Discussion

### 4.1. Query 1

Relative to query 1, the selected CPGs (4, 11–13) presented recurrent shared issues, that may be synthetized as follows.

The management program needs to be aimed at specific goals, such as enhancing skill development, function, and ability to participate in everyday activities. It must be individually tailored, considering:

- needs and preferences of the child or young person and their parents or careers.
- the multidimensional profile of the child (holistic approach), including physical, mental, emotional, communicative, and relational features.
- age and developmentally appropriate activities as interventions and goals.
- functional ability scales (GMFCS, MACS, CFCS, VFCS, and EDACS) (82–86).
- Evidence-based interventions.
- implications (including emotional implications) for the individual child or young person and their parents or careers, including the time and effort involved and potential individual barriers.
- contextual barriers and possible difficulties in implementing the program.

In particular, the Australian CPG (13) recommended using functional motor ability classification scales (82–86) to guide assessment and intervention. High-quality observational studies (21–23) demonstrated the prognostic value of such classification scales and presented reference prognostic curves for gross motor and manual function. This frame helps to acknowledge the critical periods in which the intervention must focus on the limits of rehabilitation itself, to define the individualized realistic programs. Furthermore, the stabilizing of trajectories allows shifting from capacity-related intervention to goal-directed training and participation interventions, to promote new skills acquisition (23). All CPGs agree on the importance of providing baseline and regular assessment of the child or young person's functioning, using validated and specific tools, to ensure realistic goal setting, provide a baseline for therapy, and verify whether the goals are being achieved and/or the program remains appropriate to the child or young person's needs. A multiple-disciplinary (multidisciplinary, interdisciplinary, or transdisciplinary) team approach is advisable, including all child care professionals with expertise in CP management (pediatrician, neuropsychiatrist, physiatrist, physiotherapist, neuro-psychomotor therapist, occupational therapist, speech therapist, psychologist, orthopedic surgeon, nurse, orthotist, etc.), who may work within the same organization or as a network within the geographical area closest to the child, or at tertiary institutions or specialist services, together with educational professionals, to facilitate the provision of a holistic service (4). Finally, it is recommended to ensure the young person has access to adult services, both locally and regionally, that include healthcare professionals with an understanding of managing cerebral palsy (11, 13).

### 4.2. Query 2

Concerning query 2, the CPGs (12, 13) established the essential requirements merging all motor rehabilitation approaches in CP, which are synthetized as follows:

- individualized active use interventions.
- child-focused and age and developmentally appropriate goals to enhance motivation (i.e., playful activity or daily activity).
- the task should be analyzed considering the child's skills as well as environmental limitations.
- consider not only motor skills but the child's multidimensional profile.
- consider the impact of the intervention on the child and the family.
- intervention might be structured with adaptations of the task and/or of the context (objects and environment), based on the analysis of the child's skills, to support motivation and avoid frustration.
- intervention should involve the repetitive practice of a task or part of it, without incurring burnout in the child.
- intensive interventions over a brief period, in general, resulted to be more effective, but compliance of the child and the family is to be considered.

Therefore, passive interventions such as stretching (13, 45) or NDT (13, 42, 45, 76, 87) are considered ineffective in improving functions and activities. Nonetheless, stretching might have a role after botulinum injections are limited to improve PROM.

Previous issues on task-oriented, active-use, intensive treatment are mostly based on studies regarding manual performances (88), though CPGs (12, 13) and two selected SRs (43, 44) have extended them to gross motor function interventions. They all rely on the motor learning theory (89), which views movement emerging from the interaction of three systems: the person, the task, and the environment. Practice and experience alter the development of movement patterns through interaction with the environment and the demands of the task (90). Then, motor rehabilitation is not inhibition of primitive reflexes or normalization of movement but maximizing the efficiency of the damaged central nervous system (CNS), in response to the environment and demands of the task, leading to relatively permanent changes in the capability for movement and task performance (91).

The NICE CPG (12) generally talked about task-oriented active-use interventions aimed at individualized goals, while the Australian CPG (13) and the SR by Novak et al. (42) discussed child-oriented vs. context-oriented approaches and task-focused vs. goal-directed training, as alternatives, although they concluded they are all effective. It seems that these distinctions mostly respond to the need of categorizing the interventions for research studies and are based on underlying the predominant aspect. Nonetheless, in clinical practice, an overlap of these issues is often observed, and even in research studies, the distinction is not always so clear. From a more inclusive and general perspective, both issues may be considered components of the rehabilitation approach. In a child-oriented rehabilitation setting, the context (objects and environment) may be adapted to facilitate emerging skills and supporting motivation (92). Based on the performance and limitations of the child and young person, adaptations of the environment or of the objects may need to be transferred into the life contexts. Even the contraposition of task vs. goal-oriented approaches should be dampened, considering that any intervention to be effective must aim for goals that fit the subject in terms of being realistic and motivating (92–94). Then, also task-oriented interventions are expected to be set on individualized goals. Nonetheless, the results by Jackman et al. (41) and Eliasson et al. (23) suggested that younger children might be more responsive to task or part-task training, than older subjects, who still may improve on individual goals with goal-directed training. In this case, “goal-directed” is intended in a stricter view, as linked to individual activities, in a developmental stage in which improvement in the underlying functions is no more expected.

Another issue influencing the effectiveness is the intensity of treatment. Nonetheless, all CPGs (12, 13) are recommended considering the impact of treatment on CP child or young person and their family, and this may limit the frequency of the intervention. Jackman et al. (41) tried to define the minimal doses to reach success, in terms of the total amount of hours of treatment. The authors demonstrated that the interventions that set functional goals and involve the actual practice of those goals led to goal achievement at a lower dose than general upper limb motor training. Nonetheless, indicating the precise amount of training in terms of hours and risks to overcome the need for individualizing the intervention is based on the characteristics of the subjects, which is a priority. Furthermore, the evidence is limited because of heterogeneity and the absence or short follow-up of the included studies. The home programs (12, 13, 42, 49) may be considered to increase the dose of therapy, depending on family and child compliance. In this case, the requirements of the Australian CPG model (13) appear realistic and shareable:

- Establish collaborative relationships between parents and therapist.
- Set mutually agreed upon family and child goals.
- Select therapeutic activities that focus on achieving family and child goals that are supported by the best available evidence.
- Support implementation of the home program through parent education, home visits, and program updates to sustain motivation.
- Evaluate outcomes.

Beyond setting the general characteristics required by any motor rehabilitation approach, CPGs, in particular the Australian CPG, and the included SRs, reported a list of interventions that resulted effective in improving function and activities in children and young persons with CP. The individual interventions will be analyzed, distinguishing them as focusing on manual vs. gross motor performance.

#### 4.2.1. Manual function and activities

Two interventions were demonstrated to be effective for children with unilateral cerebral palsy, based on high-level evidence: bimanual therapy and constraint-induced movement therapy. Both provide time-limited, goal-directed, skills-based, intensive blocks of self-initiated movement practice based on motor learning theory (91). The evidence (12, 13, 42, 48) concludes that both can be used because they are equally effective at the same dose, and the choice must rely on the preferences of the family, the therapist's expertise, funding and service delivery models, and resource availability. Nonetheless, they are not the same.

Bimanual therapy is a process of learning bimanual hand skills through the repetitive use of carefully chosen, goal-related, two-handed activities that provoke specific bimanual actions and behaviors (91). It targets explicit learning or procedural knowledge through a mediated learning experience (95). The bimanual performance involves perceptual and cognitive processes underlying the movement response, based on the interaction among the child, the object, and the task. According to the action–perception theory, in a reciprocal and dynamic relationship, perception guides action, and action in turn allows for a more precise perception of future actions (88, 96).

CIMT involves placing a restraint on a child's less impaired upper limb to facilitate spontaneous and repetitive use of the impaired limb in a range of unimanual activities, specifically targeted to the child's individual ability and developmental level. Improvements are achieved by implicit learning (91), which is the ability to acquire a new skill without a corresponding increase in knowledge about the skill (97). It generally requires minimal attention and is not dependent on age and IQ (97). Typically, the type of tasks practiced in a CIMT program is discrete, while more complex tasks most often require two hands to perform. Furthermore, in the absence of a constraint device, these unimanual tasks would typically be performed using the dominant hand as it would be more effective with minimal effort (98). Then, CIMT does not allow practice and learning of how to use the more impaired hand for assisting hand actions, in complex bimanual activities. CIMT is effective for the development of unimanual actions brought about by implicit learning; however, it is not possible to target the cognitive and perceptual skills or explicit learning required for using two hands together to complete a task (91). As Hoare et al. (91) suggested, CIMT and bimanual should be viewed as complementary. CIMT could be used to target unimanual actions. Once these actions are established, bimanual therapy could be used for children to learn how to use these actions for bimanual skill development and learning how to perform daily activities with two hands (91).

The Australian CPG (13) reported that children with poorer function do tend to make greater improvement following CIMT. Nonetheless, possible frustration due to difficulties in performing functional tasks might affect the compliance of these subjects, and compliance is one of the basic requirements to be considered.

The evidence supporting the other approaches addressing manual performance is still limited.

The rationale for AOT is strong (99, 100), though results of available SRs are inconclusive (42, 50, 51) and future research is needed to verify the optimal frequency and intensity of AOT programs and characteristics of children that better fit the AOT approach, with particular attention to the severity of motor impairment and cognitive status as possible limitations.

VR as videogames (42, 66, 68) involving the upper limb might sustain engagement based on playful activities and releasing feedback to the subject's activation. Furthermore, it gives the possibility of controlling, reproducing, and measuring aspects of the activity enhancing its therapeutic potential. Some devices used in the studies are commonly recoverable and low-cost. Nonetheless, advances are required to define the type and parameters of the activity, and the evidence remains at a low level.

Based on the emerging literature, rTMS combined with active approaches might have a role in improving upper limb function (42, 52, 74), though further research is needed.

Inconclusive evidence was reported about the use of NMES to improve upper limb functions (13, 42, 45, 52, 75).

Orthoses (either functional or positional) (13, 79) and taping (42, 43) are extensively used by professionals to improve manual function and activities and prevent secondary deformities, even though the evidence supporting them is at a low level.

#### 4.2.2. Gross motor function and activities

All approaches addressing gross motor function and activities are supported by an overall low level of evidence.

Nonetheless, the CPGs recommend an adapted physical therapy program to acquire gross motor skills (i.e., learn for the first time) or recover them after an intervention (i.e., surgery or spasticity or dystonia treatment). Several approaches are described in the SRs to improve balance (62), sitting (43), mobility, and gait, based on low- to very low-level evidence: gross motor activity training (59), mobility training (42), balance training (62), sit-to-stand or other activity training on the ground (55), and gait training (52, 59, 61). NDT is excluded (13, 42, 45, 76). Devices, taping, and the aid of the therapist may be used to facilitate the activities. In general, it may be assumed that an adapted physical therapy program should include self-initiated task-specific activities, complying with the essential requirements previously established. It is worth recalling that this program should also comply with the GMFCS trajectories (21) and the individual developmental stage, in terms of realistic goals and appropriate activities. Further research is needed to better define the characteristics of such adapted physical training, which is anyway reported as advisable, to facilitate learning or re-learning gross motor skills after an intervention.

In the past, strength training was considered to be contraindicated in people with CP because it was thought to enhance muscle stiffness, then result in increased spasticity and decreased range of motion. The CPGs stated that resistance training is accepted, but the objective is just to improve muscle strength (53, 54, 58, 60), having no evidence of effectiveness on the activity and participation dimensions (60). It is not meant to be performed frequently, and for long durations, a small number of repetitions should be performed until fatigue and sufficient rest must be allowed between exercises for recovery. Strength training might be included either in adapted physical programs or in fitness training, though it is recommended to be combined with other activity-based programs, focusing on coordination and endurance.

Concerning gait, both overground and treadmill walking resulted effective (42, 60, 64) at the same dosage, in improving spatial-temporal parameters of gait and gross motor skills connected to gait, in the short to intermediate term, compared to no treatment (60). The treadmill may not replace an adapted physical therapy intervention overground, which implies the possibility to introduce devices and contextual aids to facilitate a child while learning to walk or distractors and obstacles to climb over to enhance the child's skills. Nonetheless, contextual factors may limit the possibility of exercising overground walking, while a treadmill might be accessible, to ensure either intensive treatment after surgery or daily physical activity to address fitness. Finally, it is advisable to consider the resources and preferences of subjects, their families, and service providers in the choice. The evidence regarding devices ensuring weight relief is inconclusive. Further research is needed to define the effectiveness, indications, and parameters of more complex and performing devices that may provide mechanically assisted walking and weight relief.

As an innovative issue, the CPGs recommended implementing physical activity, in terms of fitness training integrated into the child's daily life, to counteract the decline in mobility which is observed among young adults with CP. Adapted sports (42, 59), but also strength training, aerobic training, and mixed type are described, limited to clients with sufficient motor skills to be able to undertake training. Aerobic training included overground or treadmill walking (13, 42, 60) and cycling (56). The most effective dose of exercise for people with CP is currently unknown. Short-term benefits are reported in gross motor function and activities following aerobic or mixed training, but they are not maintained when the training stops. Many children, adolescents, and adults with CP have low levels of health-related fitness (muscle strength and cardiorespiratory endurance) and reduced habitual physical activity participation, which is well-known to be detrimental to cardiometabolic health (101). As in the general population, CP subjects should reduce sedentary behavior and increase daily physical activity (101). Children and young people with CP might need adaptations and/or aids to facilitate their participation, but these activities are beyond rehabilitation and should be integrated into their daily lives (13).

Immersive VR and VR games (40, 42, 62, 69–73) integrated with a platform or treadmill may help engagement in gross motor exercises, with the advantage of measuring and reproducing the characteristics of the exercise. Nonetheless, the evidence supporting these approaches is limited and their feasibility is linked to service providers' resources, in terms of technologies.

HABIT-ILE (42) is one attempt to encode an intensive self-initiated, mobility training, to improve manual and gross motor function and activities, borrowing from the experience of HABIT. The evidence supporting this approach is still limited.

Hydrotherapy is reported by the Australian CPG (13) among complementary interventions. It may be considered in combination with other task-specific interventions, to recover gross motor function (39, 42), in particular, in the initial phases following orthopedic surgery. Furthermore, mobility training in the water, possibly warm for better comfort, may be an alternative approach to improve fitness. Possible limitations to consider might be open wounds, the child's reduced compliance, contextual barriers, and services' resources.

Low-level evidence suggests that hippotherapy may be considered as one possible complementary approach to improve trunk position and balance (13, 42, 62, 77).

Suit therapy is excluded because of possible adverse events (13, 42, 78), though future research is advisable to verify the positive role of suits as orthoses facilitating trunk control/alignment and upper limb function.

There is controversial evidence (13, 42, 45, 52, 75) to support the use of FES and NMES for children with cerebral palsy in the lower limb and poor data about adverse effects and compliance. Future research is advisable to assess the effectiveness of NMES in GMFCS I–III, in particular, following botulinum injections or orthopedic surgery.

There is emerging evidence (42, 52, 74) that tDCS combined with a motor learning rehabilitation intervention might be more effective in improving gait and balance, compared to the activity training alone. Nonetheless, further studies with longer follow-ups are required to draw conclusions, define parameters, and verify adverse effects.

Orthoses (either functional or positional) (13, 79) and taping (42, 43) are extensively used by professionals to improve gait and gross motor function and activities and prevent secondary deformities, even though the evidence supporting them is at a low level.

Ankle-foot casting (one or a series of casts depending on the desired outcome and the child's tolerance) may be used, following botulinum injection, to provide short-term stretch to improve dorsiflexion passive range of motion (13, 80). It is indicated that initial soft tissue contracture is interfering with function or causing potential biomechanical misalignment. Nonetheless, it is not indicated in the case of advanced contractures or when bony changes are occurring at a joint.

## 5. Limitations

The present SR reports the general characteristics of the interventions and their effectiveness. More detailed indications are needed regarding which people are more likely to undergo certain treatments, based on individual characteristics, such as age, psychological and cognitive profile, and type of CP. Furthermore, the effectiveness of rehabilitation depends on the plasticity of the nervous system, which may be genetically determined. For example, genetic variation in the dopamine system (102) and polymorphisms of the brain-derived neurotrophic factor (BDNF) gene (103) may influence treatment outcomes in children with cerebral palsy. Nonetheless, based on the studies included in the present systematic review, the evidence appeared too limited to permit defining more specific indications. In the future, a specific search and further research studies would be advisable on these topics.

## 6. Conclusion

All motor rehabilitation approaches to minors affected by CP must share the following fundamental characteristics: evidence-based, engaging active-involvement of the subject, individualized, age and developmentally appropriate, goal-directed, and skills-based, intensive, and time-limited, suitable for the needs and preferences of the child or young person and their family, feasible considering the implications for themselves and possible contextual limitations.

The following approaches showed evidence of effectiveness in improving manual functions and activities: bimanual therapy and mCIMT with high-level evidence; AOT, VR, and taping with low-level evidence.

All approaches addressing gross motor function and activities are supported by low-level evidence, though an adapted physical therapy, respecting previous requirements, is advisable to acquire gross motor skills to recover them, after an intervention (i.e., surgery or spasticity or dystonia treatment). Among these, several mobility training approaches were reported: balance training, functional tasks on the ground, gait training (overground or with a treadmill), backward walking, and cycling. Resistance training may be combined with them, considering that it may impact muscle strength, rather than gross motor activities.

Finally, as for the general population, it is advisable to increase physical activity integrated into the child's daily life, to maintain or improve fitness, with a possible positive impact on gross motor activities. The benefits recede following the withdrawal of the training. Aerobic activities are included (e.g., overground or treadmill walking, cycling, and dancing) and may be combined with strength training. Nonetheless, this is limited to subjects with sufficient motor skills to be able to undertake training.

Low-level evidence suggests that VR, hippotherapy, and hydrotherapy may be considered as possible complementary approaches in combination with previous interventions.

Future research is needed to demonstrate the effectiveness of NMES and NIBS, as complementary approaches, and to define parameters and indications.

Upper and lower limb orthoses, taping, and ankle-foot casting (following botulinum injections) are supported by low-level evidence, though largely used by professionals.

## Data availability statement

The original contributions presented in the study are included in the article/Supplementary material, further inquiries can be directed to the corresponding author.

## Author contributions

SF, EP, and SSa: conception and writing—original draft. AF: supervision. SSg and SSa: selection of studies. SF, SSg, SP, and SSa: quality assessment of studies. All authors: acquisition, analysis, and interpretation of the data. All authors have read and approved the final version of the manuscript.

## References

[1] Bax MC Flodmark O Tydeman C. Definition and classification of cerebral palsy. From syndrome toward disease. Dev Med Child Neurol Suppl. (2007) 109:39–41. 10.1111/j.1469-8749.2007.tb12627.x 17370481

[2] Surveillance of Cerebral Palsy in Europe. Surveillance of cerebral palsy in Europe: a collaboration of cerebral palsy surveys and registers. Surveillance of Cerebral Palsy in Europe (SCPE). Dev Med Child Neurol. (2000) 42:816–24. 10.1017/S0012162200001511 11132255

[3] Ferrari A Cioni G. Società Italiana di Medicina Fisica e Rehabilitativa-Società Italiana di Neuropsichiatria dell'Infanzia e dell'Adolescenza (SIMFER-SINPIA) Intersociety Commission. Guidelines for rehabilitation of children with cerebral palsy. Eura Medicophys. (2005) 41:243–60. 16249783

[4] Castelli E Fazzi E. SIMFER-SINPIA intersociety commission. Recommendations for the rehabilitation of children with cerebral palsy. Eur J Phys Rehabil Med. (2016) 52:691–703. 26629842

[5] Brouwers MC Spithoff K Kerkvliet K Alonso-Coello P Burgers J Cluzeau F . Development and validation of a tool to assess the quality of clinical practice guideline recommendations. JAMA Netw Open. (2020) 3: e205535. 10.1001/jamanetworkopen.2020.5535 32459354 PMC7254179

[6] Johnston A Kelly SE Hsieh SC Skidmore B Wells GA. Systematic reviews of clinical practice guidelines: a methodological guide. J Clin Epidemiol. (2019) 108:64–76. 10.1016/j.jclinepi.2018.11.030 30529647

[7] Shea BJ Reeves BC Wells G Thuku M Hamel C Moran J et al. AMSTAR 2: a critical appraisal tool for systematic reviews that include randomised or non-randomised studies of healthcare interventions, or both. BMJ. (2017) 358:j4008. 10.1136/bmj.j4008 28935701 PMC5833365

[8] Guyatt G Oxman AD Akl EA Kunz R Vist G Brozek J et al. GRADE guidelines: 1. Introduction-GRADE evidence profiles and summary of findings tables. J Clin Epidemiol. (2011) 64:383–94. 10.1016/j.jclinepi.2010.04.026 21195583

[9] Balshem H Helfand M Schünemann HJ Oxman AD Kunz R Brozek J . guidelines: 3. Rating the quality of evidence. J Clin Epidemiol. (2011) 64:401–6. 10.1016/j.jclinepi.2010.07.015 21208779

[10] Munn Z Barker TH Moola S Tufanaru C Stern C McArthur A et al. Methodological quality of case series studies: an introduction to the JBI critical appraisal tool. JBI Evid Synth. (2020) 18:2127–33. 10.11124/JBISRIR-D-19-00099 33038125

[11] NICE Guidelines: Managing cerebral palsy in under 25s. (2021). Available online at: http://pathways.nice.org.uk/pathways/cerebral-palsy (accessed August 6, 2021).

[12] NICE guidelines: Spasticity in under 19s: management. (2012-2016). Available online at: https://www.nice.org.uk/guidance/cg145 (accessed August 6, 2021).

[13] Management of Cerebral Palsy In Children: A Guide For Allied Health Professionals. (2018). Available online at: http://www.health.nsw.gov.au/kidsfamilies/ (accessed August 6, 2021).

[14] Rééducation et réadaptation de la fonction motrice des personnes porteuses de paralysie cérébrale. HAS: Haute Autorité de santé (Francia). (2020). Available online at: https://www.has-sante.fr/portail/ (accessed August 6, 2021).

[15] Anaby D Korner-Bitensky N Steven E Tremblay S Snider L Avery L et al. Current rehabilitation practices for children with cerebral palsy: focus and gaps. Phys Occup Ther Pediatr. (2017) 37:1–15. 10.3109/01942638.2015.1126880 26865220

[16] Graham D Paget SP Wimalasundera N. Current thinking in the health care management of children with cerebral palsy. Med J Aust. (2019) 210:129–35. 10.5694/mja2.12106 30739332

[17] Mäenpää H Autti-Rämö I Varho T Forsten W Haataja L. Multiprofessional evaluation in clinical practice: establishing a core set of outcome measures for children with cerebral palsy. Dev Med Child Neurol. (2017) 59:322–8. 10.1111/dmcn.13289 27714777

[18] Taczała J Wolińska O Becher J Majcher P. An interdisciplinary model of treatment of children with cerebral palsy in Poland. Recommendations of the paediatric rehabilitation section of the polish rehabilitation society. Ortop Traumatol Rehabil. (2020) 22:51–9. 10.5604/01.3001.0014.0641 32242520

[19] Torjesen I NICE. publishes guideline on diagnosing and managing cerebral palsy in young people. BMJ. (2017) 356:j462. 10.1136/bmj.j462 28130209

[20] Shaunak M Kelly VB. Cerebral palsy in under 25 s: assessment and management (NICE Guideline NG62). Arch Dis Child Educ Pract Ed. (2018) 103:189–93. 10.1136/archdischild-2017-312970 29056589

[21] Rosenbaum PL Walter SD Hanna SE Palisano RJ Russell DJ Raina P . Prognosis for gross motor function in cerebral palsy: creation of motor development curves. JAMA. (2002) 288:1357–63. 10.1001/jama.288.11.1357 12234229

[22] Klevberg GL Elvrum AG Zucknick M Elkjaer S Østensjø S Krumlinde-Sundholm L . Development of bimanual performance in young children with cerebral palsy. Dev Med Child Neurol. (2018) 60:490–7. 10.1111/dmcn.13680 29392717

[23] Eliasson AC Nordstrand L Backheden M Holmefur M. Longitudinal development of hand use in children with unilateral spastic cerebral palsy from 18 months to 18 years. Dev Med Child Neurol. (2022) 65:376–84. 10.1111/dmcn.15370 35899928 PMC10087588

[24] Hill M Healy A Chockalingam N. Effectiveness of therapeutic footwear for children: A systematic review. J Foot Ankle Res. (2020) 13:23. 10.1186/s13047-020-00390-3 32404124 PMC7222438

[25] Rozin Kleiner AF Bellomo A Pagnussat AS de Athayde Costa E Silva A Sforza C . Wearable sensors, cerebral palsy and gait assessment in everyday environments: is it a reality? A systematic review. Funct Neurol. (2019) 34:85–91. 31556388

[26] Novak I Honan I. Effectiveness of paediatric occupational therapy for children with disabilities: a systematic review. Aust Occup Ther J. (2019) 66:258–73. 10.1111/1440-1630.12573 30968419 PMC6850210

[27] Roostaei M Raji P Morone G Razi B Khademi-Kalantari K. The effect of dual-task conditions on gait and balance performance in children with cerebral palsy: a systematic review and meta-analysis of observational studies. J Bodyw Mov Ther. (2021) 26:448–62. 10.1016/j.jbmt.2020.12.011 33992282

[28] Saleem GT Crasta JE Slomine BS Cantarero GL Suskauer SJ. Transcranial direct current stimulation in pediatric motor disorders: a systematic review and meta-analysis. Arch Phys Med Rehabil. (2019) 100:724–38. 10.1016/j.apmr.2018.10.011 30414398 PMC7927962

[29] Valentin-Gudiol M Bagur-Calafat C Girabent-Farrés M Hadders-Algra M Mattern-Baxter K Angulo-Barroso R. Treadmill interventions with partial body weight support in children under six years of age at risk of neuromotor delay: a report of a Cochrane systematic review and meta-analysis. Eur J Phys Rehabil Med. (2013) 49:67–91. 23575201

[30] Chakraborty S Nandy A Kesar TM. Gait deficits and dynamic stability in children and adolescents with cerebral palsy: a systematic review and meta-analysis. Clin Biomech (Bristol, Avon). (2020) 71:11–23. 10.1016/j.clinbiomech.2019.09.005 31677546

[31] Chiu HC Ada L Chen C. Changes in walking performance between childhood and adulthood in cerebral palsy: a systematic review. Dev Neurorehabil. (2020) 23:343–8. 10.1080/17518423.2019.1648579 31366265

[32] Clutterbuck GL Auld ML Johnston LM. High-level motor skills assessment for ambulant children with cerebral palsy: a systematic review and decision tree. Dev Med Child Neurol. (2020) 62:693–9. 10.1111/dmcn.14524 32237147

[33] Huang C Chen Y Chen G Xie Y Mo J Li K et al. Efficacy and safety of core stability training on gait of children with cerebral palsy: a protocol for a systematic review and meta-analysis. Medicine (Baltimore). (2020) 99:e18609. 10.1097/MD.0000000000018609 31914039 PMC6959942

[34] Tanner K Schmidt E Martin K Bassi M. Interventions within the scope of occupational therapy practice to improve motor performance for children ages 0-5 years: a systematic review. Am J Occup Ther. (2020) 74:7402180060p1–7402180060p40. 10.5014/ajot.2020.039644 32204777

[35] Blumetti FC Belloti JC Tamaoki MJ Pinto JA. Botulinum toxin type A in the treatment of lower limb spasticity in children with cerebral palsy. Cochrane Database Syst Rev. (2019) 10:CD001408. 10.1002/14651858.CD001408.pub2 31591703 PMC6779591

[36] Corrado B Di Luise C Servodio Iammarrone C. Management of muscle spasticity in children with cerebral palsy by means of extracorporeal shockwave therapy: a systematic review of the literature. Dev Neurorehabil. (2021) 24:1–7. 10.1080/17518423.2019.1683908 31674272

[37] Kim HJ Park JW Nam K. Effect of extracorporeal shockwave therapy on muscle spasticity in patients with cerebral palsy: meta-analysis and systematic review. Eur J Phys Rehabil Med. (2019) 55:761–71. 10.23736/S1973-9087.19.05888-X 31615195

[38] Yana M Tutuola F Westwater-Wood S Kavlak E. The efficacy of botulinum toxin A lower limb injections in addition to physiotherapy approaches in children with cerebral palsy: a systematic review. NeuroRehabilitation. (2019) 44:175–89. 10.3233/NRE-182581 30856126

[39] Roostaei M Baharlouei H Azadi H Fragala-Pinkham MA. Effects of aquatic intervention on gross motor skills in children with cerebral palsy: a systematic review. Phys Occup Ther Pediatr. (2017) 37:496–515. 10.1080/01942638.2016.1247938 27967298

[40] Ghai S Ghai I. Virtual reality enhances gait in cerebral palsy: a training dose-response meta-analysis. Front Neurol. (2019) 10:236. 10.3389/fneur.2019.00236 30984095 PMC6448032

[41] Jackman M Lannin N Galea C Sakzewski L Miller L Novak I. What is the threshold dose of upper limb training for children with cerebral palsy to improve function? A systematic review. Aust Occup Ther J. (2020) 67:269–80. 10.1111/1440-1630.12666 32342517

[42] Novak I Morgan C Fahey M Finch M Galea C Hines A . State of the evidence traffic lights 2019: systematic review of interventions for preventing and treating children with cerebral palsy. Curr Neurol Neurosci Rep. (2020) 20:3. 10.1007/s11910-020-1022-z 32086598 PMC7035308

[43] Inamdar K Molinini RM Panibatla ST Chow JC Dusing SC. Physical therapy interventions to improve sitting ability in children with or at-risk for cerebral palsy: a systematic review and meta-analysis. Dev Med Child Neurol. (2021) 63:396–406. 10.1111/dmcn.14772 33319378

[44] Hsu CW Kang YN Tseng SH. Effects of therapeutic exercise intensity on cerebral palsy outcomes: a systematic review with meta-regression of randomized clinical trials. Front Neurol. (2019) 10:657. 10.3389/fneur.2019.00657 31293501 PMC6598595

[45] Das SP Ganesh GS. Evidence-based approach to physical therapy in cerebral palsy. Indian J Orthop. (2019) 53:20–34. 10.4103/ortho.IJOrtho_241_17 30905979 PMC6394183

[46] Alahmari K Tedla JS Sangadala DR Mukherjee D Reddy RS Bairapareddy KC . Effectiveness of hand-arm bimanual intensive therapy on hand function among children with unilateral spastic cerebral palsy: a meta-analysis. Eur Neurol. (2020) 83:131–7. 10.1159/000507325 32348996

[47] Ouyang RG Yang CN Qu YL Koduri MP Chien CW. Effectiveness of hand-arm bimanual intensive training on upper extremity function in children with cerebral palsy: A systematic review. Eur J Paediatr Neurol. (2020) 25:17–28. 10.1016/j.ejpn.2019.12.017 31902688

[48] Hoare BJ Wallen MA Thorley MN Jackman ML Carey LM Imms C. Constraint-induced movement therapy in children with unilateral cerebral palsy. Cochrane Database Syst Rev. (2019) 4:CD004149. 10.1002/14651858.CD004149.pub3 30932166 PMC6442500

[49] Beckers LWME Geijen MME Kleijnen J Ramecker EA Schnacker M Smeets R . Feasibility and effectiveness of home-based therapy programs for children with cerebral palsy: a systematic review. BMJ Open. (2020) 10: e035454. 10.1136/bmjopen-2019-035454 33028544 PMC7539606

[50] Abdelhaleem N Taher S Mahmoud M Hendawy A Hamed M Mortada H . Effect of action observation therapy on motor function in children with cerebral palsy: a systematic review of randomized controlled trials with meta-analysis. Clin Rehabil. (2021) 35:51–63. 10.1177/0269215520954345 32907374

[51] Alamer A Melese H Adugna B. Effectiveness of Action Observation Training on Upper Limb Motor Function in Children with Hemiplegic Cerebral Palsy: A Systematic Review of Randomized Controlled Trials. Pediatric Health Med Ther. (2020) 11:335–46. 10.2147/PHMT.S266720 32982541 PMC7501989

[52] Corsi C Santos MM Moreira RFC Dos Santos AN de Campos AC Galli M . Effect of physical therapy interventions on spatiotemporal gait parameters in children with cerebral palsy: a systematic review. Disabil Rehabil. (2021) 43:1507–16. 10.1080/09638288.2019.1671500 31588810

[53] Liang X Tan Z Yun G Cao J Wang J Liu Q Chen T. Effectiveness of exercise interventions for children with cerebral palsy: a systematic review and meta-analysis of randomized controlled trials. J Rehabil Med. (2021) 53:jrm00176. 10.2340/16501977-2772 33225375 PMC8814858

[54] Merino-Andrés J García de. Mateos-López A, Damiano DL, Sánchez-Sierra A. Effect of muscle strength training in children and adolescents with spastic cerebral palsy: A systematic review and meta-analysis Clin Rehabil. (2022) 36:4–14. 10.1177/02692155211040199 34407619 PMC9639012

[55] Bania T Chiu HC Billis E. Activity training on the ground in children with cerebral palsy: Systematic review and meta-analysis. Physiother Theory Pract. (2019) 35:810–21. 10.1080/09593985.2018.1460647 29659303

[56] Armstrong EL Spencer S Kentish MJ Horan SA Carty CP Boyd RN. Efficacy of cycling interventions to improve function in children and adolescents with cerebral palsy: a systematic review and meta-analysis. Clin Rehabil. (2019) 33:1113–29. 10.1177/0269215519837582 30935240

[57] López-Ortiz C Gaebler-Spira DJ Mckeeman SN Mcnish RN Green D. Dance and rehabilitation in cerebral palsy: a systematic search and review. Dev Med Child Neurol. (2019) 61:393–8. 10.1111/dmcn.14064 30350851

[58] Collado-Garrido L Parás-Bravo P Calvo-Martín P Santibáñez-Margüello M. Impact of Resistance Therapy on Motor Function in Children with Cerebral Palsy: A Systematic Review and Meta-Analysis. Int J Environ Res Public Health. (2019) 16:4513. 10.3390/ijerph16224513 31731636 PMC6888121

[59] Clutterbuck G Auld M Johnston L. Active exercise interventions improve gross motor function of ambulant/semi-ambulant children with cerebral palsy: a systematic review. Disabil Rehabil. (2019) 41:1131–51. 10.1080/09638288.2017.1422035 29303007

[60] Ryan JM Cassidy EE Noorduyn SG O'Connell NE. Exercise interventions for cerebral palsy. Cochrane Database Syst Rev. (2017) 6:CD011660. 10.1002/14651858.CD011660.pub2 28602046 PMC6481791

[61] Elnahhas AM Elshennawy S Aly MG. Effects of backward gait training on balance, gross motor function, and gait in children with cerebral palsy: a systematic review. Clin Rehabil. (2019) 33:3–12. 10.1177/0269215518790053 30043634

[62] Araújo PA Starling JMP Oliveira VC Gontijo APB Mancini MC. Combining balance-training interventions with other active interventions may enhance effects on postural control in children and adolescents with cerebral palsy: a systematic review and meta-analysis. Braz J Phys Ther. (2020) 24:295–305. 10.1016/j.bjpt.2019.04.005 31076254 PMC7351984

[63] Yardimci-Lokmanoglu BN Bingöl H Mutlu A. The forgotten sixth sense in cerebral palsy: do we have enough evidence for proprioceptive treatment? Disabil Rehabil. (2020) 42:3581–90. 10.1080/09638288.2019.1608321 31056965

[64] Chiu HC Ada L Bania TA. Mechanically assisted walking training for walking, participation, and quality of life in children with cerebral palsy. Cochrane Database Syst Rev. (2020) 11:CD013114. 10.1002/14651858.CD013114.pub2 33202482 PMC8092676

[65] Han YG Yun CK. Effectiveness of treadmill training on gait function in children with cerebral palsy: meta-analysis. J Exerc Rehabil. (2020) 16:10–9. 10.12965/jer.1938748.374 32161730 PMC7056486

[66] Johansen T Strøm V Simic J Rike PO. Effectiveness of training with motion-controlled commercial video games on hand and arm function in young people with cerebral palsy: A systematic review and meta-analysis. J Rehabil Med. (2019) 52:jrm00012. 10.2340/16501977-2633 31794044

[67] Plasschaert VFP Vriezekolk JE Aarts PBM Geurts ACH Van den Ende CHM. Interventions to improve upper limb function for children with bilateral cerebral palsy: a systematic review. Dev Med Child Neurol. (2019) 61:899–907. 10.1111/dmcn.14141 30632139 PMC6850353

[68] Rathinam C Mohan V Peirson J Skinner J Nethaji KS Kuhn I. Effectiveness of virtual reality in the treatment of hand function in children with cerebral palsy: a systematic review. J Hand Ther. (2019) 32:426–434.e1. 10.1016/j.jht.2018.01.006 30017414

[69] Montoro-Cárdenas D Cortés-Pérez I Zagalaz-Anula N Osuna-Pérez MC Obrero-Gaitán E Lomas-Vega R. Nintendo Wii Balance Board therapy for postural control in children with cerebral palsy: a systematic review and meta-analysis. Dev Med Child Neurol. (2021) 63:1262–75. 10.1111/dmcn.14947 34105150

[70] Wu J Loprinzi PD Ren Z. The rehabilitative effects of virtual reality games on balance performance among children with cerebral palsy: a meta-analysis of randomized controlled trials. Int J Environ Res Public Health. (2019) 16:4161. 10.3390/ijerph16214161 31661938 PMC6861947

[71] Ren Z Wu J. The effect of virtual reality games on the gross motor skills of children with cerebral palsy: a meta-analysis of randomized controlled trials. Int J Environ Res Public Health. (2019) 16:3885. 10.3390/ijerph16203885 31614990 PMC6843701

[72] Pin TW. Effectiveness of interactive computer play on balance and postural control for children with cerebral palsy: a systematic review. Gait Posture. (2019) 73:126–39. 10.1016/j.gaitpost.2019.07.122 31323621

[73] Warnier N Lambregts S Port IV. Effect of virtual reality therapy on balance and walking in children with cerebral palsy: a systematic review. Dev Neurorehabil. (2020) 23:502–18. 10.1080/17518423.2019.1683907 31674852

[74] Elbanna ST Elshennawy S Ayad MN. noninvasive brain stimulation for rehabilitation of pediatric motor disorders following brain injury: systematic review of randomized controlled trials. Arch Phys Med Rehabil. (2019) 100:1945–63. 10.1016/j.apmr.2019.04.009 31078616

[75] Salazar AP Pagnussat AS Pereira GA Scopel G Lukrafka JL. Neuromuscular electrical stimulation to improve gross motor function in children with cerebral palsy: a meta-analysis. Braz J Phys Ther. (2019) 23:378–86. 10.1016/j.bjpt.2019.01.006 30712812 PMC6823719

[76] Zanon MA Pacheco RL Latorraca COC Martimbianco ALC Pachito DV Riera R. Neurodevelopmental treatment (Bobath) for children with cerebral palsy: a systematic review. J Child Neurol. (2019) 34:679–86. 10.1177/0883073819852237 31179823

[77] Guindos-Sanchez L Lucena-Anton D Moral-Munoz JA Salazar A Carmona-Barrientos I. The effectiveness of hippotherapy to recover gross motor function in children with cerebral palsy: a systematic review and meta-analysis. Children (Basel). (2020) 7:106. 10.3390/children7090106 32825159 PMC7552760

[78] Karadag-Saygi E Giray E. The clinical aspects and effectiveness of suit therapies for cerebral palsy: a systematic review. Turk J Phys Med Rehabil. (2019) 65:93–110. 10.5606/tftrd.2019.3431 31453550 PMC6648185

[79] Betancourt JP Eleeh P Stark S Jain NB. Impact of ankle-foot orthosis on gait efficiency in ambulatory children with cerebral palsy: a systematic review and meta-analysis. Am J Phys Med Rehabil. (2019) 98:759–70. 10.1097/PHM.0000000000001185 30920399

[80] Milne N Miao M Beattie E. The effects of serial casting on lower limb function for children with Cerebral Palsy: a systematic review with meta-analysis. BMC Pediatr. (2020) 20:324. 10.1186/s12887-020-02122-9 32615954 PMC7330971

[81] Güçhan Z Mutlu A. The effectiveness of taping on children with cerebral palsy: a systematic review. Dev Med Child Neurol. (2017) 59:26–30. 10.1111/dmcn.13213 27476831

[82] Palisano R Rosenbaum P Walter S Russell D Wood E Galuppi B. Development and reliability of a system to classify gross motor function in children with cerebral palsy. Dev Med Child Neurol. (1997) 39:214–23. 10.1111/j.1469-8749.1997.tb07414.x 9183258

[83] Eliasson A-C Krumlinde-Sundholm L Rösblad B Beckung E Arner M Ohrvall A-M . The manual ability classification system (MACS) for children with cerebral palsy: scale development and evidence of validity and reliability. Dev Med Child Neurol. (2006) 48:549–54. 10.1017/S0012162206001162 16780622

[84] Hidecker MJC Paneth N Rosenbaum PL Kent RD Lillie J Eulenberg JB . Developing and validating the communication function classification system for individuals with cerebral palsy. Dev Med Child Neurol. (2011) 53:704–10. 10.1111/j.1469-8749.2011.03996.x 21707596 PMC3130799

[85] Sellers D Mandy A Pennington L Hankins M Morris C. Development and reliability of a system to classify the eating and drinking ability of people with cerebral palsy. Dev Med Child Neurol. (2014) 56:245–51. 10.1111/dmcn.12352 24344767

[86] Baranello G Signorini S Tinelli F Guzzetta A Pagliano E Rossi A . VFCS study group. Visual Function Classification System for children with cerebral palsy: development and validation. Dev Med Child Neurol. (2020) 62:104–10. 10.1111/dmcn.14270 31180136

[87] Novak I McIntyre S Morgan C Campbell L Dark L Morton N . A systematic review of interventions for children with cerebral palsy: state of the evidence. Dev Med Child Neurol. (2013) 55:885–910. 10.1111/dmcn.12246 23962350

[88] Greaves S Imms C Krumlinde-Sundholm L Dodd K Eliasson AC. Bimanual behaviours in children aged 8-18 months: a literature review to select toys that elicit the use of two hands. Res Dev Disabil. (2012) 33:240–50. 10.1016/j.ridd.2011.09.012 22093670

[89] Bernstein NA. The co-ordination and regulation of movements. Science. (1968) 158:415–6.

[90] Zwicker JG Harris SR A. reflection on motor learning theory in pediatric occupational therapy practice. Can J Occup Ther. (2009) 76:29–37. 10.1177/000841740907600108 19341020

[91] Hoare B Greaves S. Unimanual vs. bimanual therapy in children with unilateral cerebral palsy: Same, same, but different. J Pediatr Rehabil Med. (2017) 10:47–59. 10.3233/PRM-170410 28339410

[92] Borelli G Neviani R Sghedoni A Conti MR Montanari L Ovi A. La fisioterapia nella paralisi cerebrale infantile. Milan: Springer Verlag Italia. (2013). 10.1007/978-88-470-5277-2

[93] Ferrari A. Proposte riabilitative nelle paralisi cerebrali infantile. In: Edizioni Del Cerro. (1997).

[94] Fedrizzi E. I diversi livelli dell'intervento globale nella riabilitazione del bambino con paralisi cerebrale infantile. In: Giornale Italiano di Medicina Riabilitativa. (1987).

[95] Feuerstein R Hoffman MB Rand Y Jensen MR Tzuriel D Hoffmann DB. Learning to learn: mediated learning experiences and instrumental enrichment. Special Services in the Schools. (2010) 3:49–82. 10.1300/J008v03n01_0534942939

[96] Ferrari A. From movement to action: a new framework for cerebral palsy. Eur J Phys Rehabil Med. (2019) 55:852–61. 10.23736/S1973-9087.19.05845-3 31556512

[97] Jongbloed-Pereboom M Janssen AJ Steenbergen B Nijhuis-van der Sanden MW. Motor learning and working memory in children born preterm: a systematic review. Neurosci Biobehav Rev. (2012) 36:1314–30. 10.1016/j.neubiorev.2012.02.005 22353425

[98] Krumlinde Sundholm L. On the other hand: About successful use of two hands together. Conference proceedings from the Third International Cerebral Palsy Conference. Dev Med Child Neur. (2009) 51:39.

[99] Iacoboni M Woods RP Brass M Bekkering H Mazziotta JC Rizzolatti G. Cortical mechanisms of human imitation. Science. (1999) 286:2526–8. 10.1126/science.286.5449.2526 10617472

[100] Fogassi L Ferrari PF Gesierich B Rozzi S Chersi F Rizzolatti G. Parietal lobe: from action organization to intention understanding. Science. (2005) 308:662–7. 10.1126/science.1106138 15860620

[101] Verschuren O Peterson MD Balemans AC Hurvitz EA. Exercise and physical activity recommendations for people with cerebral palsy. Dev Med Child Neurol. (2016) 58:798–808. 10.1111/dmcn.13053 26853808 PMC4942358

[102] Diaz Heijtz R Almeida R Eliasson AC Forssberg H. Genetic variation in the dopamine system influences intervention outcome in children with cerebral palsy. EBioMedicine. (2018) 28:162–7. 10.1016/j.ebiom.2017.12.028 29339100 PMC5835543

[103] Bagrowski B Czapracka M Kraśny J Prendecki M Dorszewska J Józwiak M. Assessment of the relationship between Val66Met BDNF polymorphism and the effectiveness of gait rehabilitation in children and adolescents with cerebral palsy. Acta Neurobiol Exp (Wars). (2022) 82:1–11. 10.55782/ane-2022-001 35451419

