# Supplementary material for: Evidence-based management and motor rehabilitation of cerebral palsy children and adolescents: a systematic review
Source: Front Neurol. 2023 May 25;14:1171224. doi: 10.3389/fneur.2023.1171224 (PMC10248244; doi:10.3389/fneur.2023.1171224)
Supplement: Supplementary file 1 [file Data_Sheet_1.docx]

**Supplementary Digital Material 1 (Supplementary Text File online content only).**

Search and selection procedures.

The scope of the systematic review was structured in research questions, according to the PICO (Patients, Intervention, Control, Outcome) framework. The following questions were considered:

1. Which are the general principles to provide a comprehensive management of CP subjects under age 18 years? (P: CP subjects under age 18 years; I: management; C: no treatment; O: any outcome)
2. Which are the most effective motor rehabilitation approaches to improve gross motor or upper limb performance, in CP subjects aged 2-18 years? (P: CP subjects aged 2-18 years; I: any motor rehabilitation interventions, excluded surgery or drugs; C: no treatment or any other rehabilitative intervention; O: all outcomes relative to gross motor or manual function and/or activities).

Query 2 was deliberately maintained inclusive, rather than providing separated queries for gross motor or manual functions and activities, because several studies involved both aspects as outcomes.

Available evidence on each question was systematically enquired.

Clinical practice guidelines (CPGs) were firstly searched, relative to CP management and rehabilitation. In case of missing or incomplete evidence, to answer the identified queries, the search was extended to systematic reviews (SRs). Screening and selection was independently executed by two evaluators (SG and SS), by firstly assessing titles and abstracts, secondarily full texts. Any discrepancies among the evaluators was resolved through discussion.

Guidelines were firstly searched on the websites (6 August 2021) of the following organizations:

- NICE: National Istitute for Healt and Care Excellence (Regno Unito) https://www.nice.org.uk/

- HAS: Haute Autorité de santé (Francia) https://www.has-sante.fr/portail/

- CPG Infobase: Clinical Practice Guidelines (Canada) https://www.cma.ca/En/Pages/clinical-practice-guidelines.aspx

- SIGN: The Scottish Intercollegiate Guidelines Network (Scozia)http://www.sign.ac.uk/

- NSW MINISTRY OF HEALTH: <http://www.health.nsw.gov.au>.

Then CPGs were searched in PubMed (6 August 2021) using the search terms “(cerebral palsy) AND (guidelines OR recommendations)”, with the following filters applied: English language; age 2-18 years; years of publication 2016-2021.

CPGs reported incomplete evidence regarding query 2, then a further search was implemented, including systematic reviews (SR), on the following databases: PubMed (6 August 2021), Cochrane and PEDro (3 December 2021).

PubMed search strategy was: (("Cerebral Palsy"[Mesh]) OR (Cerebral Palsy) AND (("Walking"[Mesh]) OR (gait OR walking OR postural control OR balance OR hand function OR bimanual )) AND (rehabilitation OR Exercise OR Movement OR "Exercise Movement Techniques" OR "Exercise Therapy" OR Training OR "Physical Exercise" OR "Physical Education Training" OR "Physical Endurance" OR "Physical Fitness" OR ((aerobic OR strength* OR flexibility OR stretch* OR weight* OR resist*) AND (exercise* or training or activit*)) OR "Physical Therapy Modalities" OR physiotherap* OR "physical therapy" OR "physical Activity" OR "physical activities" OR Fitness OR "Goal directed" OR "Goal directed training" OR "functional training" OR "Functional Activity" OR "Functional Activities" OR "Context focused therapy" OR "Activity focused" OR "goal focused" OR "task oriented" OR "Home programme" OR "Home Exercise Programme" OR "Home programmes" OR "Home Exercise Programmes" OR "Neurodevelopmental Therapy" OR NDT OR "Play Therapy" OR "progressive resistance training" OR "functional strength training" OR "Muscle Strenght" OR "Muscle strengthening" OR "Muscle Training" OR "Therapeutic Exercise" OR "Interventions"). Filters applied were relative: Guideline, Meta-Analysis, Practice Guideline, Systematic Review; years of publication 2019-2021; English language; Age 2-18 years.

PEDro search strategy was: “Cerebral Palsy” in Abstract/Title and Topic; systematic reviews; years of publication 2019-2021.

Cochrane database search strategy was: “cerebral palsy” in Title, Abstract and Keyword; years of publication 2016-2021.
