# Supplementary material for: Evidence-based management and motor rehabilitation of cerebral palsy children and adolescents: a systematic review
Source: Front Neurol. 2023 May 25;14:1171224. doi: 10.3389/fneur.2023.1171224 (PMC10248244; doi:10.3389/fneur.2023.1171224)
Supplement: Supplementary file 2 [file Data_Sheet_2.docx]

**Supplementary Digital Material 2: Supplementary Table 3 online content only.**

Quality assessment of included systematic reviews through AMSTAR 2 (Assessing the Methodological Quality of Systematic Reviews).

| Systematic reviews | Items | | | | | | | | | | | | | | | | |
| --- | --- | --- | --- | --- | --- | --- | --- | --- | --- | --- | --- | --- | --- | --- | --- | --- | --- |
|  | 1 | 2* | 3 | 4* | 5 | 6 | 7* | 8 | 9* | 10 | 11* | 12 | 13* | 14 | 15* | 16 | Degree of affidability |
| Abdelhaleem 2021 | Yes | Yes | Yes | Yes partially | Yes | Yes | Yes | Yes | Yes | No | Yes | Yes | Yes | Yes | Yes | Yes | High |
| Alahmari 2020 | Yes | Yes partially | Yes | Yes partially | No | Yes | Yes | Yes partially | Yes | No | Yes | Yes | Yes | Yes | Yes | Yes | Moderate |
| Alamer 2020 | Yes | Yes | Yes | Yes | Yes | Yes | Yes | Yes | Yes | No | NC | NC | Yes | Yes | No | Yes | Low |
| Albuquerque de Araujo 2019 | Yes | Yes | Yes | Yes | Yes | Yes | Yes | Yes | Yes | No | Yes | No | Yes | Yes | No | Yes | Low |
| Armstrong, 2019 | Yes | Yes | Yes | Yes Partially | Yes | Yes | Yes | Yes | Yes | No | Yes | Yes | Yes | Yes | Yes | Yes | High |
| Bania 2019 | Yes | No | Yes | Yes | Yes | Yes | Yes | Yes | No | No | Yes | No | Yes | Yes | No | Yes | Very low |
| Beckers 2020 | Yes | Yes | Yes | Yes | Yes | Yes | Yes | Yes | Yes | No | NC | NC | Yes | Yes | No | Yes | Low |
| Betancourt 2019 | Yes | Yes | Yes | Yes | Yes | Yes | No | Yes | Yes partially | No | Yes | Yes | No | Yes | No | Yes | Very low |
| Chiu 2020 | Yes | Yes | Yes | Yes | Yes | Yes | Yes | Yes | Yes | Yes | Yes | Yes | Yes | Yes | Yes | Yes | High |
| Clutterbuck 2019 | Yes | Yes partially | Yes | Yes partially | Yes | Yes | Yes | Yes partially | Yes partially | No | NC | NC | No | Yes | No | Yes | Very low |
| Collado-Garrido 2019 | Yes | Yes | Yes | Yes | Yes | Yes | Yes partially | Yes partially | Yes | Yes | NC | NC | Yes | Yes | Yes | Yes | High |
| Corsi 2021 | Yes | Yes | Yes | Yes | Yes | Yes | Yes | Yes | Yes | No | NC | NC | Yes | Yes | No | Yes | Low |
| Das 2019 | Yes | Yes partially | Yes | Yes | Yes | No | Yes partially | Yes | No | No | NC | NC | No | No | No | Yes | Very low |
| De Guindos-Sanchez 2020 | Yes | Yes partially | Yes | Yes | Yes | Yes | Yes | Yes | No | No | Yes | No | No | Yes | No | Yes | Very low |
| Elbanna 2019 | Yes | Yes partially | Yes | Yes partially | Yes | Yes | Yes | Yes | Yes partially | No | Yes | Yes | Yes | Yes partially | No | Yes | Low |
| Elnahhas, 2019 | Yes | Yes | Yes | Yes | Yes | Yes | Yes | Yes | Yes | No | NC | NC | Yes | Yes | No | Yes | Low |
| Ghai 2019 | Yes | No | Yes | Yes | Yes | Yes | Yes | Yes | Yes | No | Yes | Yes | No | Yes | Yes | Yes | Low |
| Guchan 2020 | Yes | Yes | Yes | Yes | Yes | Yes | Yes | Yes | No | No | NC | NC | No | Yes | No | Yes | Very low |
| Han 2020 | Yes | Yes | Yes | Yes partially | Yes | No | Yes | Yes partially | Yes | No | Yes | Yes | Yes | Yes | Yes | Yes | Moderate |
| Hoare 2019 | Yes | Yes | Yes | Yes | Yes | Yes | Yes | Yes | Yes | Yes | Yes | Yes | Yes | Yes | Yes | Yes | High |
| Hsu 2019 | Yes | Yes | Yes | Yes | Yes | Yes | Yes | Yes | Yes | Yes | Yes | Yes | Yes | Yes | No | Yes | Low |
| Inamdar 2021 | Yes | Yes partially | Yes | Yes | Yes | Yes | Yes | Yes partially | Yes | Yes | Yes | Yes | Yes | Yes | Yes | Yes | High |
| Jackman 2020 | Yes | Yes | Yes | Yes partially | Yes | Yes | Yes | Yes | Yes | No | Yes | No | No | Yes | No | Yes | Very low |
| Johansen 2020 | Yes | Yes | Yes | Yes partially | Yes | Yes | Yes | Yes | Yes | No | Yes | Yes | Yes | Yes | Yes | Yes | High |
| Karadag-Saygi 2019 | Yes | Yes partially | Yes | Yes partially | No | Yes | Yes | Yes partially | Yes partially | Yes | NC | NC | Yes | No | No | Yes | Low |
| Liang 2021 | Yes | Yes | Yes | Yes | Yes | Yes | Yes | Yes | Yes | Yes | NC | NC | Yes | Yes | Yes | Yes | High |
| Lopez 2019 | No | Yes partially | Yes | Yes partially | Yes | Yes | Yes partially | Yes partially | No | No | NC | NC | No | Yes | No | Yes | Very low |
| Merino-Andres 2021 | Yes | Yes | Yes | Yes | Yes | Yes | Yes | Yes | Yes | No | Yes | Yes | Yes | Yes | Yes | Yes | High |
| Milne 2020 | Yes | Yes | Yes | Yes | Yes | Yes | Yes | Yes partially | Yes | Yes | Yes | Yes | Yes | Yes | No | Yes | Low |
| Montoro-Cardenas 2021 | Yes | Yes partially | Yes | Yes partially | Yes | Yes | Yes | Yes partially | Yes | No | Yes | Yes | Yes | Yes | Yes | Yes | High |
| Novak 2020 | Yes | Yes partially | Yes | Yes | Yes | No | Yes | Yes partially | Yes | No | NC | NC | Yes | Yes | Yes | Yes | Moderate |
| Ouyang 2020 | Yes | Yes partially | No | Yes partially | Yes | Yes | Yes | Yes | Yes | No | Yes | No | Yes | Yes | No | Yes | Low |
| Pin 2019 | Yes | Yes partially | Yes | Yes partially | No | No | Yes partially | Yes | Yes | No | NC | NC | Yes | Yes | No | Yes | Very low |
| Plasschaert 2019 | Yes | Yes partially | Yes | Yes | Yes | Yes | Yes | Yes partially | Yes partially | No | NC | NC | Yes | Yes | No | Yes | Low |
| Rathinam 2019 | Yes | Yes | Yes | Yes partially | Yes | Yes | Yes | Yes | Yes | No | NC | NC | Yes | Yes | No | Yes | Low |
| Ren 2019 | Yes | Yes partially | Yes | Yes | Yes | Yes | Yes | Yes | Yes | No | Yes | Yes | Yes | Yes | No | Yes | Low |
| Roostaei 2017 | No | No | Yes | Yes | No | No | No | Yes | Yes | No | NC | NC | Yes | Yes | No | Yes | Very low |
| Ryan 2017 | Yes | Yes | Yes | Yes | Yes | Yes | Yes | Yes | Yes | Yes | Yes | Yes | Yes | Yes | Yes | Yes | High |
| Salazar 2019 | Yes | Yes partially | Yes | Yes partially | Yes | Yes | Yes | Yes | Yes | Yes | Yes | Yes | Yes | Yes | Yes | Yes | High |
| Warnier 2019 | Yes | Yes partially | Yes | Yes partially | Yes | Yes | Yes | Yes partially | Yes partially | No | Yes | Yes | Yes | Yes | Yes | Yes | High |
| Wu 2021 | Yes | Yes partially | Yes | Yes | Yes | Yes | Yes | Yes | Yes | No | Yes | No | No | Yes | No | Yes | Very low |
| Yardımcı-Lokmanoglu 2020 | Yes | Yes partially | Yes | Yes partially | Yes | Yes | Yes | Yes partially | No | No | NC | NC | Yes | Yes | No | Yes | Very low |
| Zanon 2019 | Yes | Yes | Yes | Yes partially | Yes | Yes | Yes | Yes partially | Yes | Yes | Yes | Yes | Yes | Yes | Yes | Yes | High |

**Critical Items*
