# Supplementary material for: Evidence-based management and motor rehabilitation of cerebral palsy children and adolescents: a systematic review
Source: Front Neurol. 2023 May 25;14:1171224. doi: 10.3389/fneur.2023.1171224 (PMC10248244; doi:10.3389/fneur.2023.1171224)
Supplement: Supplementary file 3 [file Data_Sheet_3.docx]

**Supplementary digital material 3: supplementary table 4.**

Characteristics of included studies and evidence synthesis relative to query 1.

| Reference | Publication type | Quality assessment | Characteristics of the population | Outcome measures | Recommendations / Authors conclusions |
| --- | --- | --- | --- | --- | --- |
| 1. Spasticity in under 19s: management National Institute for Health and Care Excellence (NICE guidelines, 2012-2016; 2020) | CPG | AGREE II: high | CP children and young persons | / | "1.1.5 Offer a management programme that is:  developed and implemented in partnership with the child or young person and their parents or carers, individualized, goal focused.  1.1.6When formulating a management programme take into account its possible impact on the individual child or young person and their family.  1.1.8Identify and agree with children and young people and their parents or carers assessments and goals that:  -are age and developmentally appropriate  - focus on the following domains of the World Health Organization's International Classification of Functioning, Disability and Health:body functions,body structures, activities and participation,environmental factors.  1.2.2Offer a physical therapy (physiotherapy and/or occupational therapy) programme tailored to the child or young person's individual needs and aimed at specific goals, such as: enhancing skill development, function and ability to participate in everyday activities; preventing consequences such as pain or contractures  1.2.4When formulating a physical therapy programme for children and young people take into account:  -the views of the child or young person and their parents or carers  -the likelihood of achieving the treatment goals  -possible difficulties in implementing the programme  -implications for the individual child or young person and their parents or carers, including the time and effort involved and potential individual barriers.  1.2.5When deciding who should deliver physical therapy, take into account:  -whether the child or young person and their parents or carers are able to deliver the specific therapy  -what training the child or young person or their parents or carers might need  -the wishes of the child or young person and their parents or carers.  1.2.17 Reassess the physical therapy programme at regular intervals to ensure that: the goals are being achieved; the programme remains appropriate to the child or young person's needs" |
| 2. NICE guidelines: Managing cerebral palsy in under 25s 2021 http://pathways.nice.org.uk/pathways/cerebral-palsy | CPG | AGREE II: high | CP children and young persons | / | As a minimum standard of care, ensure that the young person has access to adults' services both locally and regionally that include healthcare professionals with an understanding of managing cerebral palsy. |
| 3. Management Of Cerebral Palsy in Children: A Guide for Allied Health Professionals (NSW Ministry of Health guidelines, 2018) | CPG | AGREE II: high | CP children and young persons | / | The guideline reflects what is currently regarded as a safe and appropriate approach to the management of children with cerebral palsy. However, as in any clinical situation there may be factors which cannot be covered by a single set of guidelines. This document should be used as a guide, rather than as a complete authoritative statement of procedures to be followed in respect of each individual presentation. It does not replace the need for the application of clinical judgement to each individual presentation. As in any clinical situation and due to the heterogeneous nature of cerebral palsy, there are factors that cannot be covered by a single guide. Clinicians and clients need to develop individual treatment plans that are tailored to the specific needs and circumstances of the client. This guideline should be read in conjunction with other relevant guidelines, position papers, codes of conduct, and policies and procedures, at professional, organizational and Local Health District levels.  A multiple disciplinary team approach (whether in a format of multidisciplinary, interdisciplinary or transdisciplinary), is considered best practice when working with children with complex needs. Research evidence supporting the effectiveness of a multiple disciplinary team approach is however, limited and shows conflicting results. Although a multiple disciplinary team approach may not be feasible in all settings, due to geographical, financial, organizational and time restraints, where possible, it is recommended that all disciplines involved with the child work together in a family-centered model to meet the needs and goals of the child and their family. Where clinicians may not work directly with, or have access to, other disciplines, they are encouraged to seek multidisciplinary support from other disciplines within their organization or geographical area, or from tertiary institutions or specialist services to facilitate the provision of a holistic service. Care must be taken to ensure that all professionals involved in a child’s care are included and this may include child care professionals and/or teachers. Particular attention should be focused on times of transition with early forward planning being essential for positive outcomes.  The functional motor ability classification scales* should be used to guide assessment and intervention with all children diagnosed with cerebral palsy to facilitate communication and goal setting. Further assessment should occur to ensure realistic goal setting, provide a baseline for therapy and for evaluation of therapy programs." |
| 4. SIMFER-SINPIA Intersociety Commission. Recommendations for the rehabilitation of children with cerebral palsy (Eur J Phys Rehabil Med, 2016) | CPG | AGREE II: low | CP children and young persons | / | Rehabilitation is a complex process aimed at promoting the best possible participation and quality of life for the child and for the family. Through direct and indirect actions, it focuses on the individual in all his dimensions, physical, mental, emotional, communicative and relational (holistic approach), and it involves the child’s family, social and environmental context (ecological approach).  The justified fields of intervention are determined on the basis of the data relating to the patient’s profile, and are related to:  — the architecture of the main functions (activities/abilities) on which to intervene for therapeutic purposes (the focus of the re-education plan);  —the types of these main functions, which can fall within the following areas: autonomic control, personal autonomy, locomotion, manipulation and praxis, sensation/perception and gnosis, cognition, communication, relationships;  —the compatibility of the therapeutic targets with the activities/abilities and the levels of participation appropriate to the age group considered;  —the priority functional activities/abilities and the levels of participation that the child with CP should, considering his specific age range, succeed in attaining; in other words the developmental stages (windows for intervention/critical periods). In this sense, the priority functional activities/abilities do not respect a predetermined hierarchical order (milestones), but change depending on the child’s age group. For example, walking is an important goal between the ages of 0 and 2 years and between the ages of 3 and 5 years and, in certain situations, can continue to be so between 6 and 8 years of age, but after this time it ceases to be an important goal, except in exceptional, justified circumstances. Conversely, the achievement of adequate autonomy in the sitting position becomes very important in children who use a manual or electronic wheelchair, an aid that, moreover, can be proposed for patients as young as 3 to 5 years of age, if they have a negative prognosis for walking….  The assessment of the patient must take into account not only the single functional area involved, but also its relationship with the other areas, so as to be able to define the overall level of development attained and the impact, on this, of the area in question. In seeking to characterize the elements comprising the different functional areas, it is important to provide not just a mere description of the phenomenon (it is present / it is not present / it is partially present / it is emerging), but also to state whether and in what way the child implements adaptive, compensatory or additional strategies, not least because these can serve as a crucial guide for the proposed therapy. |
| 5. Rosenbaum 2002 | Longitudinal cohort study | JBI: high | 657 uni-bilateral CP, mixed types, age 1-13 ys at first assessment, follow-up 4 ys. Gross | GMFM, GMFCS | Motor development curves were presented, describing average development predicted by the Gross Motor Classification System. Higher ability levels reached their limit of development in longer period than lower ability levels, though all levels reached their developmental limit by the age of 7 ys. |
| 6. Klevberg 2018 | Prospective longitudinal study | JBI: high | 60 unilateral, 42 bilateral CP, mixed types, MACS I-III, age at first assessment (mo): unilat. 25.0 (range 18–58), bilat. 35.0 (range 18–59); mean follow-up 4.5 months (range 5–51 mo). | AHA, B0HA, MACS | Although the AHA and BoHA are different tests, and the data are not directly comparable, children with bilateral CP seem to change their performance over time to a smaller extent than those with unilateral CP. Furthermore, children with bilateral CP seem to reach their developmental limits around 30 months of age, regardless of MACS level. More research is needed to further explore these findings, and particularly for children with bilateral CP, more knowledge is warranted to understand their specific challenges and to explore interventions that may potentially enhance their development. Nevertheless, our results call for early hand function interventions to facilitate the long-term development of bimanual performance, with particular attention to the grasping or holding ability and the asymmetric hand use at early age |
| 7. Eliasson 2022 | Prospective longitudinal study | JBI: high | 171 unilateral spastic CP, age range 18 mo-18 ys, mean follow-up 8 ys (range 1–17) | AHA, MACS | Previous knowledge that the AHA score at 18 months together with the MACS levels is predictive of future development was confirmed in this larger study. Children classified as having higher ability (MACS level I) had both a higher rate and limit of development and a shorter period of development than those having a lower ability (MACS level II). Children functioning in MACS level III had the lowest limit, and development occurred during the longest time. The stable performance lasted throughout adolescence for participants in all MACS levels from approximately 7 years. This demonstrates that, if the children have learned to use both hands in a meaningful way, they continue to use them when growing up. On an individual level, large variation in development is seen; therefore regular follow-ups for children in all MACS levels in the clinic are import-ant. Furthermore, the stabilizing of trajectories gives an important opportunity to shift the focus from capacity-related intervention to goal-directed training and participation interventions. There is positive evidence that children at any age and functional level can learn new skills. |
