# Supplementary material for: Evidence-based management and motor rehabilitation of cerebral palsy children and adolescents: a systematic review
Source: Front Neurol. 2023 May 25;14:1171224. doi: 10.3389/fneur.2023.1171224 (PMC10248244; doi:10.3389/fneur.2023.1171224)
Supplement: Supplementary file 4 [file Data_Sheet_4.docx]

**Supplementary digital material 4: supplementary table 5.**

Characteristics of included studies and evidence synthesis relative to query 2.

| Interventions | Reference | Publication type | Quality assessment | Characteristics of the treatment | Characteristics of the population | Outcome measures | Recommendations / Authors objectives and conclusions |
| --- | --- | --- | --- | --- | --- | --- | --- |
| 1. CHILD-FOCUSED THERAPY (GOAL/ TASK ORIENTED TRAINING) and CONTEXT-FOCUSED THERAPY | 1.1 Spasticity in under 19s: management National Institute for Health and Care Excellence (NICE guidelines, 2012-2016; 2020) | CPG | AGREE II: high | physical therapy (physiotherapy and/or occupational therapy), goal-oriented and task-focused therapy | CP children and young persons | / | 1.1.5 Offer a management programme that is: developed and implemented in partnership with the child or young person and their parents or carers, individualised, goal focused. 1.1.6When formulating a management programme take into account its possible impact on the individual child or young person and their family.  1.1.8Identify and agree with children and young people and their parents or carers assessments and goals that: -are age and developmentally appropriate - focus on the following domains of the World Health Organisation's International Classification of Functioning, Disability and Health:body functions,body structures, activities and participation,environmental factors. 1.2.2Offer a physical therapy (physiotherapy and/or occupational therapy) programme tailored to the child or young person's individual needs and aimed at specific goals, such as: enhancing skill development, function and ability to participate in everyday activities; preventing consequences such as pain or contractures 1.2.4When formulating a physical therapy programme for children and young people take into account: -the views of the child or young person and their parents or carers -the likelihood of achieving the treatment goals -possible difficulties in implementing the programme -implications for the individual child or young person and their parents or carers, including the time and effort involved and potential individual barriers. 1.2.11Consider task-focused active-use therapy such as constraint-induced movement therapy (temporary restraint of an unaffected arm to encourage use of the other arm) followed by bimanual therapy (unrestrained use of both arms) to enhance manual skills. 1.2.12When undertaking task-focused active-use therapy consider an intensive programme over a short time period (for example, 4–8 weeks). |
|  | 1.2 Management Of Cerebral Palsy In Children: A Guide For Allied Health Professionals (NSW Ministry of Health guidelines, 2018) | CPG | AGREE II: high | Child or goal-oriented therapy, context focused therapy | CP children and young persons | / | PAG.90 There are four main stages involved in goal directed therapy. The first is the formation of an age and developmentally appropriate goal. Goals should always be child focused to increase motivation. Assessment to identify the goal limiting factor(s) is a crucial next step. The task should then be analysed, considering the child’s skills as well as environmental limitations. Intervention should be structured and involve repetitive practice, appropriate adaptations to the task or the environment and outcomes evaluated using validated tools.  PAG.91 Context focused therapy consists of changing the task or the environment (but not the underlying body structure and function of the child) to promote successful task performance. In context focused therapy activities are identified which a child likes or needs to do, but has difficulty doing. The focus is then on changing the activity to make it easier to do by reducing restricting factors in the environment or the task. A child will practice activities within context, and individualised strategies are determined for each child and family member.  Therapy that focuses on changing the activity and the environment is considered as effective in improving functioning as therapy focusing on changing the child. |
|  | 1.3 Jackman et al. 2020 | SR: 74 RCT or quasi-RCT | AMSTAR 2:  very low | Active interventions were subcoded as “goal-directed”, “functional or part-task”, or “non-functional”.  The overall duration of the treatment was considered in terms of hours of face-to-face therapy and home practice. | CP subjects or high risk of CP, age 0-18 years. | AHA, COPM. | To improve individual goals, children need to practice goals for more than 14–25 hr, combining face-to-face therapy with home practice. To improve general upper limb function (based on evidence in the unilateral population), children need to practice for more than 30–40 hr. Interventions that set functional goals and involve actual practice of those goals lead to goal achievement at a lower dose than general upper limb motor training.  Dose of practice is one important factor in considering the ideal upper limb intervention for children with CP. Children are likely to achieve individual goals, if they have set their own goals and have practised those goals for more than 14 to 25 hr. To improve motor ability, a higher dose of practice is needed, likely 30 to 40 hr of practice.  If the purpose of the intervention is to achieve individual goals, goal-directed interventions, in which goals are practised, rather than focussing on underlying skills, are more effective.  For goal-directed or functional training, where the outcome was measured on the AHA, logistic regression showed that children under 8 years of age were 2 times more likely to succeed. On the COPM results were similar regardless of age, although children over 8 years were 1.46 times more likely to succeed. |
|  | 1.4 Novak et al. 2019 | SR: 1 SR, 3 RCT | AMSTAR 2: moderate | GOAL ORIENTED TRAINING  TASK ORIENTED TRAINING  CONTEXT FOCUSED THERAP | GMFCS I-III 4-18 yrs (1 SR e 2 RCT)  GMFCS I-III 4-18 yrs (2 RCT)  GMFCS I-IV, 11 mo–4 yrs (3 RCT) |  | ..the authors confirm data from previous SR (Novak et al.,2013: 5 RCT): “There are now numerous RCTs in the upper and lower limb, plus in early intervention, cognitive orientation to occupational performance, HABIT-ILE and in the home program evidence based, consistently indicating that goal directed training improves goal achievement of functional tasks involving motor performance. We upgraded the recommendations for use from weak positive to strong positive” to improve gross motor, hand function and self care.  Relative to TASK ORIENTED TRAINING the authors included into the analysis only 2 small RCTs that “conferred improved gross motor skills  compared to control non-task-based therapy. More research is needed”.  .. the authors reported “No between group differences for context focussed v child focussed v usual care” to improve self care in GMFCS levels I-V.  “Since context focused is equally effective to child focused, it is recommended that both approaches are used simultaneously, or the family selects the one that suits their preferences. Status update since 2013: The field has come to understand more about how to specifically train improvements in self-care skills through task specific training, goal-directed training and cognitive orientation to occupational performance. With this added data, the effect size of context focussed therapy is now less clear given that is compared to child-focussed therapy &/or usual care, which is an umbrella term for many different therapies with varying effect sizes. We have therefore downgraded the quality from high to moderate and recommendations for use from strong positive to weak positive.” |
|  | 1.5 Inamdar et al. 2021 | SR and meta-analysis: 12 RCT | AMSTAR 2: high | Neonatal developmental program vs No treatment/ Perceptual motor therapy vs Home program /COPCA vs TIP / NDT based trunk protocol vs Parent Infant Play /Task oriented approach or NFDR vs NDT/ Context-focussed vs Child-focussed /PMT + vibration vs Perceptual motor therapy / Physical therapy + trunk bel vs Physical therapy/ Physical therapy + balance boar vs Balance board  / NDT + electrical stimulation vs Electrical stimulation /NDT + kinesio-taping vs Kinesio-taping  intervention 1-2 times per week for 8 weeks or daily intervention for 2wks | Uni-bilateral CP, GMFCS I-V, 18 mo-puberty | GMFM, AIMS, IMP, AIMS, PEDI, BSID-MDI, videocoding, LSS | Object comparison of two physical therapy interventions to improve sitting ability or physical therapy plus adjunct versus physical therapy alone  Components of impairment remediation combined with functional balance training should be explored to improve sitting in children diagnosed with CP..  Task-specific, intensive, and child-initiated intervention components show promise for improving sitting in young infants at risk for CP. Given the benefits of early achievement of sitting, strong evidence-based research is needed. |
|  | 1.6 Hsu et al. 2019 | SR and meta-regression: 13 RCT | AMSTAR 2: MODERATE | Bobath vs Conventional therapy/ Motor learning coaching vs NDT/ Aims+Intensive - Goals+Intensive vs Aims + Conventional-Goals+Conventional/ Bike- Treadmill vs Conventional/ Task-oriented approach vs NDT / Intermittent physical therapy or NDT or Conductive education or Functional strength training or Sensory integration therapy or Functional strength training vs Conventional  /Treadmill vs Overground walking/Intensive NDT vs NDT  2-3 times per week, duration  ranged from 2 weeks to 6 months.  11 sessions  per week over 4 weeks to 6.5 h per day of intervention over  13 days were considered intensive physical training programs | GMFCS I-III, 1-17 yrs | GMFM scores | Objective: Effects of Therapeutic Exercise Intensity on CP. Meta-regression analysis revealed that the improvement in GMFM scores was positively associated with the number of daily training hours and program duration. |
|  | 1.7 Das et al. 2019 | SR: 34 SR | AMSTAR 2: very low | Intensive activity-based, goal-directed interventions any other treatments or control groups including placebo, sham therapy, or other PT interventions  Passive stretching, CIMT or mCIMT vs Bimanual training/interventions for improving gait speed/cardiorespiratory training programs/ intensive vs non intensive rehabilitation/ strength training/aerobic, treadmill/ virtual reality/hippotherapy/ equine-assisted activities and therapies/ aquatic intervention; taping/ early intervention/wholebody vibration/sensoryinformation | 0-20 yrs, mostly hemiplegic CP | unimanual  or bimanual  capacity and  performance,  achievement of  individualized  goals, or self-care  skills, range of motion,  spasticity, and  gait, grasp strength  MAS, JTHFT and caregiver  functional use  survey, Cohen’s  effect size, ICF, Various upperlimb  motor  outcome scales,  gait speed, Measures of  aerobic fitness  (peak  oxygen uptake  and 6-min walk  test, GMFM,  endurance, gait  speed, and stride  length), PEDI score,  Bertoti Posture  Assessment  Scale, EMG,BOTMP,  PDMS, and VABS, Griffith developmental  scales, AIMS , Bayley  Scales of Infant  Development,  Infant Motor  Profile, and  the Peabody  Developmental  Motor scales, Behavioral  Assessment  Scale, Hempel  Assessment,GAS,  COPM , bone density, force  plate and surface  electromyography, Reaching Test, House Functional  Classification, MUUL | Objective: to summarize and evaluate the evidence for the effectiveness of various therapeutic interventions employed in physiotherapy for children with CP.  Intensive activity-based, goal-directed interventions are more effective. The ability of manual stretching to increase range of motion and reduce spasticity is limited |
| 2. BIMANUAL TRAINING / HABIT | 2.1 Spasticity in under 19s: management National Institute for Health and Care Excellence (NICE guidelines, 2012-2016; 2020) | CPG | AGREE II: high | Bimanual therapy | CP | / | 1.2.11 Consider task-focused active-use therapy such as constraint-induced movement therapy (temporary restraint of an unaffected arm to encourage use of the other arm) followed by bimanual therapy (unrestrained use of both arms) to enhance manual skills. |
|  | 2.2 Management Of Cerebral Palsy In Children: A Guide For Allied Health Professionals (NSW Ministry of Health guidelines, 2018) | CPG | AGREE II: high | Bimanual training | CP hemiplegia | / | PAG. 88 Bimanual training provides an increased opportunity to practice bilateral activities with the goal of leading to an improved use of both hands during activity. Bimanual training involves practising the specific task or goal, or parts of the task, rather than focussing on the underlying body structure and function deficits. There is reliable evidence to support the use of bimanual therapy, with outcomes of bimanual therapy being equal to that of CIMT when the same amount of therapy is provided. Best candidates for bimanual training are typically older than 12 months, have spontaneous use of affected hand, selective motor control, have basic skills such as grasp and hold and have the cognitive skills to respond to cues. |
|  | 2.3 Alahmari et al. 2020 | SR and Meta-analysis: 4 RCT | AMSTAR 2: moderate | HABIT versus CIMT or structured and unstructured bimanual therapy  HABIT or BIMT vs CIMT  From 15 to 1h for 2 weeks | CP hemiplegia | AHA, JTTHF, GAS, MUUL, COPMT | Meta-analysis on the efficacy (measured by means of JTHFT) of HABIT versus CIMT or structured and unstructured bimanual therapy, HABIT showed a trivial effect compared to the other interventions, with an effect size of 0.06. Both groups performed functional tasks improving hand function within enjoyable and playful activities. |
|  | 2.4 Ouyang et al. 2020 | SR: 11 RCT, 1 quasi-RCT, 1 retrospective, 2 longitudinal studies. | AMSTAR 2: low | Individualised training, group-based training, or both, mostly in daily camp  Duration of intervention variable, generally 6h/die for 15 days | hemiplegic CP age 3-18 years | AHA, JTHFT, QUEST, ABILHAND-Kids, BBT, COPM, PEDI. | HABIT in the form of 6 h a day for 3 consecutive weeks (totaling 90 h) led to the improvement of bimanual ability, unilateral dexterity, self-care function, and functional goals after the intervention and that the improvements were mostly maintained during the follow-up period (duration not specified). HABIT with different dosages or added training components showed evidence for improving self-care function and functional goals, there was little impact on upper extremity function. |
|  | 2.5 Novak et al. 2019 | SR: 3 RCT | AMSTAR 2: moderate | CIMT versus OT or HABIT  Duration of intervention 90 h | CP hemiplegia 2-10 yrs |  | CIMT was equally effective for improving bimanual performance and unimanual capacity as dose matched OT or HABIT (bimanual training). All systematic reviews that compare CIMT with Bimanual Intensive Training concluded that both approaches led to similar improvements in upper limb function. Since CIMT is equally effective to bimanual, it recommended that both approaches are used, or the family selects the one that suits their preferences. |
| 3. CIMT | 3.1 Spasticity in under 19s: management National Institute for Health and Care Excellence (NICE guidelines, 2012-2016; 2020) | CPG | AGREE II: high | intensive programme over a short time period (for example, 4–8 weeks) | CP | / | 1.2.11 Consider task-focused active-use therapy such as constraint-induced movement therapy (temporary restraint of an unaffected arm to encourage use of the other arm) followed by bimanual therapy (unrestrained use of both arms) to enhance manual skills.  1.2.12 When undertaking task-focused active-use therapy consider an intensive programme over a short time period (for example, 4–8 weeks). |
|  | 3.2 Management Of Cerebral Palsy In Children: A Guide For Allied Health Professionals (NSW Ministry of Health guidelines, 2018) | CPG | AGREE II: high | mCIMT: the constraint is applied for up to two hours a day but for 2-10 weeks depending on the age | CP | / | PAG. 87 CIMT with children with cerebral palsy has involved the use of slings, mitts, splints and casts applied for most of the waking day, for a set period of weeks. Concerns regarding the intensity of the intervention has led to a modified model where the constraint is applied for up to two hours a day but for a longer overall duration. Outcomes of modified Constraint Induced Movement Therapy (mCIMT) have been shown to be just as effective as CIMT. The evidence indicates that mCIMT is more effective than usual care and the model of treatment appears to be age dependent. Under the age of 4 years shorter periods of daily practice at home and/or preschool over an 8 to 10 week period is effective while in children over 4 years of age intensive 2 to 3 week camps or group based intervention appears more effective. Higher intensity does not always result in better outcomes and CIMT is not age dependent, although children with poorer hand function do tend to make greater improvements. |
|  | 3.3 Hoare et al. 2018 | SR and meta-analysis: 36 RCT | AMSTAR 2: high | mCIMT  Frequency 2-7 days/week, duration of intervention sessions 0.5-8/hours per day, for a period of 1-10 weeks).  Low dose: total hours of intervention = 0 to 25 h  High dose: > 25 h | Unilateral CP, mean age 5.96 years (3 m- 19.8 yrs) | AHA, QUEST, MUUL, BBT, ABILHAND-Kids. | Objective: To evaluate the effect of constraint-induced movement therapy (CIMT) in the treatment of the more affected upper limb in children with unilateral CP.  The most common constraint devices were a mitt/glove or a sling (11 studies each). CIMT appears no more effective than another upper-limb therapy that is carried out intensively (most comparisons with intensive bimanual interventions that were therapist-led and more clearly defined). CIMT did not appear to impact body structure and function outcomes, such as grip strength, muscle stiffness and spasticity, and had no consistent effect on quality of life. Although there was minimal research on participation outcomes, it is hypothesised that CIMT and bimanual interventions may not have a direct effect on children's participation.  Two key ingredients across all models of CIMT are maintained: 1) restraint of the well-functioning upper limb (irrespective of device/type); 2) intensive, structured training (irrespective of type).  Clinicians, therefore, should view CIMT as a relatively short-term intervention that is provided for a defined period, and carefully evaluate outcomes before and aJer implementation using valid and reliable measures. CIMT appears to be a safe intervention for children with unilateral CP. Families should feel confident that, on average, active engagement in a well-defined, intensive  program of CIMT or bimanual therapy can lead to improvements in bimanual performance and unimanual capacity. This review was not able to identify the characteristics of children who could be advised to participate in one or the other of CIMT or bimanual interventions. In the meantime, clinicians should consider the specific goals for individual children and families and choose the most developmentally appropriate, family-centred, and convenient of these approaches (Hoare 2017). Factors, in addition to child and family characteristics and preferences, which may impact on intervention selection include therapist expertise, costs of implementing the intervention, funding and service delivery models, and resource availability. |
|  | 3.4 Novak et al. 2019 | SR: 2 SR | AMSTAR 2: moderate | CIMT versus OT | CP hemiplegia, 3 mo – 19 yrs | / | Objective: to investigate the effect of CIMT to improve bimanual performance and unimanual capacity in hemiplegic CP.  The authors strongly recommended CIMT to improve bimanual performance and unimanual capacity in hemiplegic CP. They also recommended CIMT to improve activity and participation: “CIMT conferred better activity and participation gains than no therapy, with large effect sizes. CIMT was equally effective to dose matched OT for producing activity and participation gains. Authors proposed the mechanism for improvements relates to intensity of practice rather than the type of intervention, consistent with the conclusions of previous reviews” |
|  | 3.5 Das et al. 2019 | SR: 34 SR | AMSTAR 2: very low | CIMT | 0-18 yrs, mostly hemiplegic CP. | Griffith developmental  scales, AIMS, Bayley  Scales of Infant  Development,  Infant Motor  Profile, and  the Peabody  Developmental  Motor scales  Neonatal  Behavioral  Assessment  Scale, Hempel  Assessment,  PEDIGAS, COPM  or a  nonstandardized  questionnaire | Objective : to investigate the effect of CIMT to improve the upper-extremity functioning.  There is positive support for the use of CIMT to improve the upper-extremity functioning |
| 4. HOME PROGRAMMES | 4.1 Spasticity in under 19s: management National Institute for Health and Care Excellence (NICE guidelines, 2012-2016; 2020) | CPG | AGREE II: high | Home programmes | CP children and young people | / | 1.2.5 When deciding who should deliver physical therapy, take into account:  -whether the child or young person and their parents or carers are able to deliver the specific therapy  -what training the child or young person or their parents or carers might need  -the wishes of the child or young person and their parents or carers. |
|  | 4.2 Management Of Cerebral Palsy In Children: A Guide For Allied Health Professionals (NSW Ministry of Health guidelines, 2018) | CPG | AGREE II: high | Home programmes | CP children and young people | / | PAG.90 There is strong evidence to support the effectiveness of home programs aimed at improving the performance of functional activities when based upon the following five step model.  1. Establish collaborative relationships between parents and therapist  2. Set mutually agreed upon family and child goals  3. Select therapeutic activities that focus on achieving family and child goals that are supported by best available evidence  4. Support implementation of home program through parent education, home visits and program updates to sustain motivation  5. Evaluate outcomes.  There is currently insufficient evidence to support the use of home programs aimed at improving participation. The use of appropriate outcome measures for evaluation is recommended. |
|  | 4.3 Beckers et al. 2020 | SR: 26 RCT, 4 single subject studies | AMSTAR 2: low | Home programmes.  Duration of programme: from 2 to 20 weeks, 6 months  Frequency of sessions : 7 h at week, 3 or 4 days/week or daily.  Duration of sessions variable: from 15min to 8h | CP uni/bilateral, GMFCS I-V, 4 mo-19 yrs. | AHA, BOTMP,  CAPE; CFUS, COPM,  EMG, GAS, GMFM-88, MGA, Maximum  Grip Aperture; nMT, nMU, PAFT; PDMS-2,; PEDI, PMGA, PV,  QUEST, RMS; RT, SFA,; SHUEE, Shriners WeeFIM, | Objective: To assess the feasibility and effectiveness of home-based occupational therapy and physiotherapy programmes in children with CP .  Conclusions about the effectiveness of home programmes cannot be made due to the large variability in the study, patient and intervention characteristics, comparators, and outcome measures used in the included studies. Even within the same treatment approach, frequency and duration of the interventions varied. As training intensity is an important predictor for treatment success, improvement in arm-hand function and performance can therefore not be solely attributed to the intervention approach. …  The question which motor learning approach in the specific context of parent-delivered programmes is best suitable, remains, therefore, unanswered…. Coaching of parents is a key element of home-based programmes. |
|  | 4.4 Novak et al.  2019 | SR: 2 RCT | AMSTAR 2: moderate | home programs vs therapy | CP GMFCS I-V, 4-13 yrs |  | Objective: to evalutate the effect of home programs on children with CP “Home programs conferred improved function compared to control no therapy…they are an effective way to increase the dose of therapy.” |
| 5. AOT | 5.1 Abdelhaleem et al. 2021 | SR and meta-analysis: 12 RCT | AMSTAR 2: high | AOT +/- CIMT or vs Whole-body vibration + conservative vs conventional physical therapy  From 9 to 30 minutes/each clip. 3- 5 days/week  For from 3 weeks to 2 months | uni/bilateral CP, 5-15 yrs | MA-2, GMFM-66, AHA, MUUL, ABILHAND Kids, AHA, ABILHAND-Kids, FMRI, ML and VT acceleration test, JTHF, BBT, Grip strength, QUEST, MAS, MMT, IPT, CHEQ, FTSTS, PBS, TUG, PRT, 10MWT, 6MWT, TUDS, UL-3DMA evaluation, EEG, Ankle ROM and MTS, PRT | Objective: To evaluate the evidence of using AOT in the rehabilitation of children with CP.  No evidence of benefit had been found to draw a firm conclusion regarding the effectiveness of AOT in the rehabilitation of children with CP due to limitations in methodological quality and variations between studies |
|  | 5.2 Alamer et al. 2020 | SR: 9 RCT | AMSTAR 2: low | AOT+ /- RP or +/- mCIMT vs video with no motor specific content or vs PT or vs mCIMT+ placebo  From 12 to 60 minutes per day , 3 -5days/week , for from 3 weeks to 6 months | CP hemiplegia, GMFCS I-IV, 3-12 yrs | WeeFIM , JTHF, BBT, MUUL, Motor control parameters (RU, RGV), AHA, MAS, MA2 ABILHAND- Kid, Grip and grasp power ABILHAND Kids, QUEST | Objective: to evaluate and examine the current best evidence for the effectiveness of AOT on upper limb function rehabilitation in children with hemiplegic CP. Taken together, these findings suggest action observation therapy was found to be a better rehabilitative intervention for upper limb rehabilitation to improve physical function and structure, activities, and participation of children with hemiplegic CP than simple motor training. However, considerable attention must be paid when we use AOT for CP children, due to the severity of motor impairment, and cognitive status; further studies are needed to determine the optimal frequency, intensity, and time of AOT on these particular study subjects |
|  | 5.3 Novak et al.  2019 | SR: 2 RCT | AMSTAR 2: moderate | Duration of intervention 1 hr/day for 15 days, for 3 wks | unilateral spastic CP, ambulatory 5-15 yrs |  | Objective: Improve bimanual hand  function AOT vs watching videos.  Upper limb action observation training conferred better bimanual performance compared to watching videos, but with a small effect size |
| 6. HABIT-ILE | 6.1 Novak et al. 2019 | SR: 2 RCT | AMSTAR 2: moderate | Duration of intervention: 90 hrs in camp format | Unilateral CP, GMFCS I-IV, 6-16 yrs | AHA , ABILHAND-Kids, Pediatric Evaluation of Disability Inventory, BBT, 6MWT, ABILOCO, Assessment of Life-HABITs. | Objective: To determine the efficacy of HABIT-ILE for children with unilateral spastic CP. Low evidence of improved motor function in both lower and upper limbs compared to usual care. |
| 7. ADAPTED PHYSICAL THERAPY / PHYSICAL ACTIVITY | 7.1 Spasticity in under 19s: management National Institute for Health and Care Excellence (NICE guidelines, 2012-2016; 2020) | CPG | AGREE II: high | Physical therapy and strenghtening | CP children and young people |  | 1.2.13 Consider muscle-strengthening therapy where the assessment indicates that muscle weakness is contributing to loss of function or postural difficulties.  1.2.14 Direct muscle-strengthening therapy towards specific goals using progressive repetitive exercises performed against resistance.  1.2.15 Following treatment with botulinum toxin type A, continuous pump-administered intrathecal baclofen, orthopaedic surgery or selective dorsal rhizotomy, provide an adapted physical therapy programme as an essential component of management.  1.2.16 Ensure that children and young people and their parents or carers understand that an adapted physical therapy programme will be an essential component of management following treatment with botulinum toxin type A, continuous pump-administered intrathecal baclofen, orthopaedic surgery or selective dorsal rhizotomy |
|  | 7.2 Management Of Cerebral Palsy In Children: A Guide For Allied Health Professionals (NSW Ministry of Health guidelines, 2018) | CPG | AGREE II: high | Physical therapy, strenghtening, fitness training,  gait training | CP children and young people |  | PAG 79. Strengthening programs and resistance training is an accepted intervention for children with cerebral palsy, in particular lower limb strengthening. Various systematic reviews of the literature into strengthening indicate that the  effectiveness of strength training, particularly in the lower limbs, is still disputed but that clinical practice continues despite the lack of published evidence. The literature also indicates there are no adverse increases in spasticity arising from strength training programs. There is limited evidence regarding the effects of strengthening programs on activity  and participation level outcomes. A critically appraised topic investigating strength training in the upper limbs of children with cerebral palsy found limited evidence to suggest that strengthening programs may increase upper limb strength in  children with cerebral palsy. Strength training/ progressive resistance exercise is based on three principles:  • To perform a small number of repetitions until fatigue  • Allow sufficient rest between exercises for recovery  • To increase the resistance as the ability to generate force increases.  Strengthening programs applied to children with cerebral palsy can be based on the guidelines published by The American Academy of Paediatrics and the National Strength and Conditioning Association (NSCA). Due to its intensity and the need for the muscles to rest and recover, it is not meant to be performed frequently and for long durations. Strength training needs to be combined with other activity-based programs such as treadmill training or cycling where you can look at other aspects of function such as endurance or coordination.  PAG. 90 Fitness training. Exercise can be defined as ”planned structured activities involving repeated movement of skeletal muscles that result in energy expenditure to improve or maintain levels of physical fitness”. Physical fitness is ”a set of attributes that people have or achieve that relates to the ability to perform physical activity”. The major components of health related fitness are cardiorespiratory fitness and muscle strength. The primary and secondary impairments of cerebral palsy affect both cardiorespiratory fitness and muscle strength contributing to reduced physical fitness. Current evidence suggests that fitness training to improve  aerobic fitness provides short-term benefits for clients with sufficient motor skills to be able to undertake training and any increase in capacity following training is not maintained when training stops. There is currently insufficient evidence to support the use of fitness training to improve function and participation however the research would suggest that aerobic fitness does not translate into either activity or participation gains. Frequency and intensity of interventions vary across the literature and generally focus on structured moderate to vigorous exercise. Attention is shifting with a growing recognition of the importance of reducing sedentary behaviour and encouraging light intensity activities throughout the day. It is recommended that fitness training to improve aerobic fitness, muscle strength and the general  health of children with cerebral palsy should be integrated into the child’s daily life on an ongoing basis.  PAG.88 Gait training is the process of first learning or re-learning how to walk after an intervention such as orthopaedic surgery and can be used as a therapy intervention for persons with cerebral palsy.  It can be achieved in a number of ways, but repetition of the actual motions/gait pattern performed during walking is the most important factor. Depending on the severity of the person’s impairment, one or more physiotherapists may be  present to assist in maintaining the person’s appropriate posture and moving their lower limbs to assist in facilitating the prerequisites of a normal gait pattern. Parallel bars may be used to help with gait training, especially in the early stages of rehabilitation as the bars provide support for the  child, and the clinician facilitates the desired movement. Other equipment, such as high support and low support assistive mobility devices are also utilised. |
|  | 7.3 Corsi et al. 2021 | SR: 13 RCT | AMSTAR 2: low | FES on gluteus, gastrocnemius+soleus, dorsiflectors or quadriceps/ Extracorporeal shock wave/ Gait training + virtual Reality+ tDCS/ overground gait and treadmill or lokomat training/ resistance training program/ vibration therapy/ serial casting  vs no intervention or conventional therapy or usual orthotic protocol  Session  frequency  (days/week): 1 or 2 or 3 or 5 or 6 or 7 days/week | Uni-bilateral CP, GMFCS I-III, 7-18 yrs | 3 d motion analysis, 10 MWT, 6 MWT, gait pattern, TUG | Objective: To investigate the effect of physical therapy interventions on spatiotemporal gait parameters in childrenwith CP.  Vibratory platform, gait training, electrical stimulation, and transcranial stimulation were effective to improve spatiotemporal gait parameters, especially velocity in children with cerebral palsy. Isolated strength training was not effective to improve gait parameters in Cerebral Palsy. |
|  | 7.4 Liang et al. 2021 | SR and meta-analysis: 27 RCT | AMSTAR 2: low | Resistance or mixed or aerobic training vs normal activity or usual FT | Uni-bilateral, GMFCS I-III, mean age 1.8-16 yrs | GMF ; gait speed; muscle strength, | Objective: to evalute effectiveness of exercise interventions for children with CP  Exercise interventions (resistance or aerobic or mixed training) may have beneficial effects on gait speed and muscle strength, but no significant effect on gross motor function in children with CP. |
|  | 7.5 Merino-Andres et al. 2021 (Cho et al. 2020) | SR and meta-analysis: 27 RCT(same studies included in Ryan et al. 2017, except Cho et al. 2020, which is included only in meta-analysis regarding “Balance” as outcome: 25 bilateral CP). | AMSTAR 2: high | strenght training program vs vibratory plat or Active conventional treatment/ HICT or treadmill  from 5 to12 wks-  2-3 days/wk-  20/ 30/45 /60 min | Uni-bilateral CP, GMFCS I-IV, 3-22 yrs | CAPE, PedsQoLCP  PierrsHarris Self Concept Scale, COPM, Stability (Global, A-P, Mid-lateral), Nº daily steps, Sitting time  Mc force, Knee ext. Force  Mc structure US  Dynamic Balance (FRT)  GMFM-88, Lower limbs mc force, TST  Gait speed, WB-SI  Gait analysis, Nº oscillations, Duration oscillations, Degrees first swing excursion,  Ankle ROM Torque,  6MWT PCI, Hip and knee strength  STS, LSU, MAS, TUG, Peak force , Maximum work repetition, Total work, Average power, Agonist to antagonist ratio,  RM, BOTMP, 1MWT | Objective; investigates the effects of strength training program in children and adolescents with CP to improve function, activity, and participation Significant standardised mean differences in favour of strength training program compared to other physical therapy technique(s) or untreated control group(s) for muscle strength at the knee flexors, at the knee extensor, at the plantarflexors, maximum resistance, balance, gait speed, GMFM (global, D and E dimension) and spasticity. |
|  | 7.6 Bania et al. 2019 | SR and meta-analysis: 9 RCT | AMSTAR 2: very low | Activity training on the ground (nintendo wii, Motor learning coaching, climbing training, Oriented circuit training, task oriented training, Mirror neurons stimulation-based video) vs. no or other intervention (Neurodevelopmental treatment/usual therapy)  20/30/45/50/60 min x 2-5/week for 4-6-8 or 12 | GMFCS I-III, 2 (most were over 6)-18 yrs | GMFM-66, 1MWT, TUG, MAS, wee fim, walking speed | Objective: effectiveness of activity training on the ground on activity or participation in CP children.  Activity training (whole -body self-initiated activities such as sitting, turning, sit-to-stand, walking, stepping, stair climbing, or other similar activities people use to transfer indipendently or with a handheld support in home or outdoor settings) compared to no treatment or usual treatment (NDT or strenghtening): no statistically significant difference. Low evidence. |
|  | 7.7 Armstrong et al. 2019 | SR and meta-analysis: 5 RCT, 1 quasi-RCT, 1 comparison trial, 1 pre–post study with a control group, 1 single-group study  with a control period. | AMSTAR 2: high | cycling vs conventional therapy, elliptical or treadmill  from 4 weeks to  6 months, frequency from 3 to 5 days per  week and session duration ranged from 20 to 90 minutes.  The overall dose of cycling ranged from 3 to 20 h | GMFCS I-V, uni-bilateral CP- mean age 10.4 yrs (SD 2.3) | GMFM-66 and -88, BOTMP, 30sWT, 600-yard walk–run test, Gait speed – selfselected, gait speed/fast, 3MWT, UE angular displacement  during gait, speed of excercise, speed/duration of exercise, PedsQL, PODCI global, PEDI-CAT | Objective: to determine the efficacy of cycling to improve function, reduce activity limitations in children with CP. Cycling can improve aerobic fitness, muscle strength, balance and gross motor function in children with CP; however, optimal training doses are yet to be determined. There was insufficient data to determine whether functional improvements can be retained. Conclusions were limited by small sample sizes, inconsistent outcome measures and a lack of follow-up testing. |
|  | 7.8 Lopez et al. 2019 | SR: 1 case study, 10 clinical trials (3 RCT), 3 pilot studies | AMSTAR 2: very low | of dance and movement with  music (rhythmic auditory stimulation [RAS])  Twice per week , from 10 to 50 min of dance training for from 4 to 12 weeks | Children and adults with CP | GMFCS, POMS, RSE, TUG, 10 MWT, Berg Balance Scale, EPS pressure platform, Electronic metronome (70-90bpm),Questionnaires for children, therapists, and parents based on the LIFE-H questionnaire,  PBS, QUEST, FIM,  GMFCS, WHODAS | Objective: to valute the efficacy of the use of dance and movement with music (rhythmic auditory stimulation [RAS]) in the neurorehabilitation of children and adults with CP. The potential for dance and Rhythmic auditory stimulation (RAS) to have positive impacts on body functions, emotional expression, social participation, and attitudinal change are indicated areas for consideration in future research. |
|  | 7.9 Das et al. 2019 | SR: 34 SR | AMSTAR 2: very low | Interventions  for improving  gait speed/ cardiorespiratory  training  programs/ intensive vs  nonintensive  rehabilitation/ strength training; aerobic/treadmill/virtual reality/ hippotherapy/ equine-assisted  activities and  therapies/aquatic  intervention/ taping/ early intervention/ wholebody  vibration/ sensory  information | 0-18 yrs, mostly hemiplegic CP | MAS,JTHFT and caregiver  functional use  survey, Cohen’s  effect size, ICF,  Gait speed, Measures of  aerobic fitness  (peak  oxygen uptake  and 6-MWT), GMFM,  endurance, gait  speed, and stride  length PEDI score,  Bertoti Posture  Assessment  Scale, EMG,BOTMP,  PDMS, and VABS, Griffith developmental  scales, AIMS , Bayley  Scales of Infant  Development,  Infant Motor  Profile, and  the Peabody  Developmental  Motor scales, Behavioral  Assessment  Scale, Hempel  Assessment,GAS,  COPM , bone density, force  plate and surface  electromyography  Reaching Test,  House Functional  Classification, MUUL | Objective: evaluate the effectiveness of physiotherapy interventions in children with CP  Intensive activity-based, goal-directed interventions are more effective. The ability of manual stretching to increase range of motion and reduce spasticity is limited. Gait speed can be improved most effectively by gait training |
|  | 7.10 Collado-Garrido et al. 2019 | SR: 12 RCT, 3 non RCT. | AMSTAR 2: high | Functional strength therapy vs no or conventional therapy  From 4 to 12 ws,  2 or 3 times/week, from 20 to 90 min/session | Uni-bilateral CP, GMFCS I-V, 4-18 yrs | GMFM, TUG, gait speed, | Objective: impact of resistance therapy on motor function in children with CP…statistically significant positive effect on motor function in favor of the use of resistance therapy in weakened musculature in children with CP. Resistance therapy would not only increase the strength of the musculature of children with CP, but this increased strength would also have an impact on the motor function. Nevertheless, it is possible that the between-group effect may have been overestimated due to the existence of a publication bias. Necessary to review and modify the games and exercise used to perform the resistance therapy, so as guarantee adherence. |
|  | 7.11 Clutterbuck et al. 2019 | SR: 34 studies (17 RCT). | AMSTAR 2: very low | Active exercise interventions (Gross Motor Activity Training alone or with progressive resistance exercise plus additional physiotherapy, Physical Fitness Training, Modified Sport, Non-Immersive Virtual Reality) vs NDT/ Conventional  movement normalisation/no intervention  From 15 min to 90 min  Total dose: 3-5/  week, for 12 weeks to 6 months | CP mixed types, GMFCS I-IV (mostly I-III), 3-18 yrs | GMFM-66, PEDI, MAS: Mob-Que28, MABC-2:; BOMPT;  Wee-FIM; AMPS; BOT-2; Aquaa, CAPE | Objective: effectiveness of active exercise interventions for improving gross motor activity/participation of school-aged,ambulant/semi-ambulant children with CP. Active exercise interventions improve gross motor function of ambulant/semi-ambulant children with CP. Gross Motor Activity Training is the most common and effective intervention. Practice variability is essential to improve gross motor function. Participation was rarely measured and requires further research, particularly in interventions that embed real-world participation opportunities like Modified Sport. |
|  | 7.12 Novak et al. 2019 | SR: 6 SR | AMSTAR 2: moderate | aerobic exercise (including cycling and treadmill) vs . Eclectic group of interventions including Nintendo, wall climbing, sit to stand, circuit training of functional tasks, overground or treadmill walking.  Modified sport | Fitness. GMFCS I-II  Mobility training GMFCS I-IV, 3-21 yrs  Modified sport CP mixed types, GMFCS I-III, 4-16 yrs  Physical activity CP mixed types, GMFCS I-V, <25 yrs  Strenght training Mixed CP types, GMFCS I-III, 3.4-20 yrs |  | Objective : effectivness of fitness and other gross motor interventions on children with CP.  *Fitness*. Moderate-based evidence (including Ryan 2017). Effective for children GMFCS I-II who can move fast enough to train aerobic fitness. … aerobic exercise (including cycling and treadmill) conferred improved gross motor function in the short and intermediate term, did not improve gait speed.  *Mobility training*. 6 SR. GMFCS I-IV, 3-21 yrs. Low to moderate-level evidence of improving gait speed and gross motor function. Eclectic group of interventions including Nintendo, wall climbing, sit to stand, circuit training of functional tasks, overground or treadmill walking.  *Modified sport*. Observational studies. CP mixed types, GMFCS I-III, 4-16 yrs. Very low-level evidence of improving gross motor skills, gait speed, aerobic fitness.  *Physical activity*. 4 SR. CP mixed types, GMFCS I-V, <25 yrs. Low-level evidence, conflicting results on improving gross motor function, gait and fitness.  *Strenght training*. 4 SR. Mixed CP types, GMFCS I-III, 3.4-20 yrs. Strength Training conferred improved muscle strength and gait. |
|  | 7.13 Ryan et al. 2017 | SR and meta-analysis: 29 RCT | AMSTAR 2: high | 8 RCT compared aerobic exercise to usual care, 15 compared resistance training to either usual care or no treatment, 4 compared mixed training to usual care or no treatment, 2 compared aerobic exercise to resistance training | CP mixed types, GMFCS I-V <19 yrs | GMFM, daily phisical activity, gait speed | Aerobic exercise may improve activity as indicated by motor function but does not appear to improve gait speed, walking endurance, participation or aerobic fitness among children with CP in the short or intermediate term. There is no research regarding the effect of aerobic exercise on participation or quality of life. Resistance training does not appear to improve motor function, gait speed or participation in the short or intermediate term, or quality of life in the short term, in children and adolescents with CP but may improve muscle strength. Mixed training does not improve motor function or gait speed but does improve participation in children and adolescents with CP in the short term. No difference between aerobic and resistance training on motor function but a difference in muscle strength in the short term. Although the evidence suggests that exercise might be safe for people with CP, only 16 trials (55%) included information on adverse events; these trials reported no serious adverse events. All the studies had small numbers of participants, meaning that the results may not be accurate |
|  | 7.14 Elnahhas et al. 2019 | SR: 7 RCT | AMSTAR 2: low | Backward gait training +/- partial body weight support  on treadmill vs  no intervention  from 15 minutes to 25 min, 3/  week , for 6 to 12 weeks | Uni-bilateral spastic CP, GMFCS I-III, 5-14 yrs | Dynamic balance, Walking velocity using, Step symmetry presented by  time spent on each foot, Spatiotemporal gait parameters via 3D gait analysis system as step length (m),  walking velocity (m/s), cadence  (steps/minutes), percentage of  the stance & swing phases of  the affected LL in relation to  gait cycle (%),  GMFM-88 | Objective: Effects of backward gait training on balance, gross motor function, and gait in children with CP. Moderate evidence that backward gait training improves mobility (gait) and some evidence that backward gait training improve balance and gross motor function. |
|  | 7.15 Albuquerque de Araujo et al. 2019 | SR and meta-analysis: 7 RCT. | AMSTAR 2: low | Balance training + NDT/Whole body vibrationtraining plus NDT/ Balance training+ NDT / Antigravity treadmill training+ NDT/ Wii therapy+ NDT/Hippotherapy + NDT vs NDT  From 20 min to 120 min × from 2 to 7/wk × from 8 to 12 wk | Uni-bilateral spastic CP, GMFCS I-II (incomplete data), 5-15 yrs | 6MWT, PBS, TUG, Overall/ anteroposterior/mediolateral stability index.Fall risk test, Cadence/Stridelength/velocity/time spent in double-limb support. | Objective: whether combining balance-training interventions with other active interventions enhances the effects of the active intervention alone on postural control of children and adolescents with CP.  Very-low quality evidence suggests that balance-training interventions (i.e., activities that cause unpredicted perturbations, such as unstable or mobile surfaces, in multiple training settings) combined with other intervention enhances the effect of the other intervention alone on postural control at short-term. |
|  | 7.16 Inamdar et al. 2021 | SR and meta-analysis: 12 RCT. | AMSTAR 2: high | Several comparisons: Neonatal developmental program vs No treatment / Perceptual motor therapy vs Home program/  COPCA vs TIP /  NDT based trunk protocol vs Parent Infant Play /  Task oriented approach vs NDT/  NFDR vs NDT / Context-focussed vs Child-focussed /PMT vibration + vs Perceptual motor therapy /  Physical therapy+ trunk bel vs Physical therapy only /  Physical therapy+ balance boar vs Balance board /  NDT + electrical stimulation vs Electrical stimulation /  NDT vs kinesio-taping vs Kinesio-taping  From 2 times per week to daily for from 2 to 8 weeks | Uni-bilateral CP, GMFCS I-V, 18 mo-puberty | AIMS, GMFM sitting, IMP, AIMS, PEDI, BSID-MDI, videocoding, GMFM, LSS | Objective: effectiveness of physical therapy interventions to improve sitting ability in young children with or at risk for CP. Components of impairment remediation combined with functional balance training should be explored to improve sitting in children diagnosed with CP..  Task-specific, intensive, and child-initiated intervention components show promise for improving sitting in young infants at risk for CP. Given the benefits of early achievement of sitting, strong evidence-based research is needed. |
|  | 7.17 Yardımcı-Lokmanoglu et al. 2020 | SR: 3 small RCT. | AMSTAR 2: very low | Standardized motor therapy+ Somatosensory therapy vs Standardized motor therapy/  Home-based program vs no control/  Traditional Physical Therapy+ Wholebody  Vibration vs Traditional Physical Therapy/  NDT+ Integrated  intensive proprioceptive  and visuomotor training vs NDT/  Usual care +  Sense intervention vs usual care | Spastic CP, GMFCS I-III, 5-15 yrs | Purdue Pegboard Test, Limb position sense, Digital dynamometer Tiltmeter, Tetrax Interactive Balance System, two-dimensional OptoGait  System 3D motion system, GAS, peds- QL, COPM, AHA, BBT | Objective: evaluate the proprioception treatment approaches and to investigate the effect of these approaches in individuals with CP.  Different approaches on proprioception (i.e., whole body vibration or integrated intensive proprioceptive  and visuomotor training) combined with conventional physiotherapy (CPT), showed no superiority on motor performance, compared to CPT alone. |
| 8. HYDROTHERAPY | 8.1 Management Of Cerebral Palsy In Children: A Guide For Allied Health Professionals (NSW Ministry of Health guidelines, 2018) | CPG | AGREE II: high | hydrotherapy | CP children and young persons | / | PAG. 96 The warmth and buoyancy of the water provides support which can aid pain relief, but also a different movement experience to that on land. The heat of the water may assist relaxation, or help reduce spasms. Walking may not only be easier but possible without aides for some children and young adults with cerebral palsy. Fitness and endurance can be more easily challenged in a controlled way. Hydrotherapy is also an excellent recreational pursuit which can lead to improved swimming skills, and respiratory function. … reported benefits such  as improved function for children with cerebral palsy, including better walking efficiency, improved strength, range of motion or balance. However further more robust studies were needed. |
|  | 8.2 Roostaei et al. 2017 | SR: 11 studies (2 RCT). | AMSTAR 2: very low | Acquatic intervention vs no intervention or no control group.  Frequency 2-3/week, from 30 to 60 min for session , duration 6–16 weeks | Uni-bilateral CP mixed types, GMFCS I-V, 3-21 yrs | GMFM, TUG, Gait velocity, COPM, 6 MWT, PEDI | Objective: effectivness of aquatic intervention on  gross motor skills for children with CP  Evidence on aquatic interventions for ambulatory children with CP is limited. Aquatic exercise is feasible and adverse effects are minimal; however, dosing parameters are unclear. Further research is needed to determine aquatic intervention effectiveness and exercise dosing across age categories and GMFCS levels. The aquatic setting, specifically type of pool, temperature of the water, and group or individualized instruction should be specified. |
|  | 8.3 Novak et al. 2019 | SR:  2 SR | AMSTAR 2: moderate | Hydrotherapy  2-3x wk for  6-16 wks | CP mixed types  GMCFCS I-V  3-21 yrs | / | Low-quality supporting evidence (including Roostaei 2017). Aquatic-based exercises improved vitals and gross motor function. |
| 9. TREADMILL/MECHANICALLY ASSISTED  WALKING | 9.1 Management Of Cerebral Palsy In Children: A Guide For Allied Health Professionals (NSW Ministry of Health guidelines, 2018) | CPG | AGREE II: high | Treadmill training | CP children and young persons | / | PAG.88 Treadmill training is an active approach to gait training in which the child practices the movement of walking on a treadmill rather than within the real-world environment. Treadmill training may include partial body-weight support, in which the child is placed in a harness that supports their weight, whilst a clinician manually guides the legs in a walking motion. Treadmill training, including those with partial body-weight support, are based on motor learning theories, in which the child carries out the activities of walking repetitively, with increasing speed and weight-bearing with the aim of this skill carrying over to walking within an everyday context. Combined results from four systematic reviews suggest that there is low quality evidence to support treadmill training to improve weight-bearing. It also found low quality evidence that treadmill training will improve functional walking although the practice of overground walking, rather than treadmill training may be more effective. |
|  | 9.2 Chiu et al. 2020 | SR and meta-analysis: 17 RCT. | AMSTAR 2: high | Any type of mechanically assisted walking training (e.g. treadmill,  Lokomat, gait trainer) in any direction (forward or backward) with  any support (with or without body weight support)vs  no or sham walking training or the same  dose of overground walking training  Duration of the intervention 4-12 weeks, intensity of training 15-40 minutes, frequency 2-5/week | Uni-bilateral CP, GMFCS I-IV, 4-14 yrs | 6 MWt, 10 MWT, GMFM, Faces Pain Scale, VAS, CAP, PedsQL | Object: to evalute Treadmill training on gait of children with CP. Compared with no walking, mechanically assisted walking training probably results in small increases in walking speed (with or without body weight support) and may improve gross motor function (with body weight support). Compared with the same dose of overground walking, mechanically assisted walking training with body weight support may result in little to no difference in walking speed and gross motor function, although two studies found that mechanically assisted walking training without body weight support is probably more effective than the same dose of overground walking training for walking speed and gross motor function. Not many studies reported adverse events, although those that did appeared to show no differences between groups. The results are largely not clinically significant, sample sizes are small, and risk of bias and intensity of intervention vary across studies, making it hard to draw robust conclusions. Mechanically assisted walking training is a means to undertake high-intensity, repetitive, task-specific training and may be useful for children with poor concentration. |
|  | 9.3 Han et al. 2020 | SR and meta-analysis: 8 RCT. | AMSTAR 2: moderate | PBWSTT + usual physical activity program vs  Usual physical activity program /  Over ground walking + usual physiotherapy /  TBWS + regular therapeutic treatment vs usual treatment / TGT + traditional physical therapy vs Traditional physical therapy / SSTTEP vs  Strengthening / TGT vs conventional therapy or  Over ground walking / BDTT vs stretching  From 15 to 30 min, 2-3 days per wk, for 6 to 12 wk | Uni-bilateral CP, GMFCS I-IV, mean age 4.5-16 yrs | 10-MWT,  Gait speed  Cadence  Gait stride length  Gait double limb support, PODCI, MAS, TUG, BBS,  Ankle joint Muscle-tendon  Total strain Stiffness | Objective: Effectiveness of treadmill training on gait function in children with CP. These findings suggested that treadmill training on cerebral palsy was effective for gait endurance, gait speed and limb support time. No significant improvement in cadence and step length. |
|  | 9.4 Novak et al. 2019 | SR: 3 SR. | AMSTAR 2: moderate | Treadmill training, with or without body weight support, | Uni-bilateral CP, GMFCS I-IV, 4-21 yrs | / | Treadmill training, with or without body weight support, conferred improved walking speed, endurance and gross motor function. |
| 10. VR - VIDEOGAMES | 10.1 Johansen et al. 2020 | SR and Meta-analysis: 8 RCT | AMSTAR 2: moderate | MCVG+/- NDT or UE therapeutic program or PT or usual program or NDT vs NDT or no tratment  from 15 to 45  min from 2 to 7 days/week, total amount of therapy  from 6 to 108 h. period from 6 to 16 weeks | GMFCS I-V, 5-20 yrs | QUEST  JTHFT  ABILhand  WeeFIM  mABC-2  BOTMP  1MWT  PDMS-2  9HPT  Dynamometer  MAS  MUUL  BBT  QUEST  TVPS-3 | Objective: To examine the effect of motion-controlled commercial video games.  The results highlight the potentials of videogames as a supplementary method of training of arm and hand function for persons with CP. Results should be interpreted with caution due to high risk of bias and low strength of evidence (positive: task oriented, motivating, intensive) |
|  | 10.2 Plasschaert et al. 2019 | SR: 2 studies | AMSTAR 2: low | BoNT-A  injections +/- occupational therapy;  SDR and occupational therapy/physical therapy  ITB and  physical therapy;  HABIT-ILE training;  Armeo Spring device training + occupational  therapy; transcranial magnetic stimulation; hyperbaric  oxygen therapy; Wii/virtual reality training;  sitting/standing positioning; and hippotherapy alone or vs occupational therapy or physical therapy  20 to 45 min, 2 to 5 days/wk, for 8-12 wks | Bilateral CP 0-19 years | MAS, COPM, QUEST, TVMS, ROM , PDMS -2, PEDI, MMT, Grasp strength, BOTMP, RIC-FAS-selfcare,   \| GMFM,  ABILHAND -kids,  BBT, JTTHF, MUUL,  VMI , TVPS,  NEPSY ® scale  -tapping,  Tablet time,  Visuomotor,  Jebsen Test 6 items,  M-ABC-2 total score ,  Manual dexterity,  Aiming and catching,  BOTMP ,  K- DTVP, elapsed time \| \| --- \| | Objective: to evaluate the efficacy of interventions on upper limb function in children with CP. Very low-level evidence about improvement in upper limb function. |
|  | 10.3 Rathinam et al. 2019 | SR: 6 RCT | AMSTAR 2: low | VR vs Physiotherapy  And occupational  Therapy/ Physiotherapy/ Strengthening  Exercise  30 min/session/  1 or 2 session/  wk/ from 4 to 6 wk | uni/bilateral CP, GMFCS I-V, 6-18 yrs | COPM, SPCC,  QUEST, MUUL, PMAL, BOTM, MACS, NHPT,  JTHFT | Objective: determine the effectiveness of VR as an intervention to improve hand function in children with CP. Four studies reported some improvement in hand function, but only one had a low risk of bias. .... The existing reviews reported the improved motivational factor and high parental satisfaction, but our study did not examine this component. Our review is in agreement with the other reviews that the available evidence from the existing studies was inconsistent and that VR cannot be reliably suggested to improve hand function until further studies have ascertained its therapeutic effect. |
|  | 10.4 Novak et al.  2019 | SR: 1 SR (19 RCT) | AMSTAR 2: moderate | VR vs NDT or usual care  Duration of intervention 20-90 mins/ day, 1-7x wk, over 4- 20 wks | CP 4-12 yrs | / | Objective: to evalute the effectivness of VR on improving arm function & postural control in children with CP. VR conferred better arm function than NDT or usual care, with large effect sizes…  Findings suggest that games are to be used as a complement to conventional therapies and not as a substitute. VR may also induce neuroplasticity. |
| 11. VR- GROSS MOTOR/ BALANCE | 11.1 Montoro-Cardenas et al. 2021 | SR: 11 RCT | AMSTAR 2: high | NWT +/- CPT vs non intervention or CPT  30 min and interventions lasting longer than 3 ws for 4–6 weeks | GMFCS I-IV, spastic uni/bilateral | PBS;  TUG;  OLST | Objective: To analyse the efficacy of Nintendo Wii therapy (NWT) on functional balance in children  with CP. Nintendo WII Balance (NWT) can be considered an effective treatment for improving functional and dynamic balance in children with CP, especially when combined with CPT (Conventional Physical therapy) in 30-minute sessions with interventions lasting longer than 3 weeks. Accessible and low cost. Incorporates motivation and fun. On functional balance we found very low quality evidence with large effect of NWT compared with no intervention and moderate quality evidence for using NWT (plus conventional) CPT versus CPT in session of approximately 30 min and interventions lasting longer than 3 ws; for dynamic balance very low quality evidence for a medium effect for using NWT plus CT versus CPT was found |
|  | 11.2 Wu et al. 2021 | SR: 11 RCT. | AMSTAR 2: very low | VR+/-  regular rehabilitation vs regular rehabilitation  15 - 40 min/time, 2 to 5  days/week, for 3 to 12 weeks | Hemiplegic, diplegic, quadriplegic CP. GMFCS I-IV but incomplete data about GMFCS levels, age over 6 yrs. | M-ABC, PBS, BBS, NintendoWii Fit Balance  Board Score; TUG, PBM | Objective: to evaluate the effect of VR games on balance recovery of children with CP.  VR games played a positive role in the improvement of balance of children with CP, but these results should be viewed with caution owing to current methodological defects |
|  | 11.3 Ren et al. 2019 | SR: 7 RCT | AMSTAR 2: very low | VRGs vs no intervention/  VRGs + traditional rehabilitation therapy vs traditional rehabilitation therapy only/  VRGs vs traditional rehabilitation therapy  17–40 min, 2 to 5 times, for 4 to 9 weeks (total duration 400 to 2400 min) | Hemiplegic, diplegic, quadriplegic CP. GMFCS I-V, age over 6 yrs. | GMFM-66;  NHPT; MABC | Objective: to evaluate the rehabilitatitive effect of Virtual Reality Games (VRGs) for gross motor skills of children with CP. Preliminary evidence shows that VRGs have positive effect on the improvement of gross motor skills of children with CP. Additionally, the single intervention time was 17–40 min, the intervention frequency should be over 5 times per week, the intervention cycle was over 12 weeks while the total intervention time should be more than 1000 min. VRGs essentially belongs to the functional training that based on the ecological theory and dynamical systems theory, which focus on the role of the environment and the task in the performance of functional activities |
|  | 11.4 Pin et al. 2019 | SR: 21 studies (10 RCT). | AMSTAR 2: very low | ICP +/- additional intervention to the conventional therapy  25 to 90 min/session,  1-5 sessions/day to 1-7 sessions/week, for 5 consecutive  days to 20 weeks in duration | GMFCS I-II, mean age over 4.8 yrs | Community Balance and Mobility Scale, 6MWT, TUG, GMFM, Test of Visual Perceptual  Skills, Postural Scale Analyzer, Functional mobility, COP, Medial-lateral and anteriorposterior  trajectory and velocity under eyes closed or eyes open when standing still, BBS, Fullerton Advanced Balance Scale, BOT-2, AHA, Lateral step-up, Half kneel-to-stand, GMFM, 10 MWT, 1MWT, Trunk Control Measurement Scale, Sitting Assessment for  Children with Neuromotor  Dysfunction, PBBS, BBT, QUEST, Visual-Perceptual Skills, MACS, Wee-FIM, Visual analogue scale to  grade level of head control. GAS, Chailey Levels of Ability, Sitting Assessment for  Children with Neuromotor  Dysfunction | Objective : to investigate the effectiveness of ICP (interactive computer play) in rehabilitation compared with the conventional therapies.  ICP seemed to be more effective than conventional therapy in improving postural control and balance, with medium to large effect sizes for children with mild to moderate severity of CP. Future studies of high methodological rigour are required to verify the role of on-site guidance of the children during ICP and the effect on children with more severe CP. |
|  | 11.5 Warnier et al. 2019 | SR and meta-analysis: 26 studies, 9 RCT. | AMSTAR 2: high | Virtual Reality Therapy (VRT)  intervention  with a game component or  real-time feedback or an interactive workout  in a virtual world  (total amount of therapy 60 to 4200 min)  15 to 90 min per session,  1 day to 20 weeks | GMFCS mostly I, one mixed, 6-18 yrs | mABC-2, 1 MWT, 6MWT, Romberg, Quiet stance (force plate), CB&M  TUDS, BOTMP, PBS, FRT, FAB, FFRT,  FSRT, PRT, TUDS, MAS¸ PRT, mSOT | Objective: To investigate the effect of Virtual Reality Therapy (VRT) on balance and walking in children with CP. VRT seems a promising intervention for rehabilitation in children with CP. The meta-analysis confirmed this positive effect. These results must be interpreted with caution due to differences in the interventions used, the lack of randomized-controlled trials, and the relatively small groups. |
|  | 11.6 Ghai et al. 2019 | SR: 14 RCT. | AMSTAR 2: low | VR vs conventional  approaches (resistance training, rhythmic auditory  cueing , robot-assisted training) | spastic or mixed uni/bilateral CP  age 6-16 years | gait velocity, stride length, cadence,  GMFM, FRT, sit to stand test, TUG, 10-MWT, QUEST, 10-stair climbing test, wee FIM, functional mobility scale, PBS | Objective: to analyze the influence of virtual-reality  training on gait recovery in children with CP.  88% of the studies reported significant enhancements in gait performance after training with virtual reality. Meta-analyses revealed positive effects of virtual-reality training on gait velocity (Hedge’s g = 0.68), stride length (0.30), cadence (0.66), and gross motor function measure (0.44). Subgroup analysis reported a training duration of 20–30min per session, ≤4 times per week across ≥8 weeks to allow maximum enhancements in gait velocity. |
|  | 11.7 Novak et al. 2019 | SR: 1 RCT, 5 observational study. | AMSTAR 2: moderate | Wii therapy and NDT vs to NDT  for 12 week | Hemipleglic spastic CP, 4-18 yrs  GMFCS I-III | / | Virtual reality + biofeedback appeared to confer better balance than virtual reality alone.  Wii Fit appeared to confer improved balance. |
|  | 11.8 Albuquerque de Araujo et al. 2019 | SR: 1 RCT | AMSTAR 2: low | BTI (dynamic platform, whole body vibration, WBV, hippotherapy Wii therapy; antigravity training) + NDT VS NDT  20 -30 min/day of BTI for a period of 8-12 weeks, 2 to 7 days/week | Spastic hemiplegic CP, mean age 9.6 yrs (SD 2.6), GMFCS I-II | 6MWT, PBS, TUG, Overall/anteroposterior/mediolateral stability index; fall risk test; Cadence/Stride length/velocity/time spent in double-limb support | Objective: to investigate the effect of balance-training interventions with active interventions on posturalcontrol of children and adolescents with CP.  Wii therapy and NDT, compared to NDT, improves PBS in short term (12 weeks). |
| 12. NIBS (rTMS or tDCS) | 12.1 Elbanna et al. 2019 | SR: 14 RCT (10 tDCS, 4 rTMS) | AMSTAR 2: low | tDCS or rTMS +/- treadmill training with or without VR vs sham rTMS or placebo or NDT or treadmill training  1 to 10 sessions twice/week | CP, traumatic brain injury or pediatric stroke ≤ 18 years | GMFM,PEDI, PBS, PROM, Ashworth scale, - Gait analysis | Objective: To assess the evidence of the effectiveness of NIBS for rehabilitation of pediatric motor disorders after brain lesion.  rTMS improves upper limb function after intervention. tDCS improves balance and majority of gait variables. Not adverse effects. High satisfaction levels for participants and caregivers. No long term follow-up. |
|  | 12.2 Corsi et al. 2021 | SR: 3 RCT | AMSTAR 2: low | tDCS vs Sham stimulation gait training (+/- virtual reality or treadmill)  20 minutes 1 -2 session/ week for 4 weeks | Uni-bilateral CP, GMFCS I-III, 7-18 yrs | 3D motion analysis | Objective: to investigate the effect of physical therapy interventions on spatiotemporal gait parameters in children with CP.  Vibratory platform, gait training, electrical stimulation, and transcranial stimulation were effective to improve spatiotemporal gait parameters, especially velocity in children with cerebral palsy. |
|  | 12.3 Novak et al. 2019 | SR: 4 SR | AMSTAR 2: moderate | tDCS | Spastic or dystonic CP, 4-19 yrs | / | tDCS (0.7-1 mA) + a motor learning rehabilitation (treadmill or VR) appeared to confer improved gait velocity, stride length, cadence and balance compared to sham tDCS + rehabilitation. Safe and well tolerated by children. Adverse effects are rare, mild, and transient and include minor tingling, burning, itching, and skin  redness. |
| 13. NMES | 13.1 Management Of Cerebral Palsy In Children: A Guide For Allied Health Professionals (NSW Ministry of Health guidelines, 2018) | CPG | AGREE II: high | NMES | Children and young persons with CP | / | pAG.79 FES is widely used in adult stroke populations but can be used for children with cerebral palsy. There is emerging evidence to support the use of FES for children with cerebral palsy in the lower limb and inconclusive evidence for its use in the upper limb |
|  | 13.2 Salazar et al. 2019 | SR: 6 RCT | AMSTAR 2: high | NMES +/- NDT vs treadmill or NDT or Conventional physical therapy  From 15 to 60 min, from 3 to 5/wk × 4-6 wk | Diplegic, hemiplegic, quadriplegic CP. Mean age 1.04-8.6 yrs | GMFM-88 or -66. | Objective: to investigate the effectiv of NMES as an adjuvant therapy to improve gross motor function in children with spastic CP Low-quality of evidence suggests that NMES might be used as adjuvant therapy to improve gross motor function in children with spastic CP, particularly the sitting and standing dimensions of GMFM scale. Our results need to be carefully interpreted due to the small number of studies included and the reduced sample size in each study. Further research with adequate methodological quality, ample sample size, and long-term follow-up are still necessary. |
|  | 13.3 Corsi et al. 2021 | SR: 5 RCT | AMSTAR 2: low | NMES | Uni-bilateral CP, GMFCS I-III, 7-18 yrs | cadence, gait velocity, and stride length | Vibratory platform, gait training, electrical stimulation, and transcranial stimulation were effective to improve spatiotemporal gait parameters, especially velocity in children with cerebral palsy. |
|  | 13.4 Das et al. 2019 | SR: 34 SR | AMSTAR 2: very low | NMES | 0-18 yrs, uni-bilateral CP | Muscle strength,  ROM,  and function | Objective: To evaluate the effectiveness of NMES on  gait or upper limb function in children with CP.  Electrical stimulation is associated with fewer functional gains |
|  | 13.5 Novak et al. 2019 | SR: 5 SR. | AMSTAR 2: moderate | NMES | Mixed CP types, GMFCS I-IV, 1-19 yrs | / | Controversial results about improving gait, low-level evidence about improving standing and sitting. |
| 14. NDT | 14.1 Management Of Cerebral Palsy In Children: A Guide For Allied Health Professionals (NSW Ministry of Health guidelines, 2018) | CPG | AGREE II: high | NDT | Children and young persons with CP | / | The child is a relatively passive recipient of the treatment and the approach is embedded into the context of normal developmental sequence. Novak et al. (2013) report a lack of evidence to support the use of NDT in current practice and indicate that alternative evidence based therapy interventions and approaches be used to provide more effective results. |
|  | 14.2 Zanon et al. 2019 | SR: 3 RCT. | AMSTAR 2: high | NDT vs  conventional  physical therapy  2-18 hr wk  for 6-16 wks | CP diplegia, GMFCS I-III, 3-15 yrs | GMFM–66 e 88, PEDI, PDMS-2, CAPE, MUUL, AHA, MACS, QUEST, wee FIM, Trunk control measurement  Scale, Pediatric Berg Balance Test, TUG, 1MWT, Manual muscle testing | Objective: to assess the effects of neurodevelopmental treatment for children with CP.  Further studies are required to assess the efficacy and safety of neurodevelopmental treatment for this purpose and until there, current evidence do not support its routinely use in practice. |
|  | 14.3 Das et al. 2019 | SR: 34 SR | AMSTAR 2: very low | NDT | 0-18 yrs, mostly hemiplegic CP |  | NDT has low-quality evidence |
|  | 14.4 Novak et al. 2019 | SR: 1 SR, 4 RCT. | AMSTAR 2: moderate | NDT | GMFCS I-III, 2-15 yrs |  | The effectiveness of neurodevelopmental treatment for children with CP is unclear. Because of the lack of good certainty evidence, neurodevelopmental treatment should be used with caution. The child’s response should be observed carefully. Recommendation downgraded to strong - |
| 15. HIPPOTHERAPY (HPT) | 15.1 Management Of Cerebral Palsy In Children: A Guide For Allied Health Professionals (NSW Ministry of Health guidelines, 2018) | CPG | AGREE II: high | Hippotherapy | Children and young persons with CP | / | PAG. 99 Horseback riding for therapy uses the horse’s movement which has an individual and variable gait, tempo, rhythm, repetition and cadence. It may influence neuromuscular development in humans. Improvements in trunk control and balance have been noted in children with cerebral palsy due to the physical adjustments to maintain proper alignment on the horse. From the current evidence it appears that hippotherapy and therapeutic horse riding have positive effects on balance and gross motor function in children with cerebral palsy although current literature and evidence is limited. |
|  | 15.2 De Guindos-Sanchez et al. 2020 | SR and meta-analysis: 10 RCT | AMSTAR 2: very low | HPT vs conventional  physical therapy intervention or placebo  30–45 min two times/week, for 8–12 week | mixed ages, GMFCS I-V. | GMFM, GMFM-66  KIDSCREEN-27  Questionnaire, MAS, CHQ, PEDI | Objective: to investigate on the effectiveness of hippotherapy to recover Gross Motor Function in Children with CP. Effective to improve gross motor function : GMFM-66 total scores and GMFM-88 dimensions A, B, and E. Furthermore, positive effects have been showed on balance recovery and muscle spasticity reduction. |
|  | 15.3 Novak et al. 2019 | SR: 5 SR, 3 RCT. | AMSTAR 2: moderate | 8 minutes to1  hour, 1-2/wk  for 8 weeks – 6  months | GMFCS I-V, uni-bilateral CP, 3-16 yrs |  | Conflicting findings in gross motor function. Hippotherapy conferred improved balance and  posture, specifically improving trunk position and arm function in GMFCS I-IV. |
|  | 15.4 Albuquerque de Araujo et al. 2019 | SR: 1 low-level study | AMSTAR 2: low | Hippotherapy + NDT VS NDT  30-45 min 7 times/week for 10 weeks. | mean age 7 yrs, uni-bilateral CP (missing data about GMFCS) | PBS, Cadence/walking speed | Objective: to investigate whether combining balance-training interventions with other active interventions enhances the effects of the active intervention alone on postural control of children and adolescents with CP. Very-low quality evidence suggests that BTI combined with other intervention enhances the effect of theother intervention alone on postural control at short-termin children and adolescents with CP. |
| 16. SUIT THERAPY | 16.1 Management Of Cerebral Palsy In Children: A Guide For Allied Health Professionals (NSW Ministry of Health guidelines, 2018) | CPG | AGREE II: high | Suit therapy | Children and young persons with CP | / | PAG. 97 Currently there is conflicting and limited evidence on the benefits of suit therapy. Some studies have shown no improvement in motor function while other studies have shown some benefit, including improved gait parameters. However, further investigation with larger sample sizes is recommended in the literature to determine the benefits of this intervention. |
|  | 16.2 Novak et al. 2019 | SR: 3 SR. | AMSTAR 2: moderate | suit therapy vs NDT  2-12 hrs/day  for 3-12 wks | CP mixed type, 3-17 yrs | GMFM | The suit might act on hip and shoulder stability and movement, given the suit is located over the hips and shoulders, whereas there was no effect on distal kinematics as the suit could not act on regions of the body not covered by the suit. Some children disliked wearing the suits and experienced adverse events including respiratory compromise, overheating and peripheral cyanosis. The suits also impeded function such as independent toileting and dressing. |
|  | 16.3 Karadag-Saygi et al. 2019 | SR: 29 studies (9 RCT). | AMSTAR 2: low | Suit therapies+ NTD vs NDT / Lycra functional splint+goal-directed training vs Goal-directed training / TheraSuit with the elastic bungee cords attached vs ‘‘Controlsuit’’ (TheraSuit without the elastic bungee cords) / Theratogs + solid GRAFO vs Traditional PT  2- 12 hours/day, 5 days/week for 4 weeks- 3 months | Children (<18 years) with a diagnosis  of CP  heterogenous | GMFM-66 and -88,, Energy cost during stair-climbing, GAS, MUUL, TUG, PEDI, PBS, SAS, Cobb angle, Kyphotic angle, Migration Index, Respiratory function, Grip strength, Abdominal strength, PODCI, BOTMP, COPM, QUEST,  Parent questionnaire, 10 MWT, PSFS, FAPS, VAS | Objective: to evaluate the clinical aspects and effectiveness of suit therapy for patients with CP.  Studies were heterogenous in design, type of suit, size, study population, and outcomes measured. Some improvement reported in proximal stability, gross motor function, but low evidence and several adverse effects. |
| 17. TAPING | 17.1 Inamdar et al. 2021 | SR and meta-analysis: 12 RCT. | AMSTAR 2: high | NDT + kinesio-taping vs Kinesio-taping | Uni-bilateral CP, GMFCS I-V, 18 mo-puberty | GMFM-sitting | Components of impairment remediation combined with functional balance training should be explored to improve sitting in children diagnosed with CP. Kinesio-taping may be an effective adjunct to conventional physical therapy in improving sitting ability in children with spastic bilateral CP.  Given the benefits of early achievement of sitting, strong evidence-based research is needed. |
|  | 17.2 Novak et al. 2019 | SR: 7 SR | AMSTAR 2: moderate | NDT + kinesio-taping vs Kinesio-taping  1-4 days/wk,  3 days – 10  mo duration | Uni-bilateral CP, GMFCS I-V, <18 yrs | / | Taping should be considered an adjunct to therapy, not stand-alone intervention, to improve gross motor and upper limbs function. Found to be most beneficial with GMFCS I-II, i.e. children with better selective motor control. Children had more active movement at the upper limbs, when the tape was elasticised compared to rigid tape.  A small number of children will have a skin allergy to the tape, and allergy is a contraindication. |
| 18. ORTHOSES | 18.1 Management Of Cerebral Palsy In Children: A Guide For Allied Health Professionals (NSW Ministry of Health guidelines, 2018) | CPG | AGREE II: high | Orthoses | Children and young persons with CP | / | PAG. 78 Functional orthoses generally position joints in a biomechanically advantageous position to either enable or improve function. Examples may include:  • Ankle foot orthoses (AFOs) – a variety of AFOs are available with varying purposes  • Wrist extension orthoses  • Neoprene wrist and thumb orthoses.  Positional orthoses aim to maintain corrected anatomical alignment of the joint and maintain range of motion around that joint. This may be important for ease of care, to reduce the requirement for future orthopaedic surgery and in some cases to maintain healthy skin integrity. Examples of positional orthoses may include:  • Spinal braces  • Leg or elbow wrap arounds  • Hip abduction orthoses.  The prescription and manufacture of upper and lower limb orthoses is common practice with children with cerebral palsy. Evidence suggests splints may be of some benefit when provided in conjunction with other therapies, although further research regarding splinting and orthoses is needed. |
|  | 18.2 Betancourt et al. 2019 | SR: 3 RCT, 14 prospective cohort studies | AMSTAR 2: very low | different  types of AFOs +/- OT vs OT or no treatment  1-wk to 1 yr | Uni-bilateral CP, GMFCS I-IV, 3-18 yrs | Dorsiflexion Angle, Stride Length | Objective: to analyze stride length and dorsiflexion data comparing the effectiveness of “ankle-foot orthoses” with “barefoot or shoes only” on ambulatory children with CP.  Children with cerebral palsy using ankle-foot orthoses had improved stride length and dorsiflexion angle during gait in a pooled meta-analyses of cohort studies and clinical trials. |
| 19. SERIAL CASTING | 19.1 Management Of Cerebral Palsy In Children: A Guide For Allied Health Professionals (NSW Ministry of Health guidelines, 2018) | CPG | AGREE II: high | Seral casting | Children and young persons with CP | / | PAG. 78 Casting is a therapy intervention used to gain/restore muscle length and provide soft tissue elongation. Casting can be done as a one off or as a series of casts depending on the desired outcome and the child’s tolerance for the cast. Casting is indicated when soft tissue contracture is interfering with function or causing potential biomechanical misalignment. Casting is not indicated when there are bony changes occurring at a joint. Casting only provides a short-term stretch and is usually required to be repeated at regular intervals and is particularly effective following Botulinum Toxin injections. There is currently no evidence to support upper limb casting being used in isolation, that is, it should be used in conjunction with other treatments that are focused on the activity level of the ICF. |
|  | 19.2 Milne et al. 2020 | SR 25 studies (mixed type, mostly had poor methodological quality). | AMSTAR 2: low | Serial casting +/-  other therapies (BTX- A) | CP or brain injury in children ≤ 18 years | ankle dorsiflexion PROM  MAS, MTS, GMFM, functional gait parameters, OGS | Objective: To evalute the effects of serial casting on lower limb function for children with CP. Lower limb serial casting was found to be effective for: improving ankle dorsiflexion PROM in the immediate to short-term, decreasing hypertonicity measured by MAS in the short-term. Serial casting with or without BTX-A did not significantly affect gross motor capacity measured by GMFM. Serial casting with pharmacological intervention achieved significantly more dorsiflexion PROM than serial casting alone. |
| 20. MASSAGE | 20.1 Management Of Cerebral Palsy In Children: A Guide For Allied Health Professionals (NSW Ministry of Health guidelines, 2018) | CPG | AGREE II: high | Massage | Children and young persons with CP | / | PAG 96. Massage is considered one of a variety of complementary and alternative medicines. There are a wide variety of massage techniques from gentle effleurage to deep tissue massage or myofascial release. Use of massage may help relieve muscle pain and tightness with a flow on effect to improve gait, range of motion and/or balance. Massage  may be used to relax a child after a bath, before sleeping, or to prepare for a therapy session. Children and young adults with cerebral palsy may suffer from cramps and spasms, more than their non-cerebral palsy peers. There is little evidence on the benefits of massage in children with cerebral palsy even though it is often recommended for the  psychological and/or relaxation benefits due to changes seen in cortisol levels. |
|  | 20.2 Guchan et al. 2020 | SR: 11 studies (7 RCT). | AMSTAR 2: very low | Massage + traditional therapy vs traditional physiotherapy/parents massage/OT  duration ranged from 14 to 30 min;  frequency ranged from 2 to 7 week/days per week | CP mixed, 0-18 yrs  GMFCS I-V (missing data relative to GMFCS in some studies) | PGMS, MAS; GMFM | Objective: to determine the effects of massage on the rehabilitation of children with CP.  Massage as an adjunct to traditional therapies should be used to reduce muscle tone in spastic-type CP. |

*Legend: 10MWT, 10 Metres Walking Test; 1MFWT, 1 Minute Fast Walking Test; 3DMA, upper limb three-dimensional motion analysis; 6MWT, 6 Minutes Walking Test; ABILHAND-Kids, questionnaire assessing the manual performances in activities of daily life; ABILOCO-Kids, measure of locomotion ability for children with lower limb impairments; ADL, Activities of daily living; AHA, Assisting Hand Assessment; AIMS, Alberta Infant Motor Scale; AMPS, assessment of motor and process skills; AOT, Action Observation Therapy; Aquaa, child-adapted Activity Questionnaire for Adults and Adolescents; BBT: Box and Block test; BDTT, backward downhill treadmill training; BOTMP, Bruininks-Oseretsky Test of Motor Proficiency; BOTMP subtest 5:6, Bruininks-Oseretsky Test of Motor Proficiency subtest of touching a swinging ball; BoNT-A, botulinum neurotoxin A; BOT-2: Bruininks-Oseretsky Test of Motor Proficiency 2nd edition; BSID-MDI, Bayley Scales of Infant Development-Mental Developmental Index; BTI, balance-training interventions; CAP, Capacity Profile; CAPE, Children’s Assessment of Participation and Enjoyment; CB&M, Community Balance and Mobility scale; CFUS, Caregiver Functional Use Survey; COPM, Canadian Occupational Performance Measure; CHEQ, Child’s Hand-use Experience Questionnaire; CHQ, Child Health Questionnaire; CIMT, Constraint Induced Movement Therapy; COPCA, Coping with and Caring for Infants with Special Needs; COPM, Canadian Occupational Performance Measure; CPT, conventional physical therapy; DPA, dynamic positional analysis; EMD, estimated mean difference; EMG, electromyography; FAB, Fullerton Advanced Balance Scale; FAPS, Functional Ambulation Performance Score; FFRT, Functional forward reach test; FRT, Functional Reach Test; FSRT, functional sideways reach test; FTSTS, Five Times Sit To Stand test; GAS, Goal Attainment Scale; GMFM, Gross Motor Function Measure; GMFM-88, Gross Motor Function Measure-88; HABIT, Hand -Arm Bimanual Intensive Therapy; HICT, High-Intensity Circuit Training; IMP, Infant Motor Profile; IPT, instrumented Pegboard Test; JTHFT, Jebsen- Taylor Hand Function Test; K- DTVP-2, Korean-Developmental Test of Visual Perception; LSS, Level of Sitting Scale; LSU, Lateral Step Up; MAS, Modified Ashworth Scale; MA, Melbourne Assessment; M-ABC, Movement Assessment Battery for Children; MABC-2, Movement Assessment Battery for Children -2nd edition; Mc, muscle; MCVG, Motion-Controlled Video Games; MGA, Maximum Grip Aperture; MMT, Manual Muscle Test; Mob-Que28, 28 question mobility questionnaire; mSOT, Modified Sensory Organization Test; MTS, Modified Tardieu Scale; MUUL, Melbourne Assessment of Unilateral Upper Limb; NDT, neurodevelopmental therapy; NFDR, neurodevelopmental facilitation reaction; NIBS, noninvasive brain stimulation; NMES, neuromuscular electrical stimulation; NHPT, Nine-hole peg test; nMT, normalized Movement Time; nMU, normalized Movement Unit; NWT, Nintendo Wii therapy; OGS, Observational Gait Scale; OLST, One Leg Stance Test; OT, occupational therapy; PAFT, Pediatric Arm Function Test; PBM, Pediatric balance measurement; PBS, Pediatric Balance Scale; PBWSTT, partial body weight supported treadmill training; PCI, Physiological Cost Index; PDMS, Peabody Developmental Motor Scales; PDMS-2, Peabody Developmental Motor Scales, Second Edition; PEDI, Pediatric Evalutation of Disability Inventory; PEDI-CAT, Paediatric Evaluation of Disability Inventory Computer Adaptive Test; PedsQL CP, Pediatric Quality of Life inventory Cerebral Palsy; PGMS, Peabody Gross Motor Scale; PMAL, Paediatric motor activity log; PROM, passive range of motion; PMGA, percent time where MGA occurs; PMT, perceptual motor therapy; PODCI, Paediatric Outcomes Data Collection Instrument; POMS, Profile of Mood States; PRT, Paediatric Reach Test; PSFS, Patient Specific Functional Scale; PT, physiotherapy; PV, Peak Velocity; QUEST, Quality of Upper Extremity Skills Test; QUEST, Quebec User Evalutation of Satisfaction with Assistive Techonology; RM, Resistance Maximum; RMS, Root Mean Square; ROM, Range Of Motion; RS, Resistance Strengthening; RSE, Self-esteem questionairres; RT, Reaction Time; rTMS, repetitive transcranial magnetic stimulation; SAS, Sitting Assessment Scale; SCALE, Selective Control Assessment of the Lower Extremity; SCG, standard care group; SDR, Selective dorsal rhizotomy; SFA, school function assessment travel scale; SFA, spontaneous functional analysis; SHUEE, Shriners Hospital for Children Upper Extremity Evaluation; SPCC, Self-perception profile for children; SSTTEP, supported speed treadmill training exercise program; STS, Sit-To-Stand; TBWS, treadmill training with body weight support; tDCS, transcranial direct current stimulation; TEG, therapeutic exercise group; TGT, treadmill gait training; TIP, Traditional infant physical therapy; TST, Timed Stairs Test; TUDS, Timed Up and Down Stairs Test; TUG Time Up and Go test; UEU, Universal Exercise Unit; TVMS, Test of Visuo Motor Skills revised; TVPS, Test of Visual- Perceptual Skills; UE, upper extremity; VABS, Vineland Adaptive Behavior Scales; VAS, Visual Analog Scale; VMI, Visual Motor Integration Test; VOT, Video game Observation Treatment; VRT, virtual reality therapy; WB-SI, Weight Bearing-Simmetry Index; WeeFIM, Functional Independence Measure for children; WHODAS, World Health Organization Disability Assessment Schedule.*
