# Supplementary material for: Evidence-based management and motor rehabilitation of cerebral palsy children and adolescents: a systematic review
Source: Front Neurol. 2023 May 25;14:1171224. doi: 10.3389/fneur.2023.1171224 (PMC10248244; doi:10.3389/fneur.2023.1171224)
Supplement: Supplementary file 5 [file Data_Sheet_5.docx]

| **Supplementary digital material 5: supplementary table 6a.**  Evidence profile comparing any type of physioterapy, occupational therapy, neuromotor intervention to nothing or any other intervention for improving **gross motor function** in CP age 2-18 years (Ryan et al. 2017; Bania et al 2019; Zanon et al. 2019; Chiu et al 2020; Elbanna et al. 2019; Salazar et al. 2019; Armstrong et al. 2019; Abdelhaleem et al 2021; De Guindos-Sanchez et al. 2020; Hsu et al. 2019; Ren et al. 2019; Cobo-Vicente 2021). | | | | | | | | | | | |
| --- | --- | --- | --- | --- | --- | --- | --- | --- | --- | --- | --- |
| Certainty assessment | | | | | | | *Summary of findings* | | | | |
| Participants (studies) Follow-up | *Risk of bias* | *Inconsistency* | *Indirectness* | *Imprecision* | *Publication bias* | *Overall certainty of evidence* | *Study event rates (%)* | | *Relative effect (95% CI)* | *Anticipated absolute effects* | |
|  |  |  |  |  |  |  | *With nothing or any other intervention* | *With any type of physioterapy, occupational therapy, neuromotor intervention* |  | *Risk with nothing or any other intervention* | *Risk difference with any type of physiotherapy, occupational therapy, neuromotor intervention* |
| AEROBIC TRAINING versus USUAL CARE short term (Ryan 2017) (follow-up: range 2 weeks to 4 weeks; assessed with: GMFM; Scale from: 0 to 100) | | | | | | | | | | | |
| 65 (3 RCTs) | not serious | not serious | not serious | not serious | none | ⨁⨁⨁⨁ High | 28 | 37 | - | The mean AEROBIC TRAINING versus USUAL CARE short term (Ryan 2017) was 0 % | mean 0.53 % higher (0.02 higher to 1.04 higher) |
| AEROBIC TRAINING versus USUAL CARE intermediate term (Ryan 2017) (follow-up: mean 6 weeks; assessed with: GMFM; Scale from: 0 to 100) | | | | | | | | | | | |
| 12 (1 RCT) | not serious | not serious | not serious | very serious^a^ | none | ⨁⨁◯◯ Low | 6 | 6 | - | The mean AEROBIC TRAINING versus USUAL CARE intermediate term (Ryan 2017) was 0 % | mean 12.96 % higher (0.52 higher to 25.4 higher) |
| RESISTANCE TRAINING versus USUAL CARE short term (Ryan 2017) (follow-up: mean 4 weeks; assessed with: GMFM; Scale from: 0 to 100) | | | | | | | | | | | |
| 164 (7 RCTs) | not serious | not serious | not serious | not serious | none | ⨁⨁⨁⨁ High | 77 | 87 | - | The mean RESISTANCE TRAINING versus USUAL CARE short term (Ryan 2017) was 0 % | mean 0.12 % higher (0.19 lower to 0.43 higher) |
| RESISTANCE TRAINING versus USUAL CARE intermediate term (Ryan 2017) (follow-up: range 4 weeks to 6 months; assessed with: GMFM) | | | | | | | | | | | |
| 85 (3 RCTs) | not serious | not serious | not serious | not serious | none | ⨁⨁⨁⨁ High | 41 | 44 | - | The mean RESISTANCE TRAINING versus USUAL CARE intermediate term (Ryan 2017) was 0 % | mean 0.13 % higher (0.3 lower to 0.55 higher) |
| RESISTANCE TRAINING versus USUAL CARE (Cho 2020) (follow-up: mean 6 weeks; assessed with: GMFM; Scale from: 0 to 100) | | | | | | | | | | | |
| 25 (1 RCT) | not serious | not serious | not serious | serious^a^ | none | ⨁⨁⨁◯ Moderate | 12 | 13 | - | The mean RESISTANCE TRAINING versus USUAL CARE (Cho 2020) was 0 % | mean 1.3 % lower (1.284 lower to 0.26 higher) |
| MIXED TRAINING versus USUAL CARE short term (Ryan 2017) (follow-up: range 2 weeks to 4 weeks; assessed with: GMFM; Scale from: 0 to 100) | | | | | | | | | | | |
| 163 (4 RCTs) | not serious | not serious | not serious | not serious | none | ⨁⨁⨁⨁ High | 82 | 81 | - | The mean MIXED TRAINING versus USUAL CARE short term (Ryan 2017) was 0 % | mean 0.02 % higher (0.29 lower to 0.33 higher) |
| RESISTANCE TRAINING versus AEROBIC TRAINING short term (Ryan 2017) (follow-up: range 2 weeks to 4 weeks; assessed with: GMFM; Scale from: 0 to 100) | | | | | | | | | | | |
| 56 (2 RCTs) | not serious | not serious | not serious | serious^a,b^ | none | ⨁⨁⨁◯ Moderate | 29 | 27 | - | The mean RESISTANCE TRAINING versus AEROBIC TRAINING short term (Ryan 2017) was 0 % | mean 0.02 % higher (0.5 lower to 0.55 higher) |
| ACTIVITY ON THE GROUND (walking, stairs climbing, balance, sit-to-stand...) versus no intervention (Bania 2019) (assessed with: GMFM; Scale from: 0 to 100) | | | | | | | | | | | |
| 61 (2 RCTs) | very serious^b^ | very serious^c^ | not serious | serious^a^ | publication bias strongly suspected^b^ | ⨁◯◯◯ Very low | 30 | 31 | - | The mean ACTIVITY ON THE GROUND (walking, stairs climbing, balance, sit-to-stand...) versus no intervention (Bania 2019) was 0 % | mean 0.18 % higher (1.49 lower to 1.86 higher) |
| ACTIVITY ON THE GROUND versus OTHER TREATMENT (Bania 2019) (assessed with: GMFM; Scale from: 0 to 100) | | | | | | | | | | | |
| 156 (5 RCTs) | very serious^b^ | serious^c^ | not serious | not serious | publication bias strongly suspected^b^ | ⨁◯◯◯ Very low | 78 | 78 | - | The mean ACTIVITY ON THE GROUND versus OTHER TREATMENT (Bania 2019) was 0 % | mean 0.09 % lower (0.86 lower to 0.69 higher) |
| CONVENTIONAL THERAPY versus NDT (Bobath) (Zanon 2019: Arai 2014) (follow-up: mean 24 weeks; assessed with: GMFM-88; Scale from: 0 to 100) | | | | | | | | | | | |
| 16 (1 RCT) | not serious | very serious^c^ | not serious | very serious^a^ | publication bias strongly suspected^b^ | ⨁◯◯◯ Very low | 8 | 8 | - | The mean CONVENTIONAL THERAPY versus NDT (Bobath) (Zanon 2019: Arai 2014) was 0 % | 5.19 % higher (10 lower to 20.39 higher) |
| MECHANICALLY ASSISTED WALKING WITHOUT WEIGHT SUPPORT versus NO WALKING (Chiu 2020) (follow-up: mean 7 weeks; assessed with: GMFM-E %; Scale from: 0 to 100) | | | | | | | | | | | |
| 60 (2 RCTs) | not serious | not serious | not serious | not serious | none | ⨁⨁⨁⨁ High | 30 | 30 | - | The mean MECHANICALLY ASSISTED WALKING WITHOUT WEIGHT SUPPORT versus NO WALKING (Chiu 2020) was 0 % | 1.3 % higher (0.49 higher to 2.11 higher) |
| MECHANICALLY ASSISTED WALKING WHITOUT BODY SUPPORT versus SAME DOSE OF OVERGROUND WALKING (Chiu 2020) (follow-up: mean 12 weeks; assessed with: GMFM-E %; Scale from: 0 to 100) | | | | | | | | | | | |
| 35 (1 RCT) | not serious | not serious | not serious | very serious^a^ | none | ⨁⨁◯◯ Low | 18 | 17 | - | The mean MECHANICALLY ASSISTED WALKING WHITOUT BODY SUPPORT versus SAME DOSE OF OVERGROUND WALKING (Chiu 2020) was 0 % | 11.9 % higher (2.98 higher to 20.82 higher) |
| MECHANICAL ASSISTED WALKING WITH BODY WEIGHT SUPPORT versus no WALKING (Chiu 2020) (follow-up: range 4 weeks to 12 weeks; assessed with: GMFM-E %; Scale from: 0 to 100) | | | | | | | | | | | |
| 58 (3 RCTs) | not serious | not serious | not serious | very serious^a,d^ | none | ⨁⨁◯◯ Low | 31 | 27 | - | The mean MECHANICAL ASSISTED WALKING WITH BODY WEIGHT SUPPORT versus no WALKING (Chiu 2020) was 0 % | 1.09 % higher (0.57 lower to 2.75 higher) |
| MECHANICALLY ASSISTED WALKING WITH BODY WEIGHT SUPPORT versus SAME DOSE OVERGROUND WALKING (Chiu 2020) (follow-up: range 8 weeks to 10 weeks; assessed with: GMFM-E %; Scale from: 0 to 100) | | | | | | | | | | | |
| 52 (2 RCTs) | not serious | serious^d^ | not serious | serious^a,d^ | none | ⨁⨁◯◯ Low | 25 | 27 | - | The mean MECHANICALLY ASSISTED WALKING WITH BODY WEIGHT SUPPORT versus SAME DOSE OVERGROUND WALKING (Chiu 2020) was 0 % | 0.73 % lower (14.38 lower to 12.92 higher) |
| NMES versus CONVENTIONAL THERAPY (Salazar 2019) (assessed with: GMFM-D E; Scale from: 0 to 100) | | | | | | | | | | | |
| 226 (8 RCTs) | not serious | serious^a,c^ | not serious | serious^a,d^ | none | ⨁⨁◯◯ Low | 110 | 116 | - | The mean NMES versus CONVENTIONAL THERAPY (Salazar 2019) was 0 % | 3.58 % higher (1.47 higher to 5.69 higher) |
| tDCS (transcranial Direct Current Stimulation) + VR or treadmill versus PLACEBO + VR or treadmill (Elbanna 2019) (follow-up: 1 months; assessed with: GMFM D; Scale from: 0 to 100) | | | | | | | | | | | |
| 48 (2 RCTs) | serious^b^ | serious^d^ | not serious | serious^a,d^ | none | ⨁◯◯◯ Very low | 24 | 24 | - | The mean tDCS (transcranial Direct Current Stimulation) + VR or treadmill versus PLACEBO + VR or treadmill (Elbanna 2019) was 0 % | 0.74 % higher (0.14 higher to 1.34 higher) |
| tDCS + VR or treadmill versus PLACEBO + VR or treadmill (Elbanna 2019) (follow-up: 1 months; assessed with: GMFM-E; Scale from: 0 to 100) | | | | | | | | | | | |
| 48 (2 RCTs) | serious^b^ | serious^d^ | not serious | serious^a,d^ | none | ⨁◯◯◯ Very low | 24 | 24 | - | The mean tDCS + VR or treadmill versus PLACEBO + VR or treadmill (ELBANNA 2019) was 0 % | 0.57 % higher (0.02 lower to 1.17 higher) |
| CYCLING versus TREADMILL OR CONVENTIONAL PHYSIOTHERAPY (Armstrong 2019) (assessed with: GMFM; Scale from: 0 to 100) | | | | | | | | | | | |
| 107 (3 RCTs) | not serious | serious^d^ | not serious | serious^a,d^ | none | ⨁⨁◯◯ Low | 54 | 53 | - | The mean CYCLING versus treadmill or conventional physiotherapy (Armstrong 2019) was 0 % | 0.29 % higher (0.09 lower to 0.68 higher) |
| AOT versus CONVENTIONAL PHYSIOTHERAPY (Abdelhaleem 2021) (assessed with: GMFM; Scale from: 0 to 100) | | | | | | | | | | | |
| 48 (2 RCTs) | not serious | very serious^a,c,d^ | not serious | very serious^a,d,e^ | none | ⨁◯◯◯ Very low | 24 | 24 | - | The mean AOT versus CONVENTIONAL PHYSIOTHERAPY (Abdelhaleem 2021) was 0 % | 1.96 % higher (0.84 lower to 4.76 higher) |
| VR (Wii platform, virtual bicycle, etc) versus CONVENTIONAL PHYSIOTHERAPY (Ren 2019): DATI DEMOGRAFICI DI ALCUNI STUDI INCOMPLETI (assessed with: GMFM D-E; Scale from: 0 to 100) | | | | | | | | | | | |
| 459 (9 RCTs) | serious^b,c,d,e^ | serious^d,e^ | not serious | serious^d,e^ | none | ⨁◯◯◯ Very low | 229 | 230 | - | The mean VR (Wii platform, virtual bicycle, etc) versus conventional physiotherapy (Ren 2019): DATI DEMOGRAFICI DI ALCUNI STUDI INCOMPLETI was 0 % | 0.23 % higher (0.04 higher to 0.41 higher) |
| IPPOTERAPIA versus NO TREATMENT O AEROBIC TRAINING (De Guindos-Sanchez 2020) (follow-up: 8 weeks; assessed with: GMFM; Scale from: 0 to 100) | | | | | | | | | | | |
| 146 (2 RCTs) | very serious^b,d^ | serious^c,d^ | not serious | serious^c,d^ | none | ⨁◯◯◯ Very low | 67 | 79 | - | The mean IPPOTERAPIA versus NO TREATMENT O AEROBIC TRAINING (De Guindos-Sanchez 2020) was 0 % | 0.6 % higher (0.11 lower to 1.31 higher) |
| INTENSIVE (>x3/week) OR USUAL PHYSIOTHERAPY versus baseline (Hsu 2019) (assessed with: GMFM 66 e 88; Scale from: 0 to 100) | | | | | | | | | | | |
| 229 (6 RCTs) | serious^b,e^ | not serious | not serious | serious^d^ | none | ⨁⨁◯◯ Low | 114 | 115 | - | The mean INTENSIVE (>X3/WEEK) OR USUAL PHYSIOTHERAPY versus baseline (Hsu 2019) was 0 % | 2.093 % higher (1.657 higher to 2.53 higher) |
| INTENSIVE versus USUAL PHYSIOTHERAPY (Hsu 2019) (assessed with: GMFM-66; Scale from: 0 to 100) | | | | | | | | | | | |
| 312 (9 RCTs) | serious^b,e^ | not serious | not serious | serious^c,d^ | none | ⨁⨁◯◯ Low | 156 | 156 | - | The mean INTENSIVE versus USUAL PHYSIOTHERAPY (Hsu 2019) was 0 % | 0.11 % higher (0.138 lower to 0.359 higher) |
| NMES + fkt versus fkt (Cobo-Vicente 2021) (assessed with: GMFM) | | | | | | | | | | | |
| 362 (9 RCTs) | serious^f^ | not serious | not serious | serious^d^ | none | ⨁⨁◯◯ Low | 180 | 182 | - | The mean NMES + fkt versus fkt (Cobo-Vicente 2021) was 0 | mean 0.41 higher (0.19 higher to 0.64 higher) |

#### *Legend: RCT, randomized controlled trial; CI, confidence interval; ^a^ small sample; ^b^ lacking description of review protocol,risk of bias analysis, funding of studies; ^c^ conflicting results among studies; ^d^ large CI; ^e^ the meta-analysis repeatedly consider data concerning different GMFM dimensions of the same subjects; ^f^ moderate quality based on AMSTAR.*

| **Supplementary digital material 5: supplementary table 6b.**  Evidence profile comparing any type of physioterapy, occupational therapy, neuromotor intervention, to nothing or any other intervention for improving **balance** in CP age 2-18 years (Merino-Andres et al. 2021; Han et al. 2020; Elbanna et al. 2019; Montoro-Cardenas et al. 2021; Warnier et al. 2019; Wu et al. 2021). | | | | | | | | | | | |
| --- | --- | --- | --- | --- | --- | --- | --- | --- | --- | --- | --- |
| Certainty assessment | | | | | | | *Summary of findings* | | | | |
| Participants (studies) Follow-up | *Risk of bias* | *Inconsistency* | *Indirectness* | *Imprecision* | *Publication bias* | *Overall certainty of evidence* | *Study event rates (%)* | | *Relative effect (95% CI)* | *Anticipated absolute effects* | |
|  |  |  |  |  |  |  | *With nothing or any other intervention* | *With any type of physioterapy, occupational therapy, neuromotor intervention* |  | *Risk with nothing or any other intervention* | *Risk difference with any type of physiotherapy, occupational therapy, neuromotor intervention* |
| RESISTANCE (STRENGHT) TRAINING versus OTHER TREATMENT (Merino-Andres 2021) (assessed with: BERG BALANCE SCALE; Scale from: 0 to 56) | | | | | | | | | | | |
| 280 (8 RCTs) | not serious | serious^a,b^ | not serious | serious^a,b^ | none | ⨁⨁◯◯ Low | 139 | 141 | - | The mean RESISTANCE (STRENGHT) TRAINING versus OTHER TREATMENT (Merino-Andres 2021) was 0 | 0.784 lower (1.029 lower to 0.54 lower) |
| TREADMILL versus OTHER INTERVENTION (Han 2020) (assessed with: SECONDS) | | | | | | | | | | | |
| 44 (3 RCTs) | serious^c^ | serious^a^ | not serious | serious^a^ | none | ⨁◯◯◯ Very low | 22 | 22 | - | The mean TREADMILL versus OTHER INTERVENTION (Han 2020) was 0 sec | 0.738 sec higher (0.138 higher to 1.339 higher) |
| TMS + VR o treadmill versus PLACEBO + VR o treadmill COP-AP-eye open (Elbanna 2019) (follow-up: mean 1 months; assessed with: mm) | | | | | | | | | | | |
| 48 (3 RCTs) | very serious^d^ | serious^b^ | not serious | serious^b^ | none | ⨁◯◯◯ Very low | 24 | 24 | - | The mean TMS + VR o treadmill versus PLACEBO + VR o treadmill COP-AP-eye open (Elbanna 2019) was 0 mm | 0.48 mm higher (0.99 lower to 0.04 higher) |
| TMS + VR o treadmill versus PLACEBO + VR o treadmill COP-ML-eye open (Elbanna 2019) (follow-up: mean 1 months; assessed with: mm) | | | | | | | | | | | |
| 48 (3 RCTs) | very serious^d^ | serious^a,b^ | not serious | serious^a,b^ | none | ⨁◯◯◯ Very low | 24 | 24 | - | The mean TMS + VR o treadmill versus PLACEBO + VR o treadmill COP-ML-eye open (Elbanna 2019) was 0 mm | 0.28 mm higher (0.23 lower to 0.79 higher) |
| TMS + VR o treadmill versus PLACEBO + VR o treadmill COP-AP-eye closed (Elbanna 2019) (follow-up: mean 1 months; assessed with: mm) | | | | | | | | | | | |
| 48 (3 RCTs) | very serious^d^ | serious^a,b^ | not serious | serious^a,b^ | none | ⨁◯◯◯ Very low | 24 | 24 | - | The mean TMS + VR o treadmill versus PLACEBO + VR o treadmill COP-AP-eye closed (Elbanna 2019) was 0 mm | 0.7 mm higher (0.17 higher to 1.21 higher) |
| tDCS + VR o treadmill versus PLACEBO + VR o treadmill COP-ML-eye closed (Elbanna 2019) (follow-up: mean 1 months; assessed with: mm) | | | | | | | | | | | |
| 48 (3 RCTs) | very serious^d^ | serious^a,b^ | not serious | very serious^a,b^ | none | ⨁◯◯◯ Very low | 24 | 24 | - | The mean tDCS + VR o treadmill versus PLACEBO + VR o treadmill COP-ML-eye closed (Elbanna 2019) was 0 mm | 0.42 mm higher (0.11 lower to 0.95 higher) |
| Wii (alone or + physiotherapy) versus CONVENTIONAL PHYSIOTHERAPY or NO THERAPY (Montoro-Cardenas 2021) (assessed with: PBS; Scale from: 0 to 56) | | | | | | | | | | | |
| 281 (8 RCTs) | not serious | serious^b^ | not serious | serious^a,b^ | none | ⨁⨁◯◯ Low | 187 | 94 | - | The mean wii (alone or + physiotherapy) versus CONVENTIONAL PHYSIOTHERAPY or NO THERAPY (Montoro-Cardenas 2021) was 0 | 0.833 higher (0.382 higher to 1.284 higher) |
| VR videogames (Wii, Xbox, ecc.) versus NO VR TREATMENT (Warnier 2019) (assessed with: mixed outcome measures) | | | | | | | | | | | |
| 140 (5 RCTs) | serious^c^ | serious^a,b,e^ | not serious | serious^a,b,e^ | none | ⨁◯◯◯ Very low | 71 | 69 | - | The mean VR videogames (Wii, Xbox, ecc.) versus NO VR TREATMENT (Warnier 2019) was 0 | 0.89 higher (0.14 higher to 1.63 higher) |
| VR videogames (Wii, Xbox, etc) alone or without conventional physiotherapy versus CONVENTIONAL PHYSIOTERAPHY (Wu 2021) (assessed with: mixed outcome measures) | | | | | | | | | | | |
| 348 (11 RCTs) | very serious^d^ | serious^a,e^ | not serious | serious^a,e^ | none | ⨁◯◯◯ Very low | 172 | 176 | - | The mean VR videogames (Wii, Xbox, etc) da soli o con conventional physiotherapy versus CONVENTIONAL PHYSIOTERAPHY (Wu 2021) was 0 | 0.29 higher (0.1 higher to 0.48 higher) |

#### *Legend: RCT, randomized controlled trial; CI, confidence interval; ^a^ large standard deviation; ^b^ Conflicting results among studies; ^c^ moderate quality based on AMSTAR; ^d^ low quality based on AMSTAR; ^e^ The authors mix different outcome measures and repeatedly consider the same study into the meta-analysis*.

| **Supplementary digital material 5: supplementary table 6c.**  Evidence profile comparing any type of physioterapy, occupational therapy, neuromotor intervention, to nothing or any other intervention for improving **gait** in CP age 2-18 years (Ryan et al. 2017; Han et al. 2020; Warnier et al.2019; Chiu et al. 2020). | | | | | | | | | | | |
| --- | --- | --- | --- | --- | --- | --- | --- | --- | --- | --- | --- |
| Certainty assessment | | | | | | | *Summary of findings* | | | | |
| Participants (studies) Follow-up | *Risk of bias* | *Inconsistency* | *Indirectness* | *Imprecision* | *Publication bias* | *Overall certainty of evidence* | *Study event rates (%)* | | *Relative effect (95% CI)* | *Anticipated absolute effects* | |
|  |  |  |  |  |  |  | *With nothing or any other intervention* | *With any type of physioterapy, occupational therapy, neuromotor intervention* |  | *Risk with nothing or any other intervention* | *Risk difference with any type of physiotherapy, occupational therapy, neuromotor intervention* |
| AEROBIC versus USUAL CARE short term (Ryan 2017) (follow-up: range 2 weeks to 4 weeks; assessed with: METERS/SECOND) | | | | | | | | | | | |
| 82 (4 RCTs) | not serious | not serious | not serious | serious^a^ | none | ⨁⨁⨁◯ Moderate | 41 | 41 | - | The mean AEROBIC versus USUAL CARE short term (Ryan 2017) was 0 m/s | 0.09 m/s higher (0.11 lower to 0.28 higher) |
| AEROBIC versus USUAL CARE intermediate term (Ryan 2017) (follow-up: mean 6 weeks; assessed with: METERS/SECOND) | | | | | | | | | | | |
| 12 (1 RCT) | not serious | not serious | not serious | very serious^b^ | none | ⨁⨁◯◯ Low | 6 | 6 | - | The mean AEROBIC versus USUAL CARE intermediate term (Ryan 2017) was 0 m/s | 0.17 m/s lower (0.59 lower to 0.24 higher) |
| RESISTANCE TRAINING versus USUAL CARE short term (Ryan 2017) (follow-up: mean 4 weeks; assessed with: METERS/SECOND) | | | | | | | | | | | |
| 185 (8 RCTs) | not serious | not serious | not serious | serious^a^ | none | ⨁⨁⨁◯ Moderate | 82 | 103 | - | The mean RESISTANCE TRAINING versus USUAL CARE short term (Ryan 2017) was 0 m/s | 0.03 m/s higher (0.02 lower to 0.07 higher) |
| RESISTANCE TRAINING versus USUAL CARE intermediate term (Ryan 2017) (assessed with: METERS/SECOND) | | | | | | | | | | | |
| 84 (3 RCTs) | not serious | not serious | not serious | serious^a^ | none | ⨁⨁⨁◯ Moderate | 40 | 44 | - | The mean RESISTANCE TRAINING versus USUAL CARE intermediate term (Ryan 2017) was 0 m/s | 0.03 m/s lower (0.17 lower to 0.11 higher) |
| MIXED TRAINING versus USUAL CARE short term (Ryan 2017) (assessed with: METERS/SECOND) | | | | | | | | | | | |
| 58 (1 RCT) | not serious | serious^a,b^ | not serious | very serious^a,b^ | none | ⨁◯◯◯ Very low | 29 | 29 | - | The mean MIXED TRAINING versus USUAL CARE short term (Ryan 2017) was 0 m/s | 0.1 m/s higher (0.07 lower to 0.27 higher) |
| RESISTANCE TRAINING versus AEROBIC TRAINING short term (Ryan 2017) (assessed with: METERS/SECOND) | | | | | | | | | | | |
| 26 (1 RCT) | not serious | serious^a,b^ | not serious | very serious^a,b^ | none | ⨁◯◯◯ Very low | 12 | 14 | - | The mean RESISTANCE TRAINING versus AEROBIC TRAINING short term (Ryan 2017) was 0 m/s | 0.12 m/s higher (0.15 lower to 0.39 higher) |
| RESISTANCE TRAINING versus AEROBIC TRAINING intermediate term (Ryan 2017) (assessed with: METERS/SECOND) | | | | | | | | | | | |
| 26 (1 RCT) | not serious | serious^a,b^ | not serious | serious^a,b^ | none | ⨁⨁◯◯ Low | 12 | 14 | - | The mean RESISTANCE TRAINING versus AEROBIC TRAINING intermediate term (Ryan 2017) was 0 m/s | 0.19 m/s higher (0.05 lower to 0.43 higher) |
| TREADMILL versus OTHER INTERVENTION (strenghtening, stretching, conventional physiotherapy, overground walking) (Han 2020) (assessed with: METERS/SECOND) | | | | | | | | | | | |
| 179 (8 RCTs) | serious^c^ | serious^a^ | not serious | serious^a^ | none | ⨁◯◯◯ Very low | 90 | 89 | - | The mean TREADMILL versus OTHER INTERVENTION (strenghtening, stretching, conventional physiotherapy, overground walking) (Han 2020) was 0 m/s | 0.532 m/s higher (0.146 higher to 0.918 higher) |
| VR versus NO VR THERAPY (Warnier 2019) (assessed with: METERS) | | | | | | | | | | | |
| 108 (5 RCTs) | serious^c^ | serious^a,d^ | not serious | serious^a,d^ | none | ⨁◯◯◯ Very low | 54 | 54 | - | The mean VR versus NO VR THERAPY (Warnier 2019) was 0 METRI | 3.1 METRI higher (0.78 higher to 5.43 higher) |
| MECHANICALLY ASSISTED WALKING WITHOUT BODY WEIGHT SUPPORT versus NO WALKING (Chiu 2020) (assessed with: METERS/SECOND) | | | | | | | | | | | |
| 10 (1 RCT) | not serious | very serious^b,e^ | not serious | very serious^b^ | none | ⨁◯◯◯ Very low | 5 | 5 | - | The mean MECHANICALLY ASSISTED WALKING WITHOUT BODY WEIGHT SUPPORT versus NO WALKING (Chiu 2020) was 0 m/s | 0.05 m/s higher (0.03 higher to 0.07 higher) |
| MECHANICALLY ASSISTED WALKING WITHOUT BODY WEIGHT SUPPORT versus SAME DOSE OVERGROUND WALKING (Chiu 2020) (follow-up: range 7 weeks to 12 weeks; assessed with: METERS/SECOND) | | | | | | | | | | | |
| 55 (2 RCTs) | not serious | serious^b^ | not serious | serious^b,f^ | none | ⨁⨁◯◯ Low | 28 | 27 | - | The mean MECHANICALLY ASSISTED WALKING WITHOUT BODY WEIGHT SUPPORT versus SAME DOSE OVERGROUND WALKING (Chiu 2020) was 0 m/s o min | 0.25 m/s o min higher (0.13 higher to 0.37 higher) |
| MECHANICALLY ASSISTED WALKING WITH BODY WEIGHT SUPPORT versus NO WALKING (Chiu 2020) (assessed with: METERS/SECOND) | | | | | | | | | | | |
| 161 (7 RCTs) | not serious | serious^a^ | not serious | serious^a^ | none | ⨁⨁◯◯ Low | 72 | 89 | - | The mean MECHANICALLY ASSISTED WALKING WITH BODY WEIGHT SUPPORT versus NO WALKING (Chiu 2020) was 0 m/s | 0.07 m/s higher (0.06 higher to 0.08 higher) |
| MECHANICALLY ASSISTED WALKING WITH BODY WEIGHT SUPPORT versus SAME DOSE OVERGROUND WALKING (Chiu 2020) (assessed with: METERS/SECOND) | | | | | | | | | | | |
| 78 (3 RCTs) | not serious | not serious | not serious | serious^a,b^ | none | ⨁⨁⨁◯ Moderate | 39 | 39 | - | The mean MECHANICALLY ASSISTED WALKING WITH BODY WEIGHT SUPPORT versus SAME DOSE OVERGROUND WALKING (Chiu 2020) was 0 m/s | 0.02 m/s lower (0.08 lower to 0.04 higher) |

#### *Legend: RCT, randomized controlled trial; CI, confidence interval; ^a^ large standard deviation; ^b^ small sample; ^c^ moderate quality based on AMSTAR; ^d^ conflicting results among studies; ^e^ only one study included; ^f^ the authors mix two different outcome measures*

| **Supplementary digital material 5: supplementary table 6d.**  Evidence profile comparing any type of physioterapy, occupational therapy, neuromotor intervention, to nothing or any other intervention for improving **manual function and activities** in CP age 2-18 years (Abdelhaleem et al. 2021; Johansen et al. 2020; Elbanna et al. 2019). | | | | | | | | | | | |
| --- | --- | --- | --- | --- | --- | --- | --- | --- | --- | --- | --- |
| Certainty assessment | | | | | | | *Summary of findings* | | | | |
| Participants (studies) Follow-up | *Risk of bias* | *Inconsistency* | *Indirectness* | *Imprecision* | *Publication bias* | *Overall certainty of evidence* | *Study event rates (%)* | | *Relative effect (95% CI)* | *Anticipated absolute effects* | |
|  |  |  |  |  |  |  | *With nothing or any other intervention* | *With any type of physioterapy, occupational therapy, neuromotor intervention* |  | *Risk with nothing or any other intervention* | *Risk difference with any type of physiotherapy, occupational therapy, neuromotor intervention* |
| AOT versus video without motor content+ usual physiotherapy (Abdelhaleem 2021) IMMEDIATE POST INTERVENTION (assessed with: AHA) | | | | | | | | | | | |
| 108 (4 RCTs) | not serious | serious^a,b^ | not serious | serious^a,b^ | none | ⨁⨁◯◯ Low | 53 | 55 | - | The mean AOT versus video without motor content+ usual physiotherapy (Abdelhaleem 2021) IMMEDIATE POST INTERVENTION was 0 | 0.1 higher (0.29 lower to 0.48 higher) |
| AOT versus video without motor content+ usual physiotherapy (Abdelhaleem 2021) INTERMEDIATE TERM (follow-up: range 8 weeks to 24 weeks; assessed with: AHA) | | | | | | | | | | | |
| 95 (3 RCTs) | not serious | serious^a^ | not serious | not serious | none | ⨁⨁⨁◯ Moderate | 46 | 49 | - | The mean AOT versus video without motor content+ usual physiotherapy (Abdelhaleem 2021) INTERMEDIATE TERM was 0 | 0.01 higher (0.4 lower to 0.41 higher) |
| AOT versus video without motor content+ usual physiotherapy (Abdelhaleem 2021) IMMEDIATE POST INTERVENTION (assessed with: MUUL) | | | | | | | | | | | |
| 65 (4 RCTs) | not serious | serious^a,b^ | not serious | serious^a,b^ | none | ⨁⨁◯◯ Low | 30 | 35 | - | The mean AOT versus video without motor content+ usual physiotherapy (Abdelhaleem 2021) IMMEDIATE POST INTERVENTION was 0 | 0.29 higher (0.64 lower to 1.23 higher) |
| AOT versus video without motor content+ usual physiotherapy (Abdelhaleem 2021) INTERMEDIATE TERM (follow-up: range 8 weeks to 24 weeks; assessed with: MUUL) | | | | | | | | | | | |
| 42 (2 RCTs) | not serious | not serious | not serious | serious^c^ | none | ⨁⨁⨁◯ Moderate | 19 | 23 | - | The mean AOT versus video without motor content+ usual physiotherapy (Abdelhaleem 2021) INTERMEDIATE TERM was 0 | 0.35 lower (0.96 lower to 0.27 higher) |
| AOT versus video without motor content + usual physiotherapy (Abdelhaleem 2021) IMMEDIATE POST INTERVENTION (assessed with: Abilhands kids) | | | | | | | | | | | |
| 45 (2 RCTs) | not serious | not serious | not serious | serious^a^ | none | ⨁⨁⨁◯ Moderate | 22 | 23 | - | The mean AOT versus vdideo non motori + usual physiotherapy (Abdelhaleem 2021) IMMEDIATE POST INTERVENTION was 0 | 0.3 higher (0.28 lower to 0.89 higher) |
| AOT versus video without motor content+ usual physiotherapy (Abdelhaleem 2021) INTERMEDIATE TERM (follow-up: range 8 weeks to 24 weeks; assessed with: Abilhands kids) | | | | | | | | | | | |
| 46 (2 RCTs) | not serious | very serious^a,b^ | not serious | serious^a,b^ | none | ⨁◯◯◯ Very low | 23 | 23 | - | The mean AOT versus video without motor content+ usual physiotherapy (Abdelhaleem 2021) INTERMEDIATE TERM was 0 | 0.15 higher (0.43 lower to 0.73 higher) |
| VR (Wii, Xbox, non immersive) + usual physiotherapy versus usual care (Johansen 2020) (assessed with: mixed outcome measures) | | | | | | | | | | | |
| 222 (7 RCTs) | serious^d^ | very serious^a,e^ | not serious | serious^a^ | none | ⨁◯◯◯ Very low | 109 | 113 | - | The mean VR (Wii, Xbox, non immersiva) + usual physiotherapy versus usual care (Johansen 2020) was 0 | 0.89 higher (0.22 higher to 1.56 higher) |
| rTMS (alone or combined with CIMT) versus sham TMS (alone or combined with CIMT) (Elbanna 2019) (follow-up: range 2 days to 17 days; assessed with: AHA) | | | | | | | | | | | |
| 29 (2 RCTs) | serious^d^ | not serious | not serious | serious^f^ | none | ⨁⨁◯◯ Low | 15 | 14 | - | The mean rTMS (isolata o + CIMT) versus sham TMS (isolata o + CIMT) (Elbanna 2019) was 0 | mean 0.94 higher (0.42 higher to 1.46 higher) |

#### *Legend: RCT, randomized controlled trial; CI, confidence interval; ^a^ large standard deviation; ^b^ conflicting results among studies; ^c^ small sample; ^d^ moderate quality based on AMSTAR; ^e^ high hetereogenity; ^f^ small sample, though both studies report improvement and similar SD.*
